# GATK hard filtering: tunable parameters to improve variant calling for next generation sequencing targeted gene panel data

**DOI:** 10.1186/s12859-017-1537-8

**Published:** 2017-03-23

**Authors:** Simona De Summa, Giovanni Malerba, Rosamaria Pinto, Antonio Mori, Vladan Mijatovic, Stefania Tommasi

**Affiliations:** 1 IRCCS-Istituto Tumori “Giovanni Paolo II”, Molecular Genetics Laboratory, viale Orazio Flacco, 65, 70124 Bari, Italy; 2 Department of Neuroscience, Biomedicine and Movement Sciences, Section of Biology and Genetics, University of Verona, Strada Le Grazie 8, 37135 Verona, Italy

**Keywords:** NGS, Variant calling, Variant filtering, Targeted gene panel, SNV, Indel

## Abstract

**Background:**

NGS technology represents a powerful alternative to the standard Sanger sequencing in the context of clinical setting. The proprietary software that are generally used for variant calling often depend on preset parameters that may not fit in a satisfactory manner for different genes.

GATK, which is widely used in the academic world, is rich in parameters for variant calling. However the self-adjusting parameter calibration of GATK requires data from a large number of exomes. When these are not available, which is the standard condition of a diagnostic laboratory, the parameters must be set by the operator (hard filtering). The aim of the present paper was to set up a procedure to assess the best parameters to be used in the hard filtering of GATK. This was pursued by using classification trees on true and false variants from simulated sequences of a real dataset data.

**Results:**

We simulated two datasets, with different coverages, including all the sequence alterations identified in a real dataset according to their observed frequencies. Simulated sequences were aligned with standard protocols and then regression trees were built up to identify the most reliable parameters and cutoff values to discriminate true and false variant calls. Moreover, we analyzed flanking sequences of region presenting a high rate of false positive calls observing that such sequences present a low complexity make up.

**Conclusions:**

Our results showed that GATK hard filtering parameter values can be tailored through a simulation study based-on the DNA region of interest to ameliorate the accuracy of the variant calling.

**Electronic supplementary material:**

The online version of this article (doi:10.1186/s12859-017-1537-8) contains supplementary material, which is available to authorized users.

## Background

In the last decade, sequencing technologies, the so-called next generation sequencing (NGS), have delivered a step change in the ability to sequence genome, leading to a state of permanent evolution.

NGS platforms allow to detect mutations significantly reducing time and costs [1, 2]. Ion Torrent Personal Genome Machine (PGM) started to be distributed in 2011 [3] and thus to be used for the identification of genetic variants associated to human diseases [4, 5]. Indel detection, in particular, in homopolymer region have a high positive rate [6, 7] which have to be lowered for clinical applications [8, 9]. Thus the major challenge in NGS regards the correct manipulation of output data [10] assembling appropriate pipeline, including aligner and variant caller. Diverse algorithms for alignment have been compared in many studies [11, 12]. Caboche et al [13] compared different mapping algorithms with Ion Torrent data, in terms of computational requirement, mapper robustness, ability to map reads in repeated regions and behavior with mutated reference genome. They were able to optimize a benchmark procedure from whole genome sequencing of small genomes, highlighting the importance to evaluate mappers or to optimize parameters of a chosen mapper for a specific application. Moreover, variant calling pipelines have been compared in relation to different applications and platforms. Yeo et al. [14] optimized an indel detection workflow for BRCA1/2 PGM sequencing panel. They compared the proprietary software Torrent Suite and two open source variant callers, GATK and SAMtools. Their results showed that SNV detection was less problematic than indel identification using Torrent Suite. Moreover, they demonstrated how the combination of BWA or TMAP mappers and SAMtools is able to improve indel detection.

As demonstrated by these studies, the bioinformatic challenge on NGS data and, in particular, Ion Torrent data from targeted sequencing requires a lot of efforts in order to correctly identify the best analysis pipeline.

GATK is a well-known toolbox for NGS data analysis. Variant Quality Score Recalibration (VQSR) step generate an adaptive model based on metrics, such as strand bias, from true variants. Thus it could be possible to calculate if a variant is true or false. However, this step could be used only for whole genome data or for dataset including more than 30 exomes. For targeted gene panels, GATK’s Best Practices suggest to set up hard filters specific for the study. In the present study, we compared variant calling results of GATK pipeline including the use of hard filtering, suggested by GATK’s Best Practices, and the proprietary Torrent Suite Variant Caller regarding a custom panel including 11 genes. Then, we focused on two simulated datasets (100 replicates for each dataset), with high and low coverage, and then we processed the raw variants called by GATK to set parameters of quality in order to increase the number of true variants and decrease the number of false variants.

## Methods

### Real dataset

A dataset of 26 metastastic melanoma formalin-fixed paraffin embedded (FFPE) samples was studied. Exonic regions of a panel of 11 genes were sequenced with Ion Torrent PGM. Sequencing data were analyzed using 2 standard pipelines including either the Torrent Suite (TMAP 4.0.6 aligner and Torrent Variant Caller version 4.2-18 (TVC) with the parameters “somatic” and “high stringency” switched on) or the GATK suite for few variants (bwa aligner and GATK programs; see GATK pipeline section for details).

### Simulated datasets

The above reported panel of 11 genes was devised to identify sequence variants in the coding regions. When the present study was conceived we prepared a catalog of known variants and their allele frequency using the information from the real 26 sequenced individuals. The simulation-based study was then set up using a reference dataset of 26 individuals with randomly assigned variants according to the catalog of known variants. The simulated genotypic profiles of each individual were recorded. For every sample it was prepared by simulation a file (fasta format) containing the amplicon sequences that were modified to introduce the assigned variants, repeated 2 times (since humans are diploids) in both the forward and in the reverse sequence. The dataset of 26 fasta files was then processed with the ART simulator [15] to generate files (FASTQ format) similar to those produced by a sequencer of new generation, mimicking its features and biases. For every sample the ART simulator was launched twice under the hypothesis of a sequencing depth of 20x (low coverage, LC) and 100x (high coverage, HC), respectively. The 26 files were then processed using a standard pipeline for variant calling that includes the aligner BWA and the GATK suite of programs (see GATK pipeline section). All the still unfiltered variants were tagged as true (actually present in the fasta file of the simulated subject) or false (not present in the fasta file and therefore representing the product of an erroneous call by the bioinformatic variant calling process) variants. Thus one hundred independent datasets of 26 individuals were simulated for a total of 5200 simulated samples. By this approach we tried to simulate several times the most likely scenario (number of samples per dataset, amplicons used, selected genes, reported variants) resembling to the real one.

### GATK pipeline

We followed the Toolkit for Genome Analysis (GATK, https://software.broadinstitute.org/gatk/) [16] recommendations of DNAseq best practices for calling variants. Hence the following software were used: BWA-mem (http://bio-bwa.sourceforge.net/) for sequence alignment [17] and GATK 3.4 software for the later steps. In more detail, sequences underwent the following steps: 1) alignment to the human genome reference version hg19, 2) realignment around Indels, 3) base recalibration and 4) variant discovery (using the haplotype caller function in ERC mode) without been marked for duplicates.

The discovered variants were hard filtered after having selected the rules to setup the filters from the classification trees as described in the “Filters for the variant calling” and “Classification trees” sections.

### Filters for the variant calling

Hard filtering evaluated 7 standard GATK filters (BaseQRankSum, ClippingRankSum, DP, MQ, GQ, MQRankSum, ReadPosRankSum, see Additional file 1 for a description) and 3 filters (FS, ADT, ADTL) that were not present in the standard GATK vcf output files. FS is the *p* value from the contingency table of the number of reads calling the alleles at the variant site on either the DNA strands (forward and reverse). ADT and ADTL evaluate imbalances in calling the reference and the alternative allele (ADT), also depending on the amount of reads that map on the variant locus (ADTL). ADT and ADTL are defined as follows:

$$ \begin{array}{l}\mathrm{ADT}=\frac{\left|\left(\mathrm{AD}1-\mathrm{AD}2\right)\right|}{\mathrm{AD}1+\mathrm{AD}2}\\ {}\mathrm{ADT}\mathrm{L}=\mathrm{lo}{\mathrm{g}}_{10}{\left(\mathrm{AD}1+\mathrm{AD}2\right)}^{*}\mathrm{ADT}\end{array} $$

where AD1 and AD2 are the number of unfiltered reads calling the reference and alternate allele, respectively.

Descriptive statistics were performed using R 3.2.3 and the kruskal.test function, and ROCR library version 1.0-7.

### Classification trees

Every filter was included into classification trees to target the filter rules that better discriminate the true variants (listed by GATK and also present in the simulated sample) from false variants (proposed by GATK but not present simulated in the sample under examination). These rules have been then used for the hard filtering. Eight classification trees were generated to investigate separately SNV and INDEL, homozygotes and heterozygotes (as shown by GATK) under a simulated coverage of 20x or 100x. For every tree we extracted the selected filters and their threshold values in order to use them in the phase of hard-filtering. We extracted the filters that were listed starting from the root node of the classification tree to the next 2 consecutive daughter nodes (for a maximum of 7 filters). The filters selection for each classification tree was made as follows. Comparing every daughter node with the root node we targeted the nodes that 1) contained at least 10% of the true calls and 2) where the ratio of the number of true calls and false calls was greater than 3. To minimize the number and complexity of the filter rules we then considered for each targeted node l (lower node) the upper connected node u (upper node) closer to the root node. We then selected the node u instead of the node l when 1) the node l contained less than 80% of the true calls or 2) when containing more than 70% of false calls of node u (i.e. reduction of at least 30% of false calls), respectively. Once the relevant nodes were selected we extracted the filter rules starting from the root node toward each of the finally selected daughter nodes.

Classification trees were produced using the library rpart (version 4.1-9) of the R package (version3.1.2)

## Results

### Results in the real dataset: GATK vs TVC

In the first part of this study we looked at the results obtained using 2 different standard pipelines on the same set of sequences. TVC is the software use for targeted sequencing in bundle with the Ion Torrent sequencer. GATK is considered the “gold standard” in managing large NGS data (i.e. exomes and genomes) and can be used for targeted sequencing. For this reason, we compared TVC calls with those produced by GATK 3.4. TVC called 399 variants in the entire dataset, 73 of which were shared with GATK that detected 83 SNVs. Then we performed the VCF files, which are the output files of both TVC and GATK, focusing on some Parameters Of sequencing Quality. In particular, DP (Coverage) and AF (Allele Frequency) tags were shared by VCF outputs. TVC calls showed mean, DP and AF values of 1658.13 and 0.15, respectively. The 73 SNVs called by both TVC and GATK showed higher mean. This observation could suggest that shared SNVs are true positive calls, having higher coverage and quality by depth values. It is noteworthy that mean POQ values of the variants called only by GATK were similar to TVC calls (DP: 1795.33; AF: 0.16). SNVs identified only by TVC showed lower mean DP and AF values than shared variations (663.75, 0.08, respectively). Such results suggest that SNVs called by TVC may be enriched of many false positives. Indel calling is a highly debated problem when we refer to Ion Torrent data. In a very preliminary way, we evaluated mean DP and AF values of 107 Indels identified by GATK, which displayed similar values to those of shared SNVs (3814.58, 0.16, respectively). No indel was detected by TVC.

### High-coverage and low-coverage simulated datasets: descriptive statistics

To better explore GATK variant calling and to try to tune the hard filtering parameters (filters), we performed a simulation-based study, as described in the “Methods” section. In the High-Coverage (HC) dataset, GATK identified 91115 unfiltered SNVs and 81640 unfiltered Indels. It is noteworthy that 98.49% and only 21.23% of SNVs and Indels were true variants, respectively. In the Low-Coverage (LC) dataset, 113246 and 88145 unfiltered SNVs and unfiltered Indels were respectively called. As expected, we found that the percentage of true variants was lower in the LC dataset (84.95% for SNVs and 8.68% for Indels) (Table 1).

**Table 1:** Overall GATK unfiltered alterations identified in HC and LC dataset (100 replicates of a dataset of 26 individuals and 11 genes)

	TV	FV
HC dataset		
*Indels*	17,359	64,281
*SNVs*	89,743	1,372
LC dataset		
*Indels*	7,656	80,489
*SNVs*	96,203	17,043

*TV* true variants, *FV* false variants

We observed that 3.9% of SNVs and 53.8% of Indels were homozygous in the HC dataset whereas 14.8% of SNVs and 51.9% of Indels were homozygous in the LC dataset.

We investigated the distribution of the values of the individual GATK filters, namely BaseQRankSum, ReadPosRankSum, ClippingRankSum, DP, MQ, MQRankSum, and GQ (see Additional file 1 for details) between true and false variants. Table 2 reports the descriptive statistics for the HC dataset and shows that BaseQRankSum, ReadPosRankSum and DP display a statistically significant difference both in SNVs and Indels. MQRS showed a statistically significant difference in Indels only.

**Table 2:** Descriptive statistics of GATK filters in the HC dataset, stratifying calls by type (SNV/Indels). Data are displayed as mean ± sd

	SNVs			Indels		
	*TV* *mean ± sd*	*FV* *mean ± sd*	*p-value*	*TV* *mean ± sd*	*FV* *mean ± sd*	*p-value*
BQRS	0.11 ± 0.03	-0.6 ± 0.5	<0.0001	0.28 ± 0.12	0.15 ± 0.05	<0.0001
RPRS	-0.067 ± 0.05	0.05 ± 0.4	0.0009	-0.74 ± 0.22	0.23 ± 0.05	<0.0001
CRS	0.0007 ± 0.02	-0.009 ± 0.29	0.72	0.001 ± 0.07	0.007 ± 0.03	0.6
DP	96.61 ± 0.58	49.25 ± 5.06	<0.0001	109.4 ± 9.57	96.01 ± 0.1	<0.0001
MQ	60 ± 0	59.99 ± 0.07	-	60 ± 0	60 ± 0	-
MQRS	-0.03 ± 0.02	-0.05 ± 0.28	0.3	-0.02 ± 0.09	-0.21 ± 0.04	<0.0001
GQ	99 ± 0	79.15 ± 12.06	-	99 ± 0	73.16 ± 2.7	-

The mean value is the mean value of the median value from each of the 100 replicates

*BQRS* BaseQRankSum, *RPRS* ReadPosRankSum, *CRS* ClippingRankSum, *DP* depth of coverage, *MQ* MappingQuality, *MQRS* MappingQualityRankSum, *GQ* genotype quality, *TV* true variants, *FV* false variants

Table 3 reports the descriptive statistics for the LC dataset and shows a statistically significant difference for all the GATK filters with the exception of MQ both in SNVs and Indels subsets. Of note that GQ always (= the median value of each of the 100 replicates) presented a value equal to 99 for the true variants. It is intriguing that in the case of SNV subset, DP presents the lowest values in the TVs compared to FVs even the difference is relatively small.

**Table 3:** Descriptive statistics of GATK filters in the LC dataset, stratifying calls by type (SNV/Indels). Data are displayed as mean ± sd

	SNVs			Indels		
	*TV* *mean ± sd*	*FV* *mean ± sd*	*p-value*	*TV* *mean ± sd*	*FV* *mean ± sd*	*p-value*
BQRS	0.02 ± 0.02	-0.27 ± 0.12	<0.0001	0.16 ± 0.16	0.01 ± 0.03	<0.0001
RPRS	-0.19 ± 0.03	-0.31 ± 1.1	<0.0001	-1.29 ± 0.3	0.04 ± 0.04	<0.0001
CRS	-0.02 ± 0.02	-0.05 ± 0.07	<0.0001	-0.004 ± 0.12	-0.04 ± 0.01	0.001
DP	19.97 ± 0.17	22.72 ± 1.3	<0.0001	21.83 ± 1.97	20.24 ± 0.42	<0.0001
MQ	60 ± 0	60 ± 0	**-**	60 ± 0	60 ± 0	**-**
MQRS	-0.06 ± 0.01	-0.1 ± 0.08	<0.0001	-0.15 ± 0.15	-0.1 ± 0.04	0.03
GQ	99 ± 0	20.04 ± 2.9	**-**	99 ± 0	17.94 ± 0.92	**-**

The mean value is the mean value of the median value from each of the 100 replicates

*BQRS* BaseQRankSum, *RPRS* ReadPosRankSum, *CRS* ClippingRankSum, *DP* depth of coverage, *MQ* MappingQuality, *MQRS* MappingQualityRankSum, *GQ* genotype quality, *TV* true

The performance of all individual filters to discriminate between true and false variants was summarized by estimating the area under the ROC curve (AUC). Table 4 reports that ADT and GQ showed the best performance. Additionally every filter showed a better performance for SNVs rather than Indels (Additional file 2).

**Table 4:** Perfomance of the individual filters evaluated to discriminate between true and false variants by the AUC values from ROC curve, grouped by type of variants (SNV or Indel) and status of the genotype call (homozygote or heterozygote) according to the depth of sequencing (LC or HC dataset)

	SNV		Indel	
	*Homo*	*Het*	*Homo*	*Het*
HC dataset				
BQRS	0.73	0.53	0.5	0.53
RPRS	0.57	0.61	0.52	0.68
CRS	0.5	0.51	0.53	0.5
DP	0.79	0.8	0.76	0.6
MQ	0.55	0.5	0.6	0.63
MQRS	0.52	0.53	0.58	0.58
GQ	0.65	0.95	0.77	0.77
ADT	0.96	0.8	0.56	0.94
ADTL	0.8	0.77	0.72	0.94
FS	0.51	0.62	0.51	0.54
LC dataset				
BQRS	0.58	0.65	0.5	0.52
RPRS	0.53	0.5	0.54	0.74
CRS	0.52	0.5	0.51	0.51
DP	0.63	0.67	0.52	0.62
MQ	0.51	0.54	0.54	0.52
MQRS	0.52	0.52	0.51	0.5
GQ	0.79	0.99	0.53	0.97
ADT	0.98	0.99	0.5	0.92
ADTL	0.67	0.98	0.54	0.98
FS	0.5	0.54	0.5	0.54

It could be noticed that correctly called alterations showed a higher coverage than false variants, highlighting the importance of this parameter.

### Classification trees

We performed the analyses of either SNVs or Indels subsets stratified by genotype, in HC and LC datasets. Classification trees (Additional file 3) allowed to set a series of filter rules for each of the 2 type of sequence alteration. Table 5 shows the parameters and threshold values to be used for hard-filtering been extracted from the classification trees.

**Table 5:** Parameters and their thresholds selected by regression trees

Sequencing depth	Variant type	Genotype by GATK	Filter rule
20x	SNV	homozygous	ADT > =0.98
20x	SNV	heterozygous	ADT < 0.55
20x	INDEL	homozygous	N/A (*)
20x	INDEL	heterozygous	ADT < 0.26 & GQ > =98.5 & DP > =23.5 & MQ > =59.5 ADT < 0.26 & DP > 23.5 & ReadPosRankSum < -1.55
100x	SNV	homozygous	ADT > =0.96
100x	SNV	heterozygous	GQ > =68.5
100x	INDEL	homozygous	ADTL > =5.08
100x	INDEL	heterozygous	ADT < 0.15 & MQ > =59.91 & GQ > =98.5

(*): no reliable filters were selected by classification trees

Notably, the classification tree did not select any reliable filter for homozygous Indels in the LC dataset (Table 6).

**Table 6:** Results by the application of selection parameters and their thresholds on simulated datasets

	TV N (%)	FV N (%)	Variant selected by hard filtering %
HC dataset			
Homo SNVs			
*Overall*	2,382 (66.6)	1,195 (33.4)	93.9
*Selected*	2,238 (98.6)	31 (1.3)	
Het SNVs			
*Overall*	87,361 (99.8)	177 (0.2)	98.6
*Selected*	86,166 (99.9)	24 (0.03)	
Homo indels			
*Overall*	54 (0.12)	43,871 (99.8)	27.7
*Selected*	15 (75)	5 (25)	
Het indels			
*Overall*	17.305 (45.8)	20,410 (54.1)	84.6
*Selected*	14,646 (94)	935 (6)	
LC dataset			
Homo SNVs			
*Overall*	2,084 (12.38)	14,721 (87.6)	96.9
*Selected*	2,020 (92.2)	171 (7.8)	
Het SNVs			
*Overall*	95,119 (97.6)	2,322 (2.3)	99.4
*Selected*	94,602 (99.9)	80 (0.08)	
Homo indels			
*Overall*	154 (0.4)	45623 (99.6)	100
*Selected*	154 (0.4)	45623 (99.6)	
Het indels			
*Overall*	7,502 (17.6)	34,889 (82.3)	43
*Selected*	3,226 (99.1)	27 (0.8)	

% have to be intended as the percentage of unfiltered variants for “overall”calls and as the percentage of alterations which were not filtered out in the hard filtering process for “selected”calls; % of selection indicates the amount of variants selected from the total callset. *TV* true variants, *FV* false variants

We then explored the sequence of the flanking regions of each type of alterations, in particular we observed that short homopolymeric strings are recurrent and therefore partly responsible for false positive calls (Table 7, Additional file 3).

**Table 7:** Homopolymeric sequences flanking false positive variants

	Chr	Position	Flanking sequence	N° of occurrences
HC dataset				
Homo SNVs	chr10	131565164	CCGGT**T**GGGGA	77
	chr3	178921420	GGACT**G**TTTTT	73
Het SNVs	chr13	48919347	TAAAC**A**TTTTA	63
	chr3	178937372	CTTGG**T**AAAAG	9
Homo Indels	chr4	55602995	AGAGC**C**AAAAA	1842
	chr10	89693016	AAGTT**A**TTTTT	1802
Het Indels	chr13	48955363	AGTTA**C**TTTTT	2175
	chr3	178941853	CTATC**C**TTTTT	1678
LC dataset				
Homo SNVs	chr2	204736165	GGGTT**G**TTTTT	334
	chr13	48954225	GGTAA**A**TTTTT	241
Het SNVs	chr7	140534584	AAACA**G**AAAAA	32
	chr13	48955464	CTTTG**A**TTTTT	20
Homo Indels	chr7	140481508	AACAG**T**AAAAA	1153
	chr7	140481513	TAAAA**A**AGTCA	1084
Het Indels	chr3	69915434	TAAAG**G**AAAAA	1202
	chr10	89693016	AAGTT**A**TTTTT	1107

Variant locus is on the 6^th^ nucletide (bold) of the 11 nucleotide string (flanking sequence)

## Discussion

It is well known that there are many technical challenges involved in getting an accurate variant calling procedure of NGS data including the bioinformatic analysis. A number of tools based on complex statistical models has been developed but many concerns related to their performance remain still open. Since the number of the called variants varies from software to software, typically more than one computer program is then used. If the variant is actually called by all the programs then its support increases. However, the problem occurs when the variant is called only by some programs, raising the suspicion that it is not true. NGS is now applied in many fields. We were interested in studying the case of targeted sequencing of small set of genes when using a common NGS platform such as Ion Torrent. When analyzing a few variants (as in the case of a panel of genes rather than an exome) the GATK guides suggest to use filters that must be set by the user (hard-filtering) rather than the adaptive filtering that needs a high number of variants to work properly. Under these conditions (which are very common since many laboratories developed their own panel of specific genes to study the association with a specific phenotype) it becomes important to tailor the filters to call the true variants on the specific design. It is likely that different panels of genes and even different designs for the same panel of genes require a different setup of filters. We therefore tried to explore through a simulation-based study the outcomes that the pipeline for the variant calling may encounter. We were interested in the study of a specific scenario made up a group of individuals with a specific sequencing design. We measured the performance inthe calling true variants for each of the filters that can be set when working with hard-filtering. Hence we used classification trees on a large data simulated dataset of true and false variants.

In the present paper, we studied several standard and non standard GATK filters to be used for hard-filtering in the context of a targeted gene panel sequencing. Firstly, we analyzed a real dataset coming from the sequencing of an Ion Torrent targeted gene panel observing a high discrepancy between TVC and GATK, particularly for Indels, suggesting that such type variants are even difficult to be detected by the present bionformatic tools. In fact the importance to define a “gold standard” dataset to test variant calling methods is a very hot topic. Recently, “synthetic” matched tumor/normal samples was created for comparing performances of popular variant callers in detection of “somatic” SNVs. However, even if they had the advantage to refer to NIST-GIAB [18] as gold standard, authors could not discriminate “somatic” SNVs from germline background, an important issue when studying tumors, and moreover the batch that they purchased was not the same used for NIST-GIAB [19]. In the present study, we decided to simulate two datasets, each with a different coverage and carrying alterations found in real data. Notwithstanding this investigation did not simulate tumor heterogeneity, GATK variant calling was tested both in a relative high coverage and low coverage conditions.

Recently, Vanni et al [20] highlighted the discrepancy between TVC and GATK, which was also observed in our study, excluding indel calls from comparison. They considered Phred score ranging 5–30 to mark low-quality variants. Our results show that such approach could not be enough to have a high quality GATK call set. In detail, we evidenced that different parameters could be tuned depending on type of mutations and genotypes suggested. In a previous study, authors focused on the detection of parameters that could allow to improve indel detection [7]. They focused on two parameters regarding the frequency of reference and alternate alleles and the variance of the width of inserted/deleted sequences. The first parameters is similar to our ADT and ADTL filters, which were involved in the step of selection for the reduction of false positives. They observed that the numbers of false positive regarded in particular indel in homopolymeric regions. In a similar way we observed that flanking regions, were homopolymeric in a high number of false positive calls. It is important to reduce errors in these regions because they occur in genomic regions where the occurrence of true alterations is also higher [21]. Variant calling of TVC is improving but Indels are still a problem and thus parallel pipeline with opportune set up and filters could be helpful to solve this question, with particular attention on type of platform used for sequencing and on type of design (e.g., exome, targeted gene panel). Carson et al [22] recently demonstrated how DP and GQ filters could be able to enhance sensitivity and specificity in whole exome sequencing data. They tested different thresholds and showed that over a certain threshold accuracy reached a plateau and notably they demonstrated that VQSR is not enough to improve variant calling. Indeed, they concluded that, also when VQSR could be applied, opportune hard filtering strategies need to be set up. Our intent was not to target the precise hard filter parameter values and our results have to be intended as suggestion in handling data coming from targeted gene panel sequencing in order to optimize GATK variant calling outputs.

We observed that hard filtering was able to reduce the number of unfiltered false positives, with a different efficiency between SNVs (higher) and Indels (lower). True Indels were hard to be filtered and the performance of filtering was generally lower than in the case of the SNVs (i.e. high loss of true variants and high abundance of false variants; see Table 6). We also observed that the very majority to the unfiltered Indels called at the homozygous status is represented by false variants. Hence there are some regions of the DNA reference sequence that are prone to be recognized as carrying Indels by the bioinformatic pipeline. Our preliminary investigations show that these regions often contain low complexity sequences (for instance a short sequence made up of the same base). A good strategy would be to train in advance the program that operates in the indel parameter recalibration phase to recognize these regions but this hypothesis needs to be investigated in more detail. The reader should note that even if some NGS technologies are known to read with difficulty the DNA regions having low complexity, we are here asserting that the call errors of the variants in the low complexity regions are due to the bioinformatic analysis and not to sequencing errors as we worked with data produced by the simulator and not by a NGS sequencer. So, in the real world, regions with low complexity sequences are doubly condemned to possible abundant errors due to both sequencing and the following bioinformatics analysis.

Some results were quite different from the expected. In particular, we observed that they were detected (unfiltered and then filtered) more SNVs in the case of low coverage (see Table 6, Het SNVs) than in high coverage. Of note that in such cases the rate of false variant is very small even for the unfiltered variants. However, the rate of false variant is about 10 folds greater in the LC dataset. Therefore we hypothesize that variant calling process is more sensitive because of less specific when analyzing dataset with a low coverage.

In general terms, as already known, the Indels are more difficult to be analyzed than the SNVs and a deeper sequencing helps to improve the performance of filtering the true variants. However, such a performance could vary since complexity of the sequence changes along the sequence itself. In the case of targeted sequencing, our suggestion is to study in advance the region that we will be sequenced in order to evaluate the performance of the variant calling procedure over such regions in order to figure out the most problematic areas to be treated with caution. We also suggest the use of simulations based on the specific target region which can help to calibrate the filters for the specific problem. We argue that it could be useful to set specific filters for different regions and for different known variants.

## Conclusions

The results of our study showed that filters could be correctly tuned according to coverage and type of alterations. Moreover, it could be useful to test by appropriate simulations the design of amplicon gene panels to gain a priori knowledge of the possible issues in variant calling by GATK.

## Additional files

Additional file 1: Technical definitions of Parameters of Sequencing Quality. (PDF 107 kb)

Additional file 2: Performance of all individual filters to discriminate between true and false variants estimated by the area under the ROC curve (AUC). (PDF 595 kb)

Additional file 3: Classification trees to set filter rules. (PDF 9 kb)

## Abbreviations

SNV: Single nucleotide variants
