# Supplementary material for: GATK hard filtering: tunable parameters to improve variant calling for next generation sequencing targeted gene panel data
Source: BMC Bioinformatics. 2017 Mar 23;18(Suppl 5):119. doi: 10.1186/s12859-017-1537-8 (PMC5374681; doi:10.1186/s12859-017-1537-8)
Supplement: Supplementary file 1 — Technical definitions of Parameters of Sequencing Quality. (PDF 107 kb) [file 12859_2017_1537_MOESM1_ESM.pdf]

Supplementary File 1

Technical definitions of Parameters of Sequencing Quality

Parameter	Definition*
**BaseQRankSum**	It compares the base qualities of the data supporting the reference allele with those supporting any alternate allele
**ReadPosRankSum**	This variant-level annotation tests whether there is evidence of bias in the position of alleles within the reads that support them, between the reference and alternate alleles
**ClippingRankSum**	This variant-level annotation tests whether the data supporting the reference allele shows more or less base clipping (hard clips) than those supporting the alternate allele
**DP**	This annotation is used to provide counts of read depth at two different levels, with some important differences. At the sample level (FORMAT), the DP value is the count of reads that passed the caller's internal quality control metrics (such as MAPQ > 17, for example)
**MQ**	The count of all reads that have MAPQ = 0 across all samples
**MQRankSum**	The count of all reads that have MAPQ = 0 for each sample
**GQ**	Conditional genotype quality, encoded as a phred quality −10log10p
**AD**	Also known as the allele depth, this annotation gives the unfiltered count of reads that support a given allele for an individual sample. The values in the field are ordered to match the order of alleles specified in the REF and ALT fields: REF, ALT1, ALT2 and so on if there are multiple ALT alleles.

*As reported on the GATK website (https://www.broadinstitute.org/gatk/) as of 2nd March 2016
