# Supplementary material for: GATK hard filtering: tunable parameters to improve variant calling for next generation sequencing targeted gene panel data
Source: BMC Bioinformatics. 2017 Mar 23;18(Suppl 5):119. doi: 10.1186/s12859-017-1537-8 (PMC5374681; doi:10.1186/s12859-017-1537-8)
Supplement: Supplementary file 2 — Performance of all individual filters to discriminate between true and false variants estimated by the area under the ROC curve (AUC). (PDF 595 kb) [file 12859_2017_1537_MOESM2_ESM.pdf]

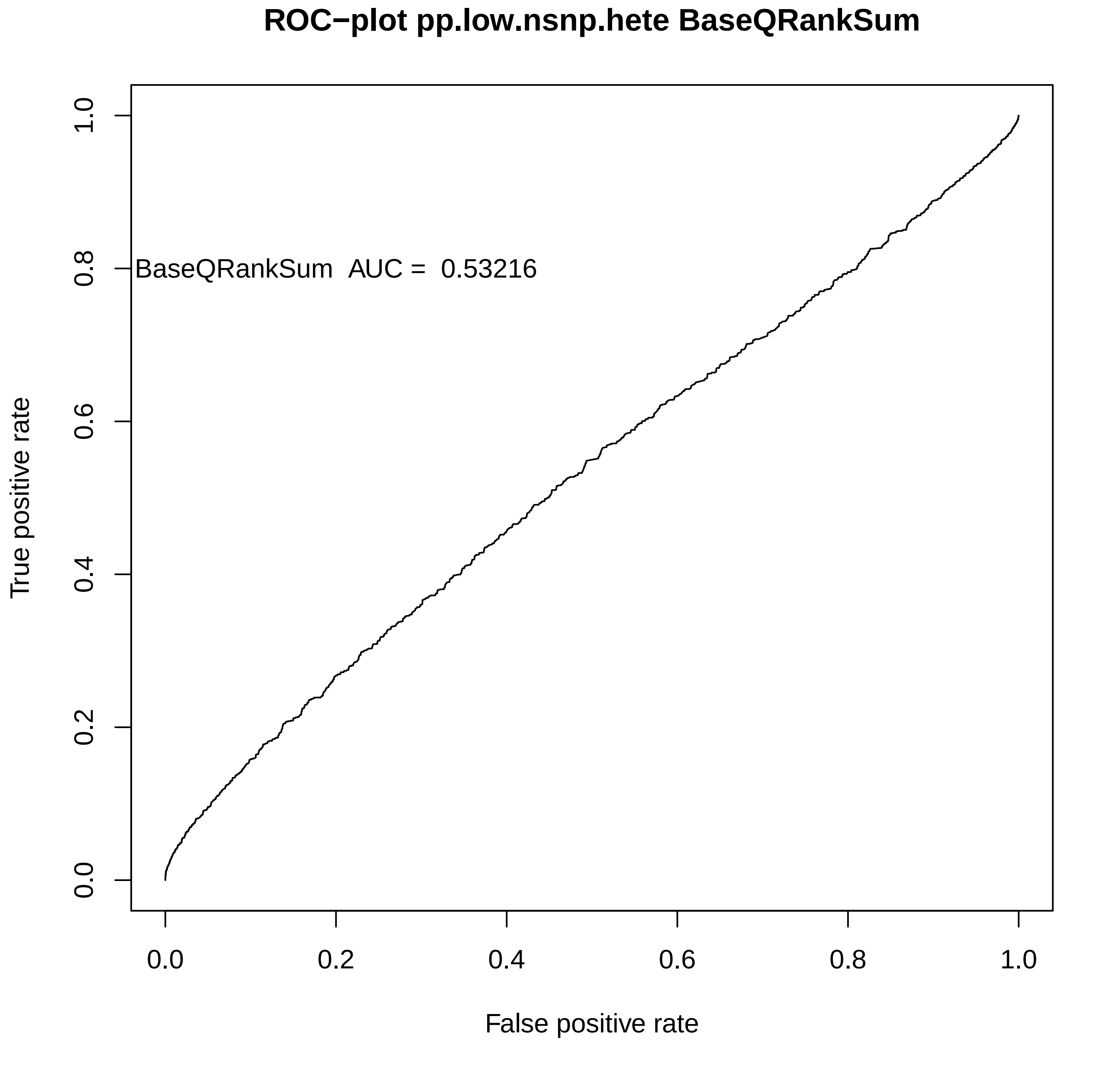
ROC−plot pp.low.nsnp.hete BaseQRankSum
BaseQRankSum AUC = 0.53216
True positive rate
False positive rate
0.0
0.2
0.4
0.6
0.8
1.0

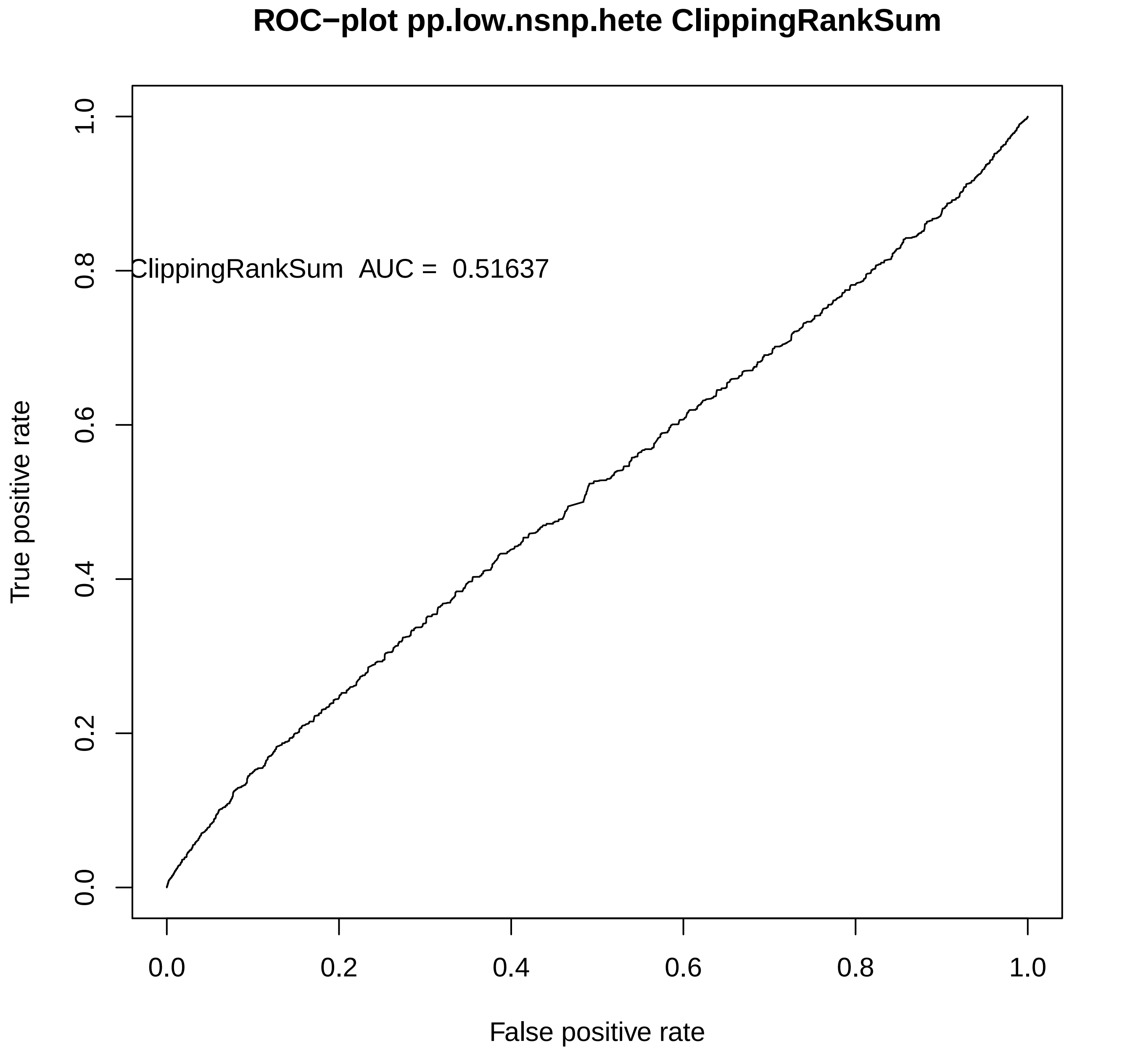
ROC−plot pp.low.nsnp.hete ClippingRankSum
ClippingRankSum AUC = 0.51637
True positive rate
False positive rate
0.0
0.2
0.4
0.6
0.8
1.0

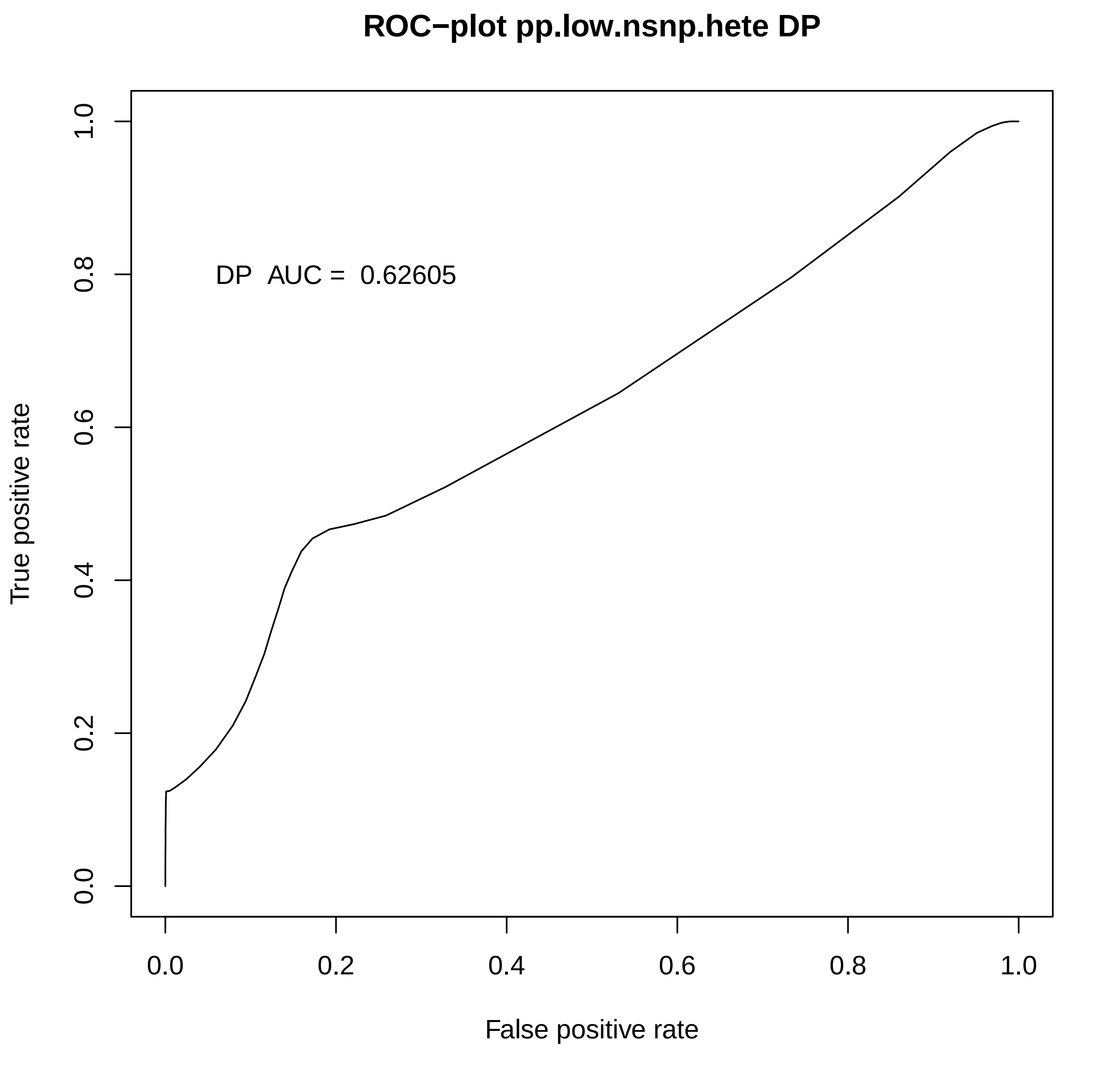
ROC−plot pp.low.nsnp.hete DP
DP AUC = 0.62605
True positive rate
False positive rate
0.0
0.2
0.4
0.6
0.8
1.0

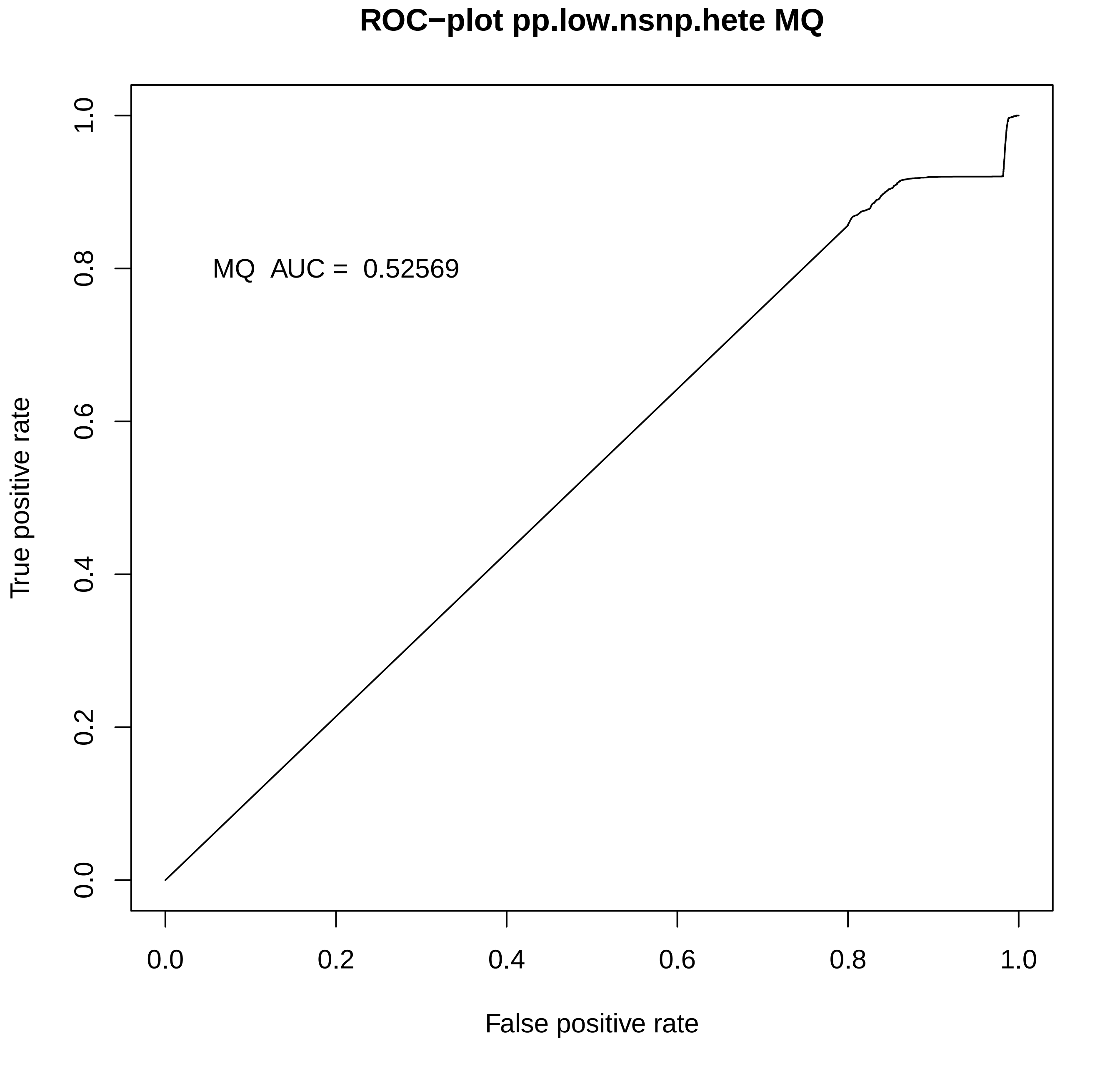

ROC−plot pp.low.nsnp.hete MQ
MQ AUC = 0.52569
True positive rate
False positive rate
0.0
0.2
0.4
0.6
0.8
1.0

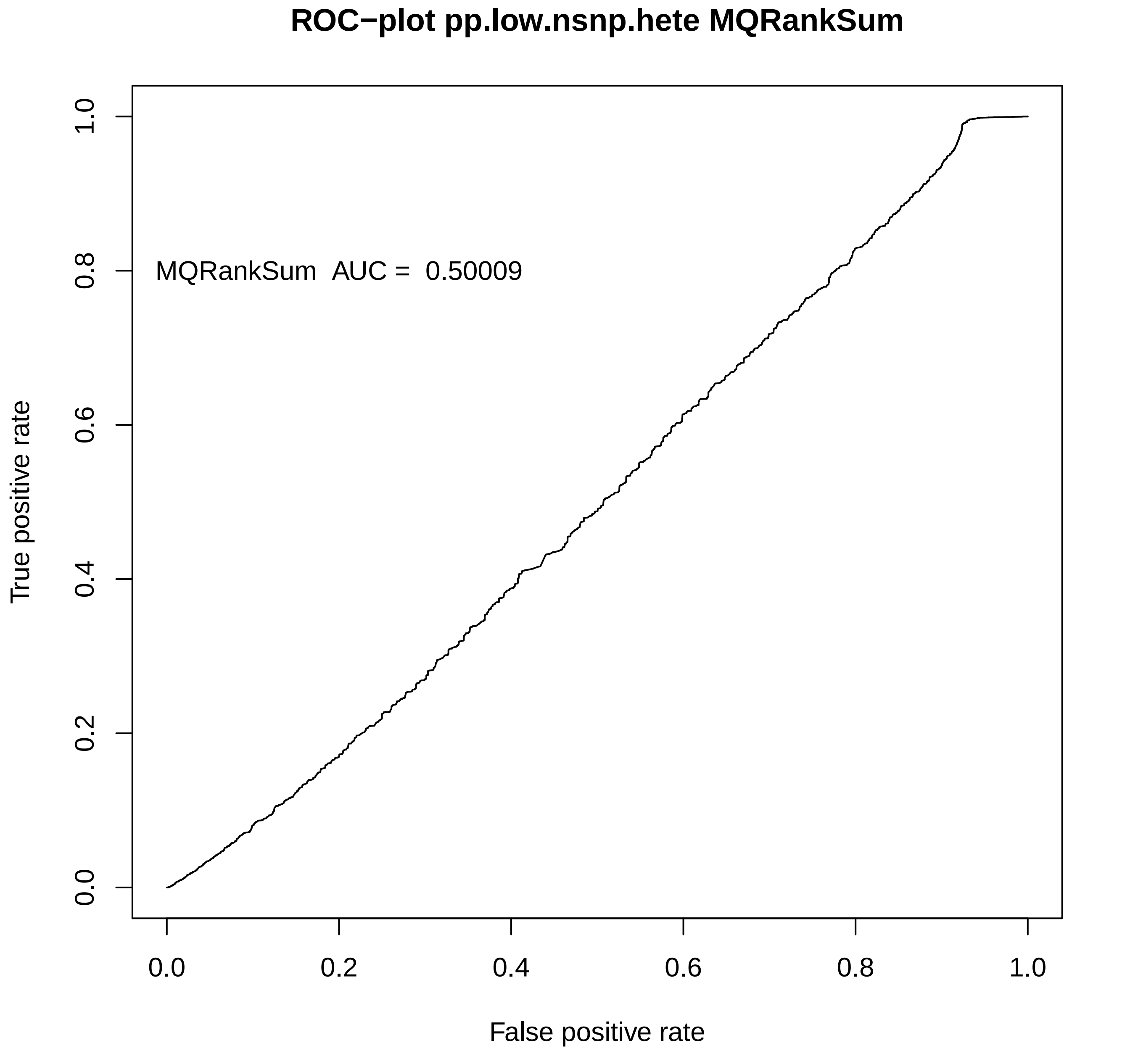
ROC−plot pp.low.nsnp.hete MQRankSum
MQRankSum AUC = 0.50009
True positive rate
False positive rate
0.0
0.2
0.4
0.6
0.8
1.0

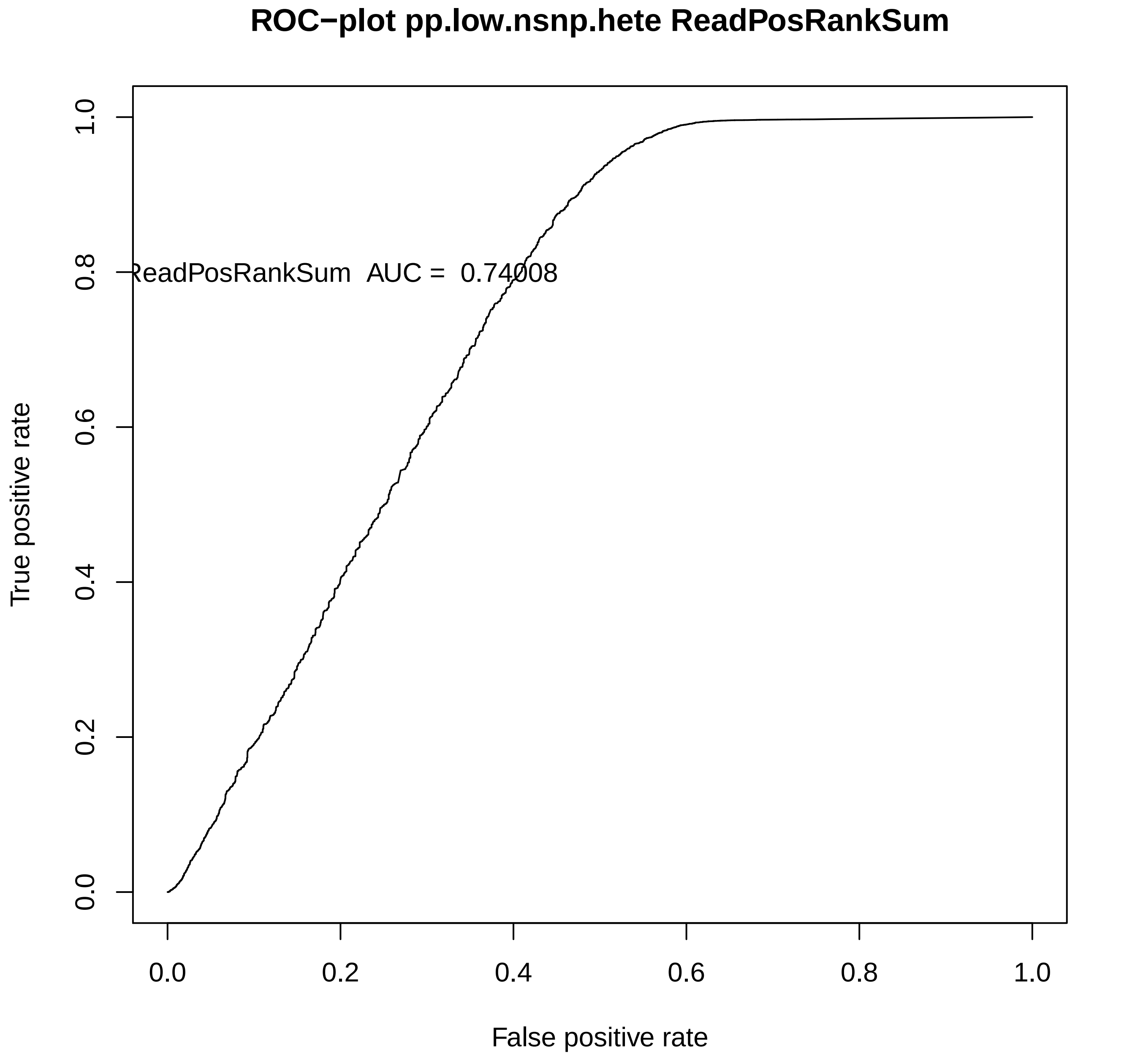
ROC−plot pp.low.nsnp.hete ReadPosRankSum
ReadPosRankSum AUC = 0.74008
True positive rate
False positive rate
0.0
0.2
0.4
0.6
0.8
1.0

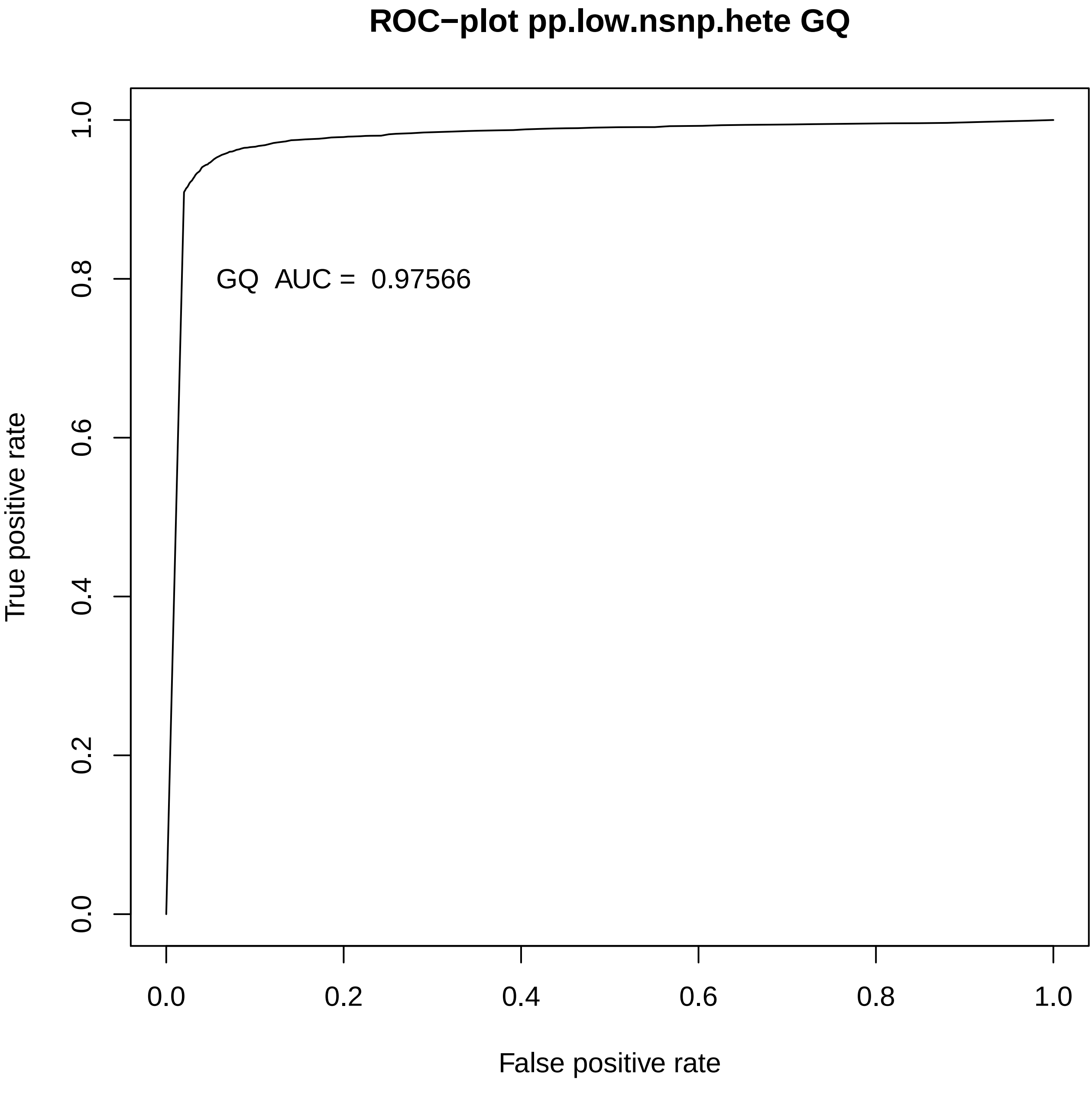
ROC−plot pp.low.nsnp.hete GQ
GQ AUC = 0.97566
True positive rate
False positive rate
0.0
0.2
0.4
0.6
0.8
1.0

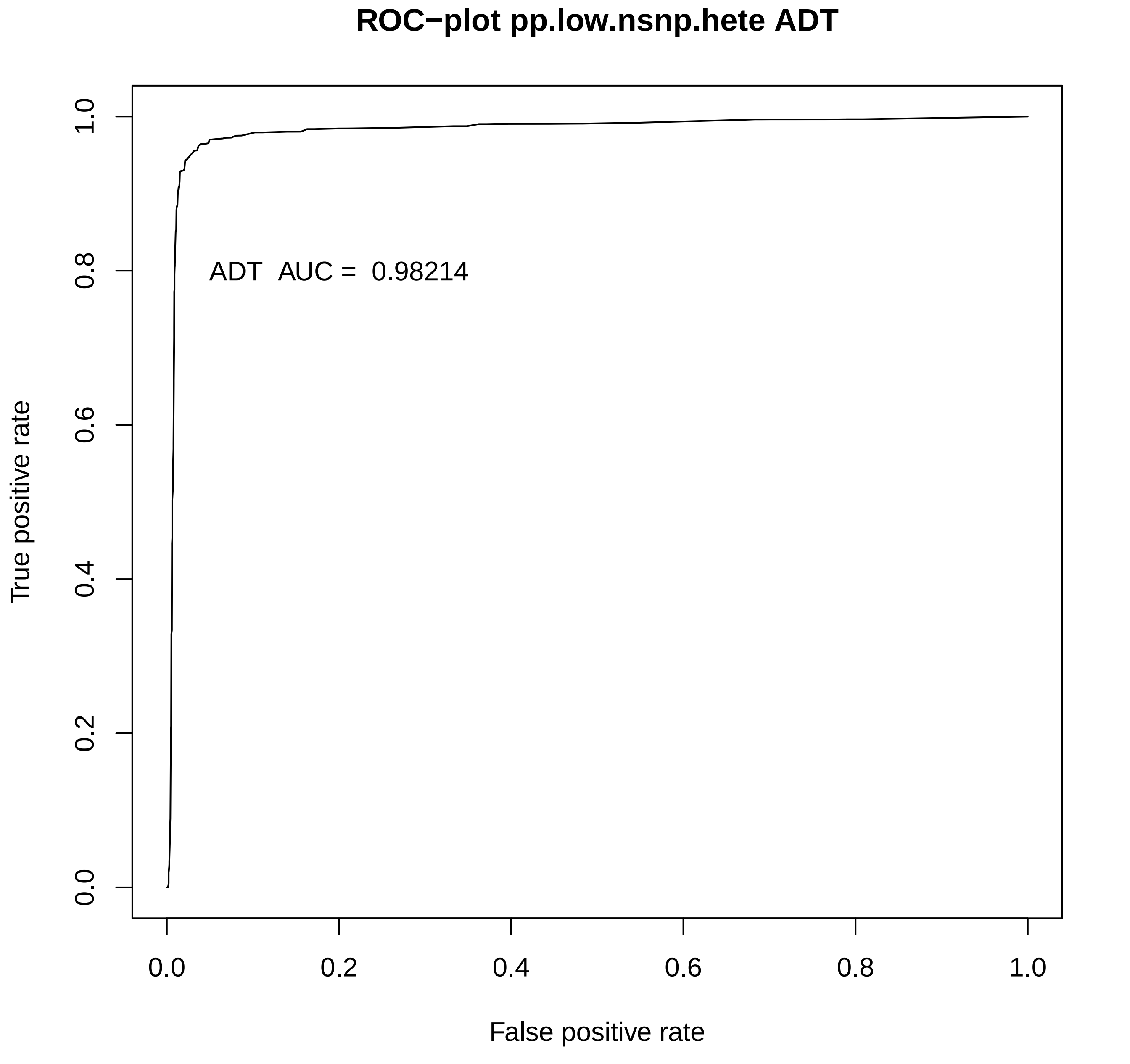
ROC−plot pp.low.nsnp.hete ADT
ADT AUC = 0.98214
True positive rate
False positive rate
0.0
0.2
0.4
0.6
0.8
1.0

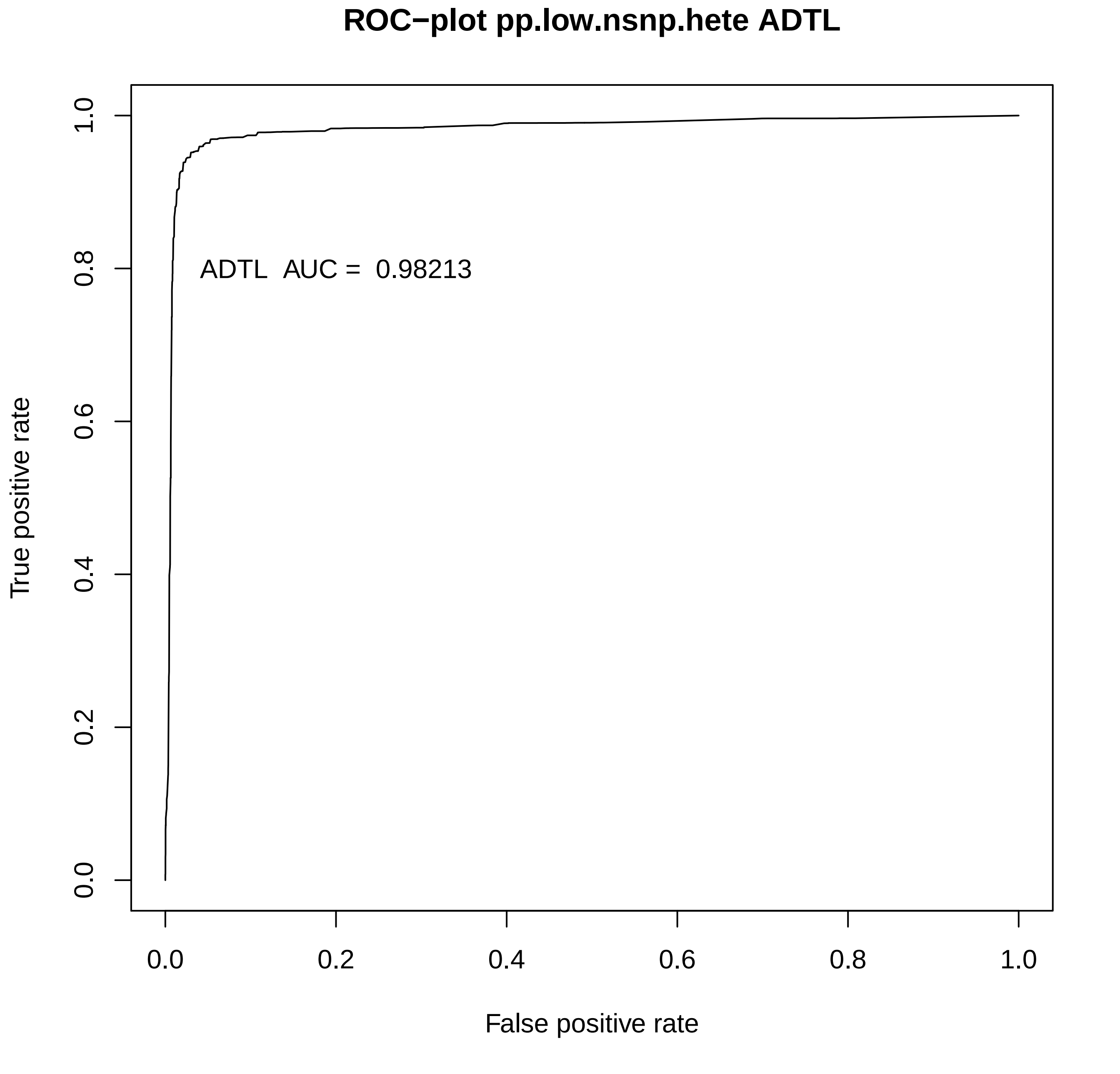
ROC−plot pp.low.nsnp.hete ADTL
ADTL AUC = 0.98213
True positive rate
False positive rate
0.0
0.2
0.4
0.6
0.8
1.0

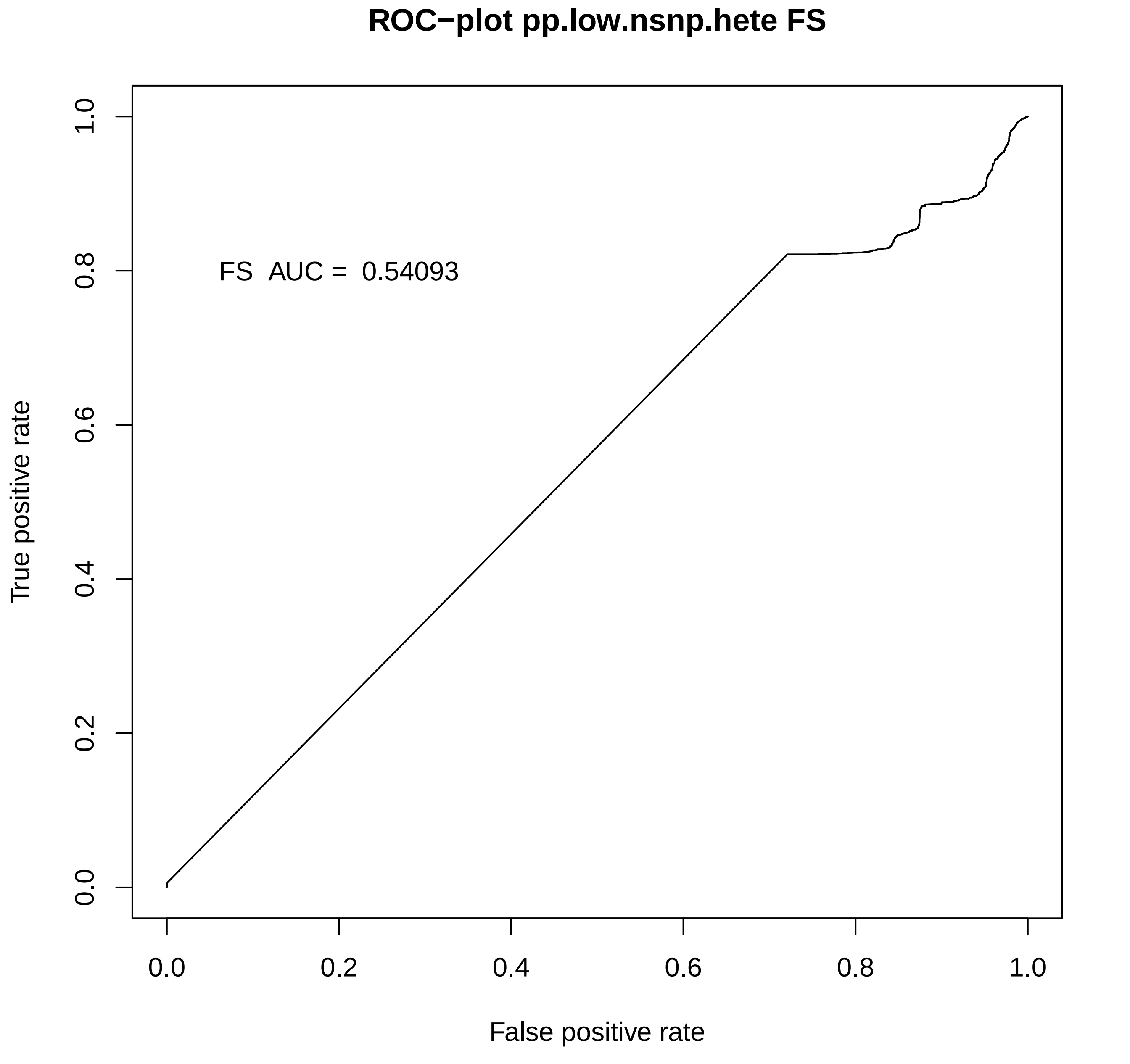
ROC−plot pp.low.nsnp.hete FS
FS AUC = 0.54093
True positive rate
False positive rate
0.0
0.2
0.4
0.6
0.8
1.0

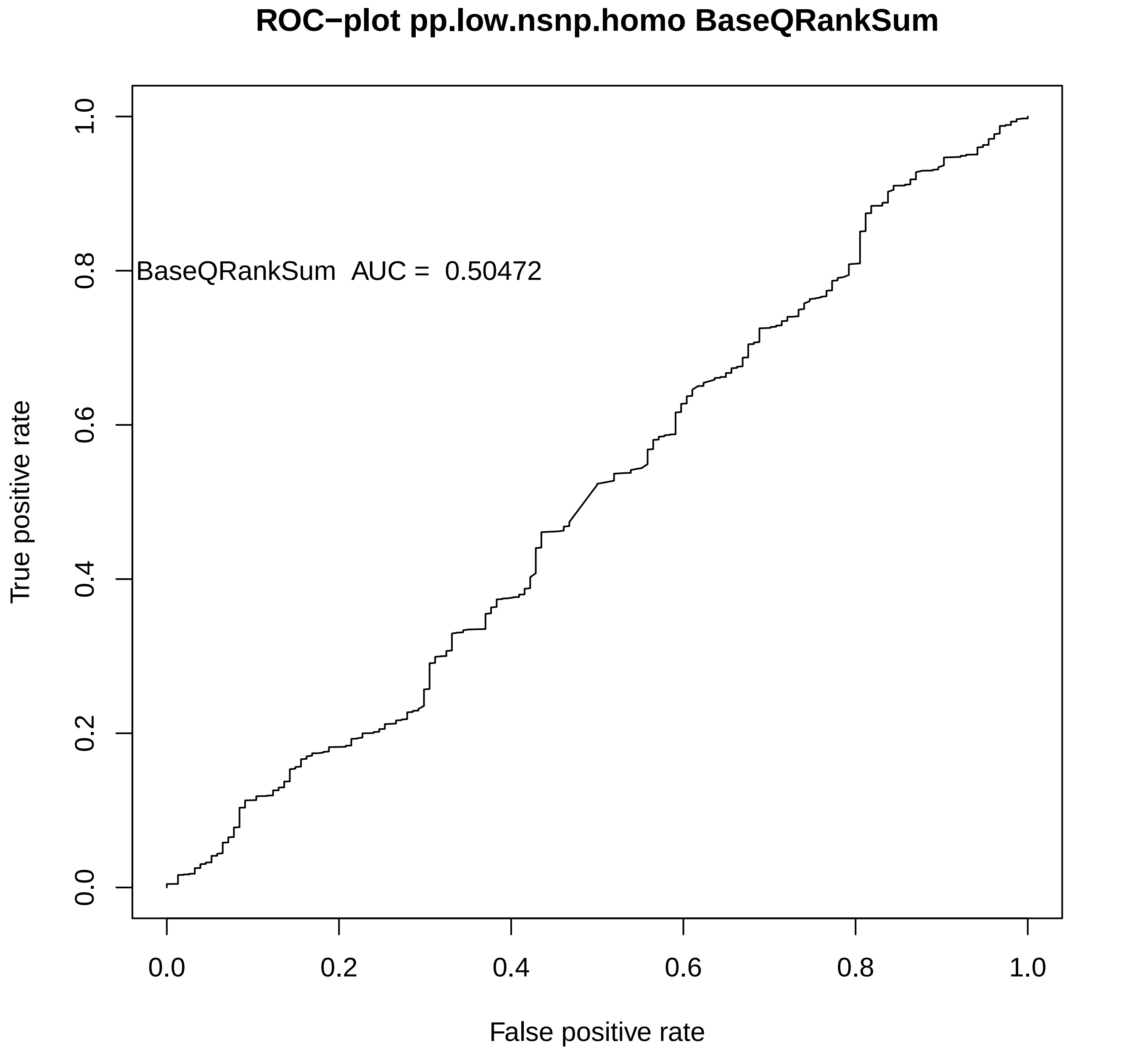

ROC−plot pp.low.nsnp.homo BaseQRankSum
BaseQRankSum AUC = 0.50472
True positive rate
False positive rate
0.0
0.2
0.4
0.6
0.8
1.0

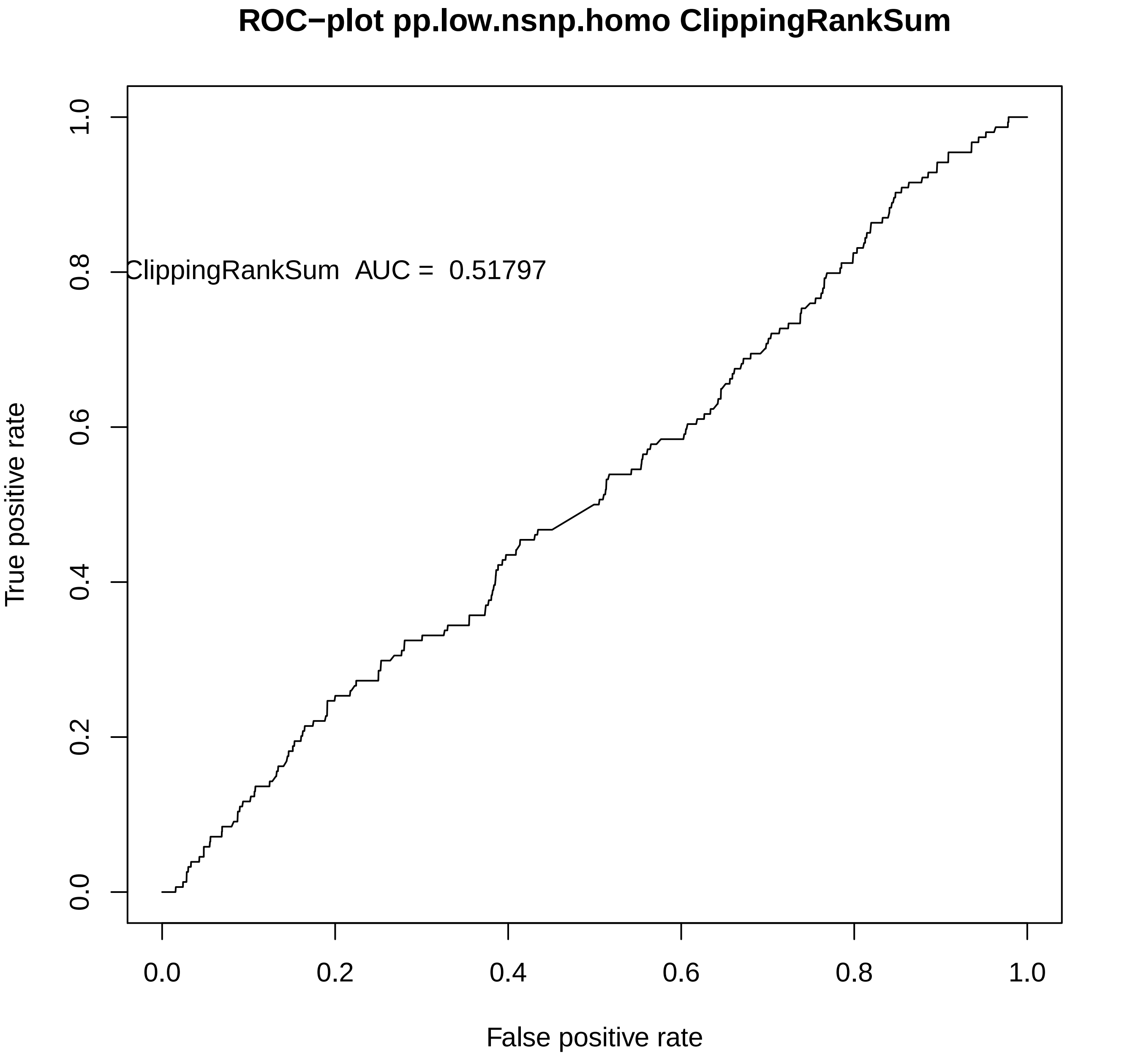
ROC−plot pp.low.nsnp.homo ClippingRankSum
ClippingRankSum AUC = 0.51797
True positive rate
False positive rate
0.0
0.2
0.4
0.6
0.8
1.0

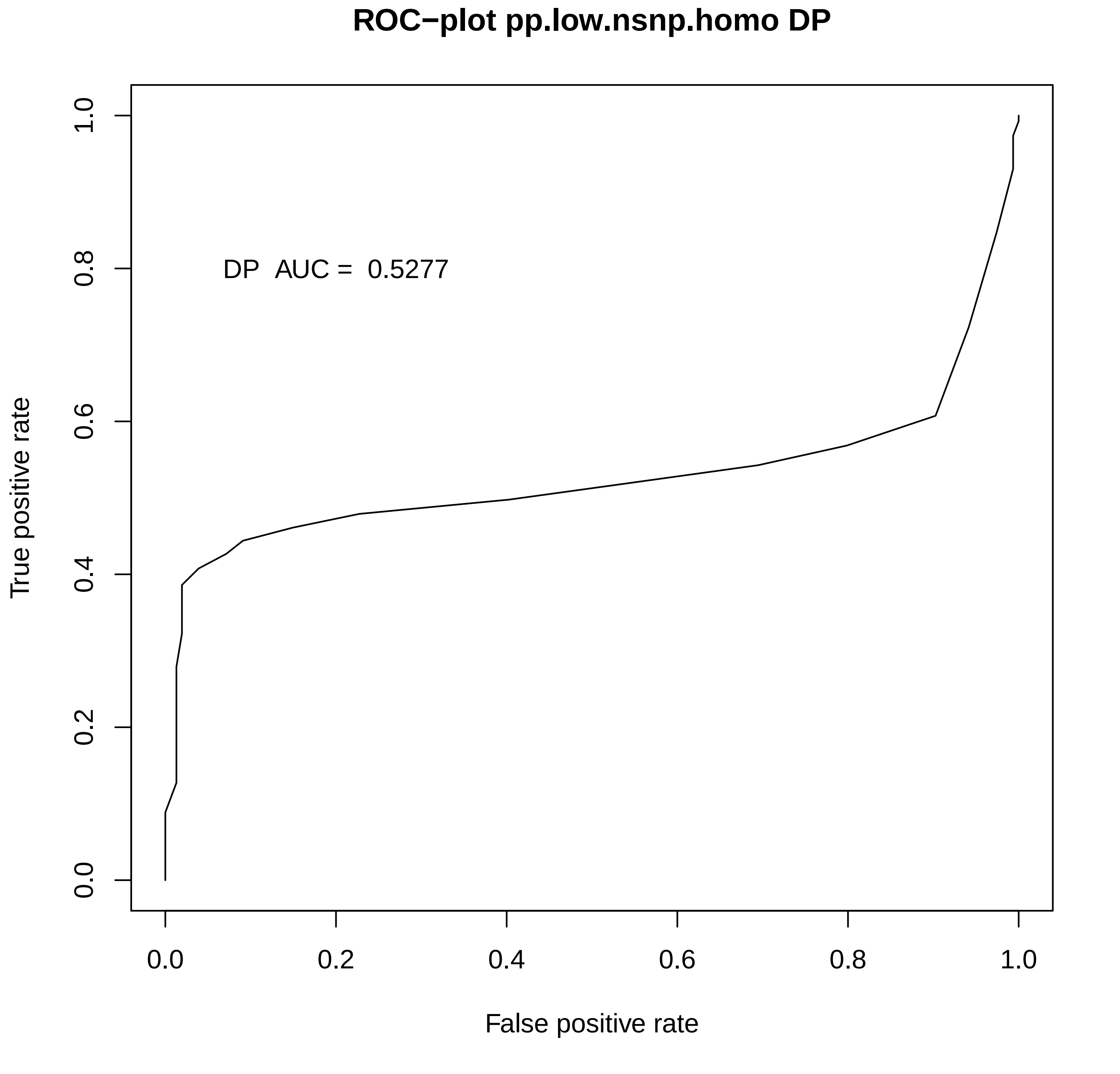
ROC−plot pp.low.nsnp.homo DP
DP AUC = 0.5277
True positive rate
False positive rate
0.0
0.2
0.4
0.6
0.8
1.0

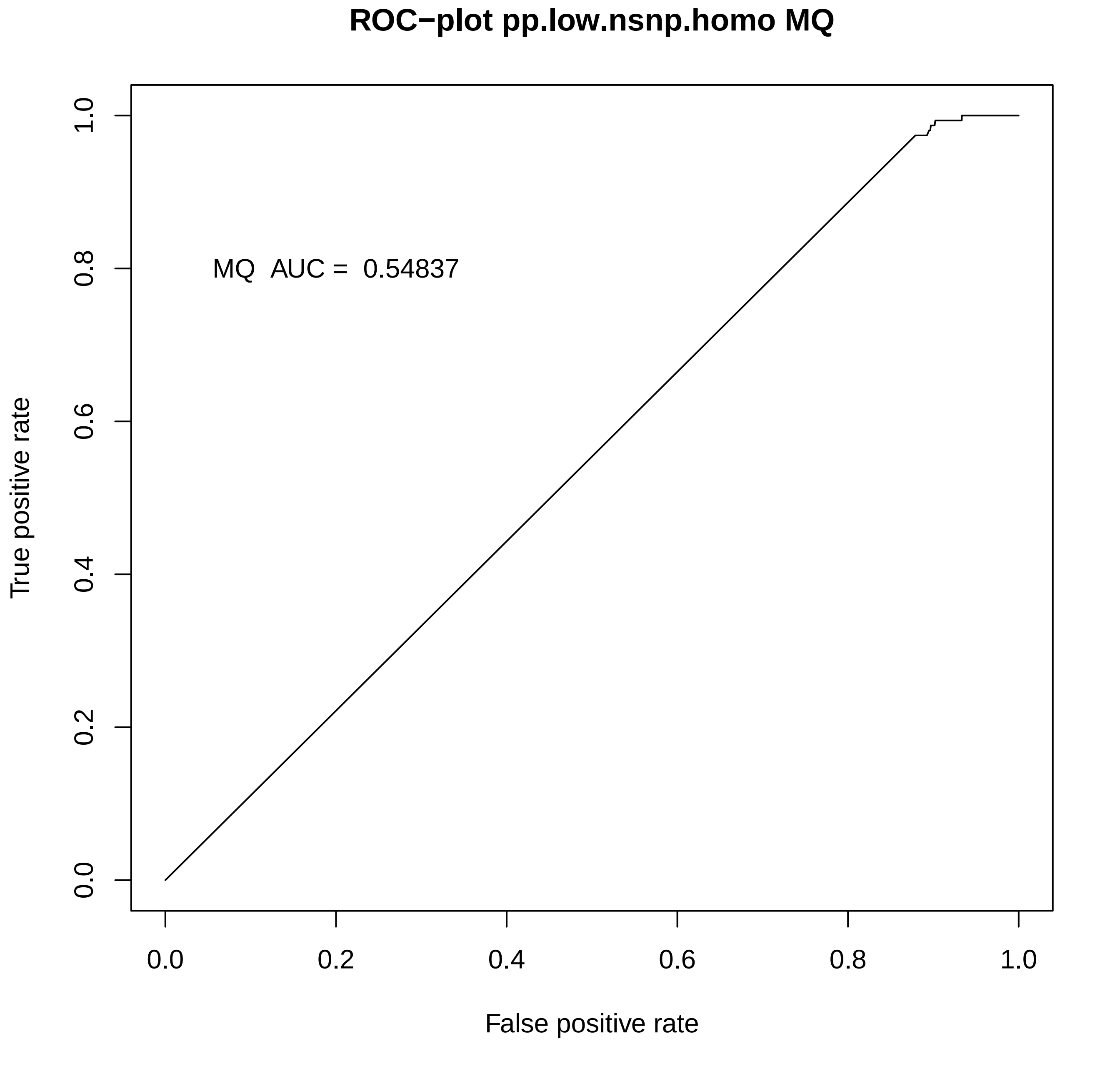
ROC−plot pp.low.nsnp.homo MQ
MQ AUC = 0.54837
True positive rate
False positive rate
0.0
0.2
0.4
0.6
0.8
1.0

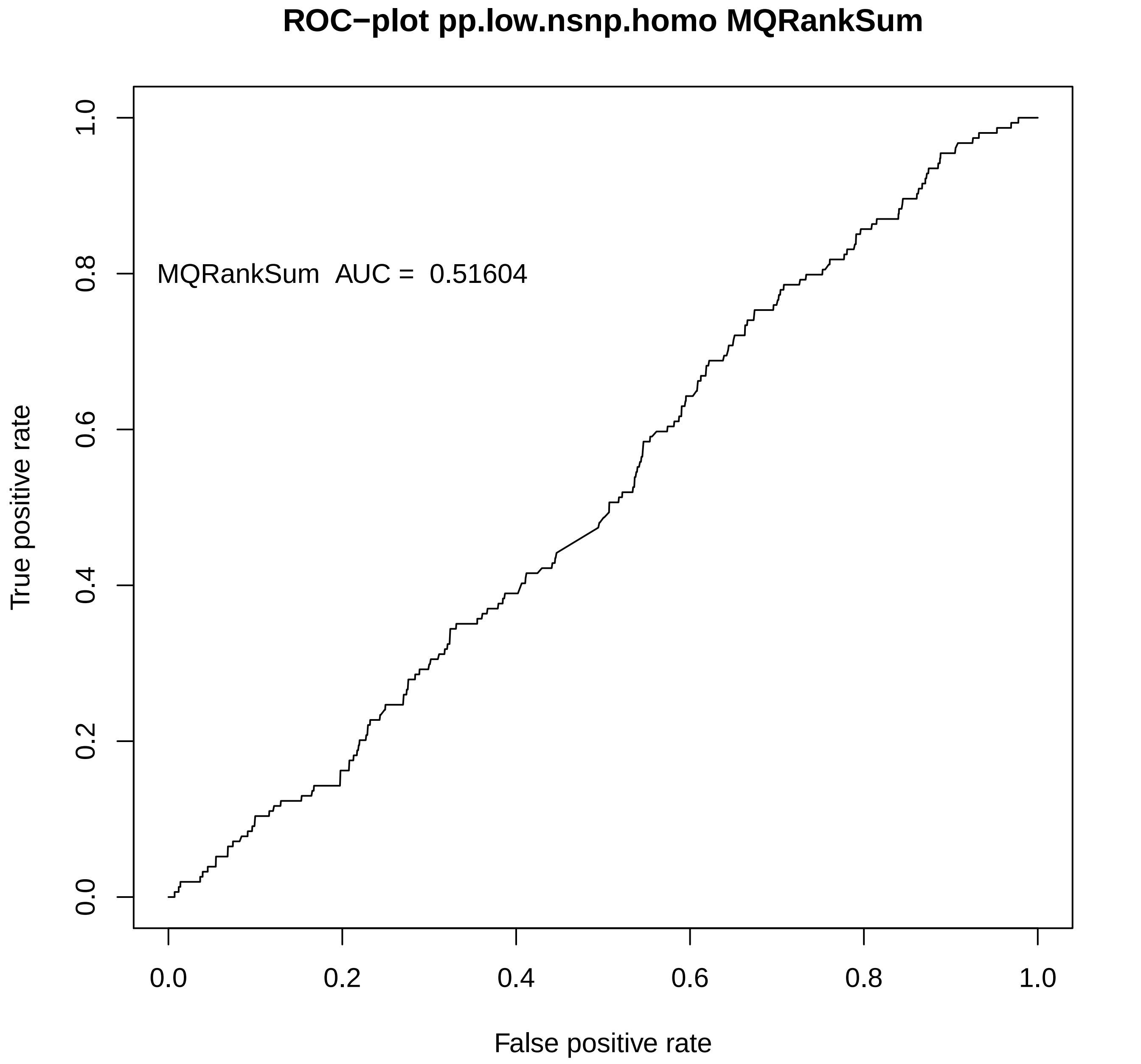
ROC−plot pp.low.nsnp.homo MQRankSum
MQRankSum AUC = 0.51604
True positive rate
False positive rate
0.0
0.2
0.4
0.6
0.8
1.0

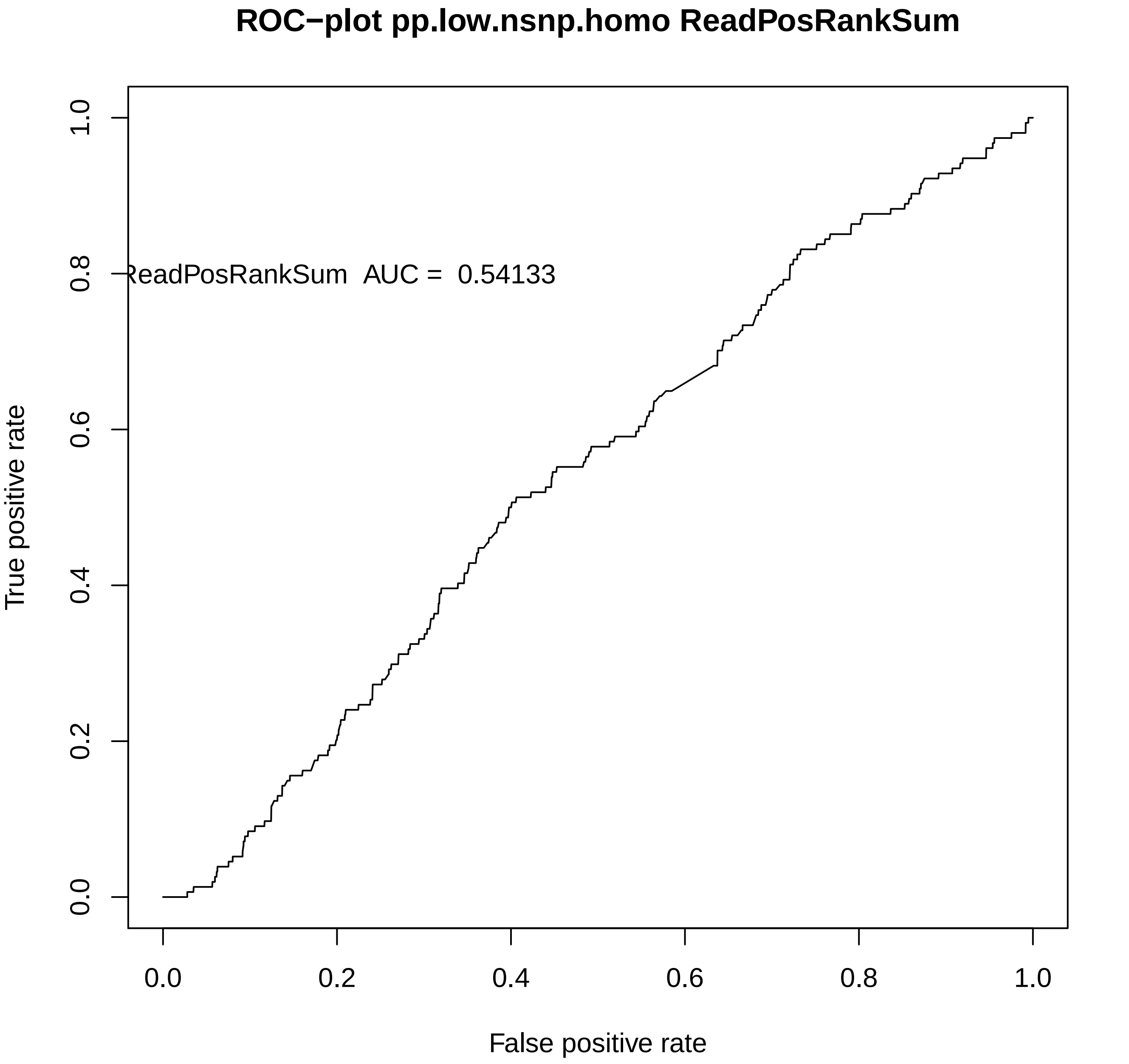
ROC−plot pp.low.nsnp.homo ReadPosRankSum
ReadPosRankSum AUC = 0.54133
True positive rate
False positive rate
0.0
0.2
0.4
0.6
0.8
1.0

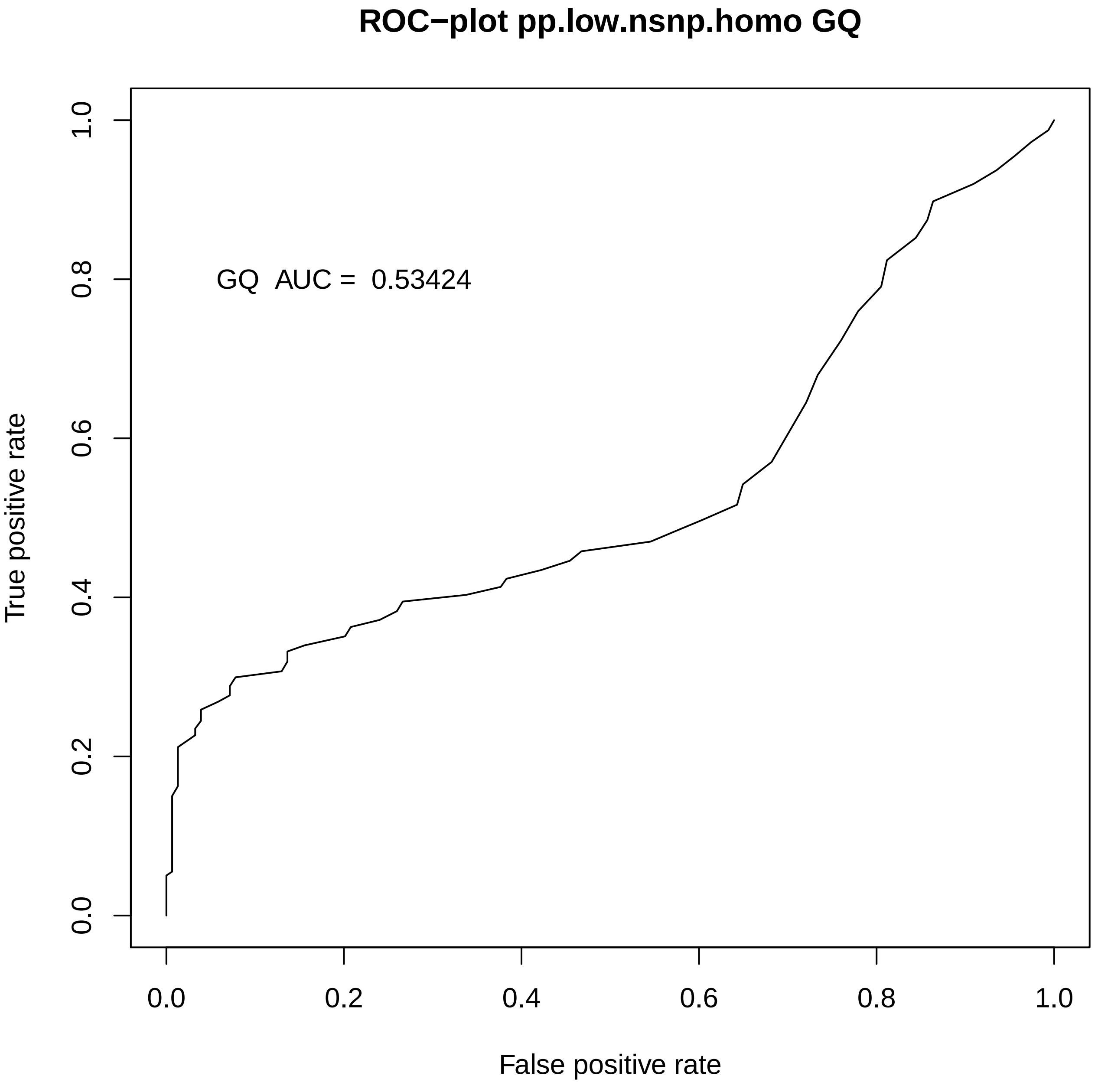
ROC−plot pp.low.nsnp.homo GQ
GQ AUC = 0.53424
True positive rate
False positive rate
0.0
0.2
0.4
0.6
0.8
1.0

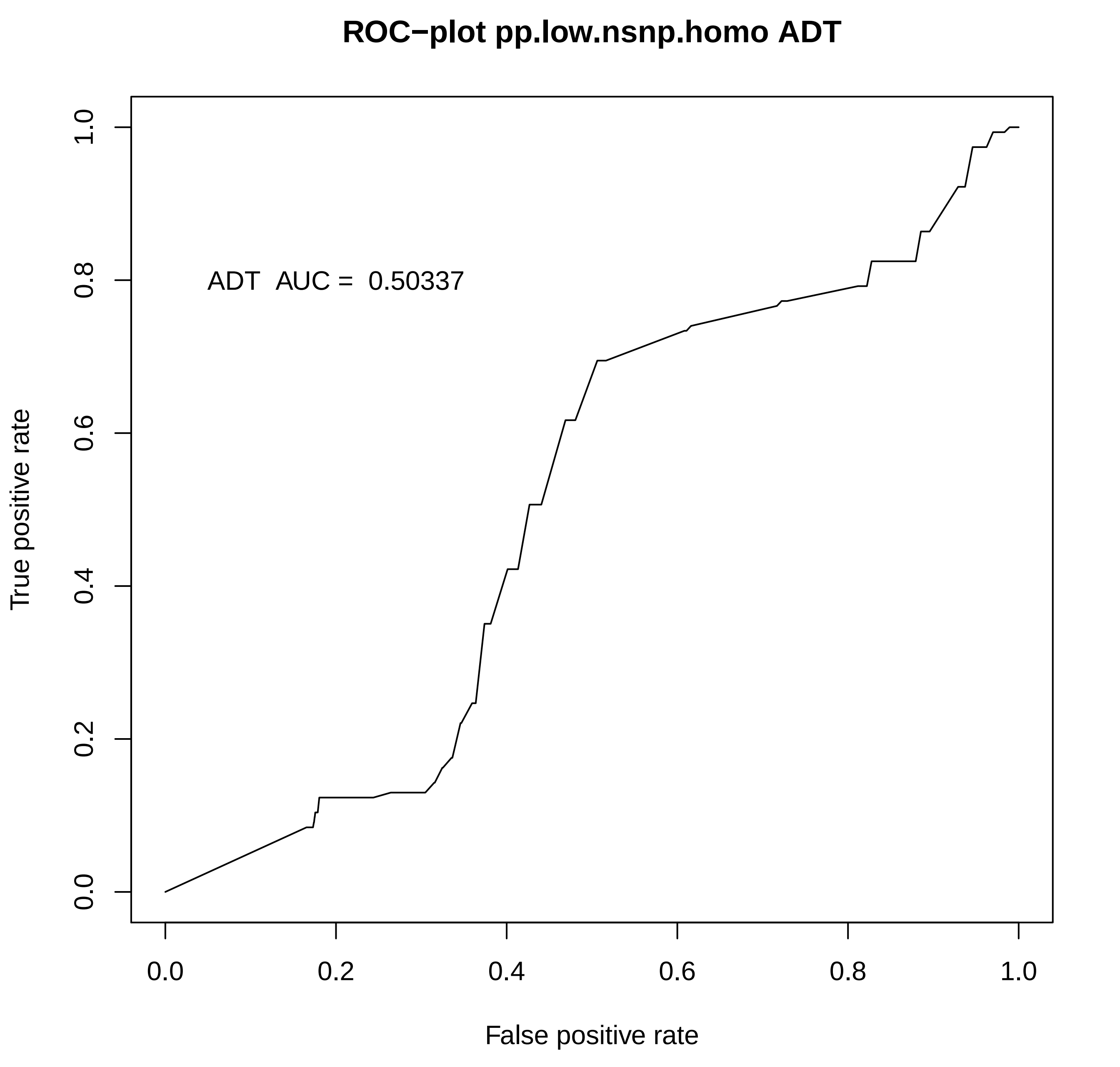
ROC−plot pp.low.nsnp.homo ADT
ADT AUC = 0.50337
True positive rate
False positive rate
0.0
0.2
0.4
0.6
0.8
1.0

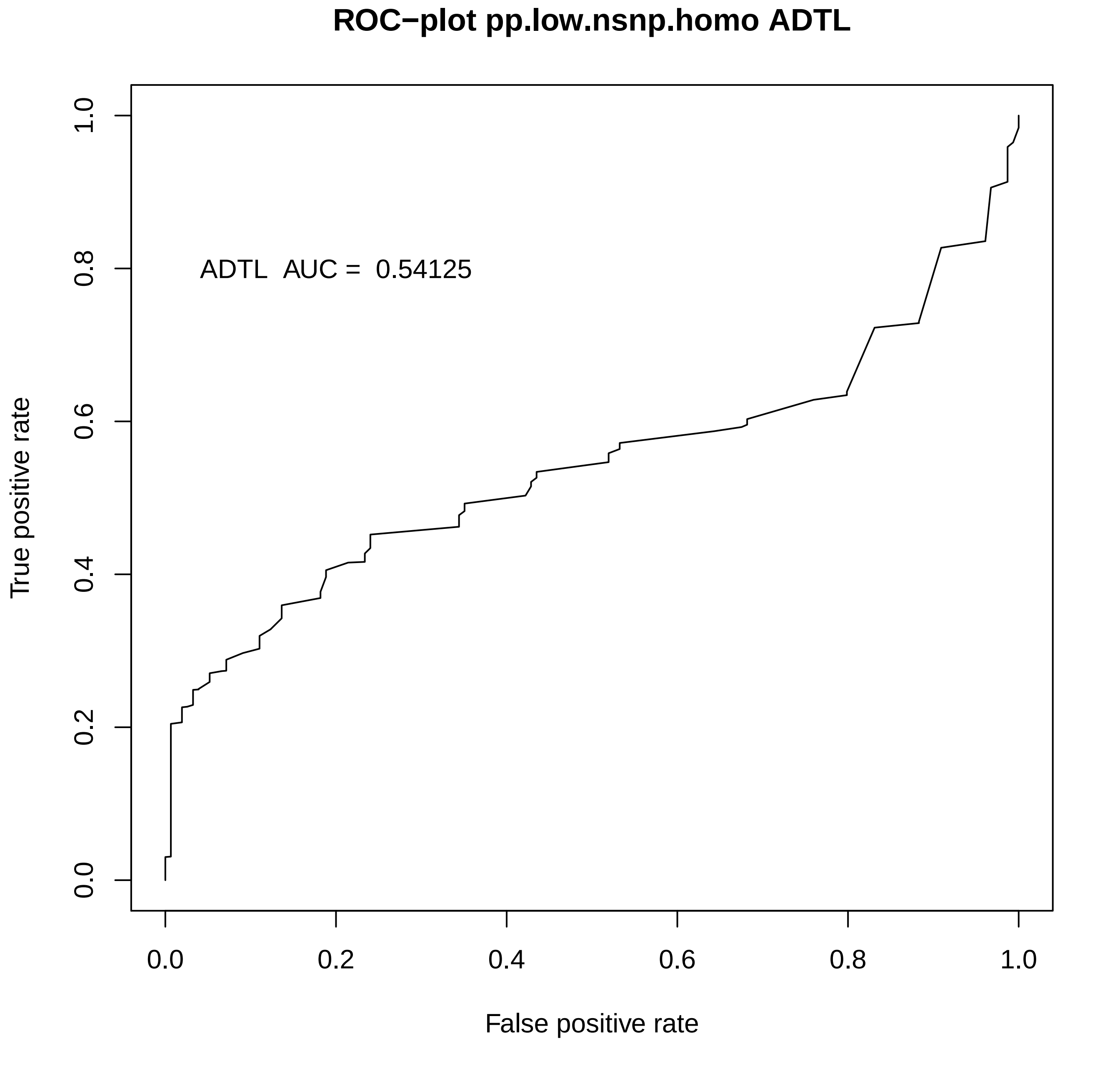

ROC−plot pp.low.nsnp.homo ADTL
ADTL AUC = 0.54125
True positive rate
False positive rate
0.0
0.2
0.4
0.6
0.8
1.0

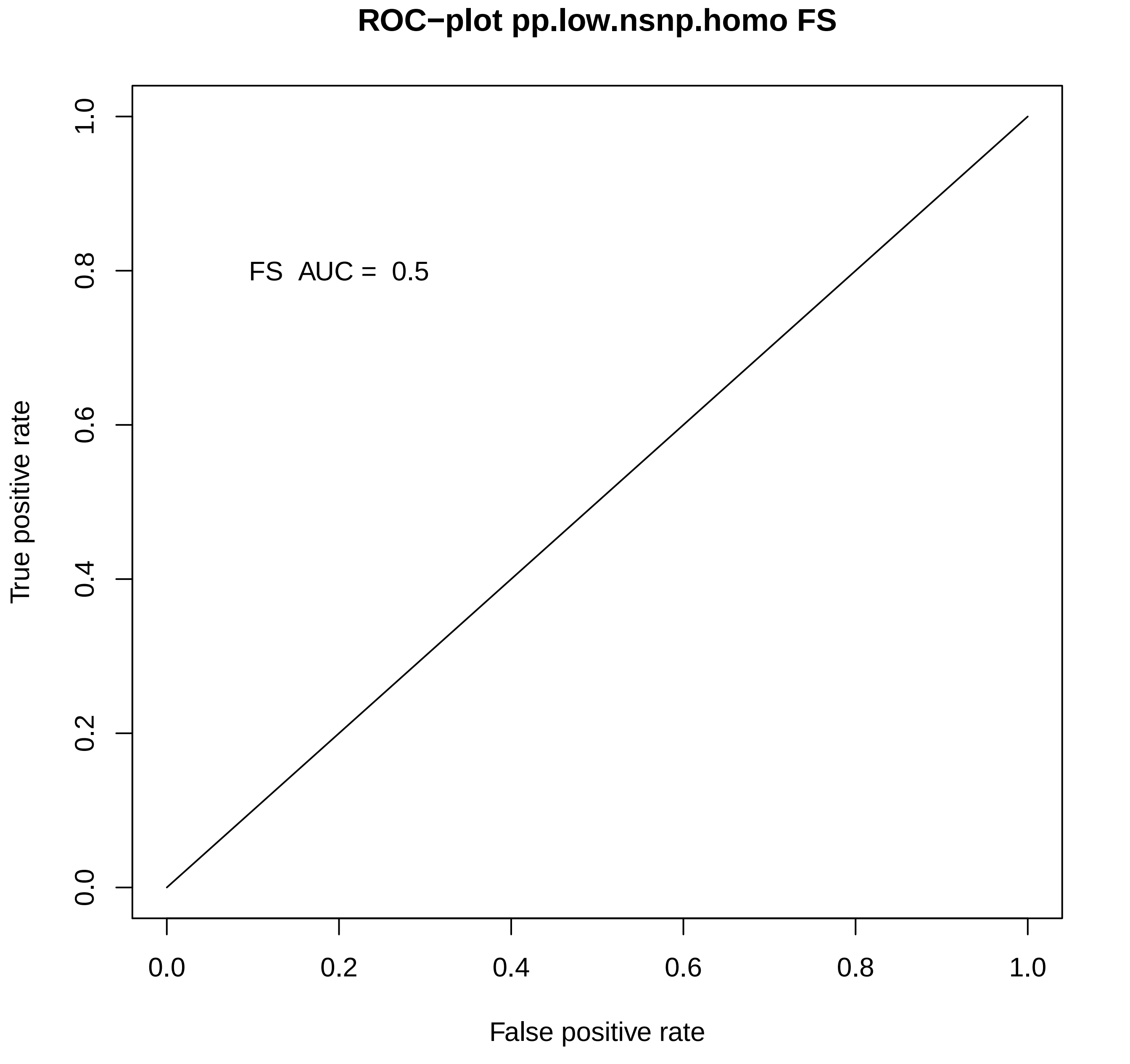

ROC−plot pp.low.nsnp.homo FS
FS AUC = 0.5
True positive rate
False positive rate
0.0
0.2
0.4
0.6
0.8
1.0

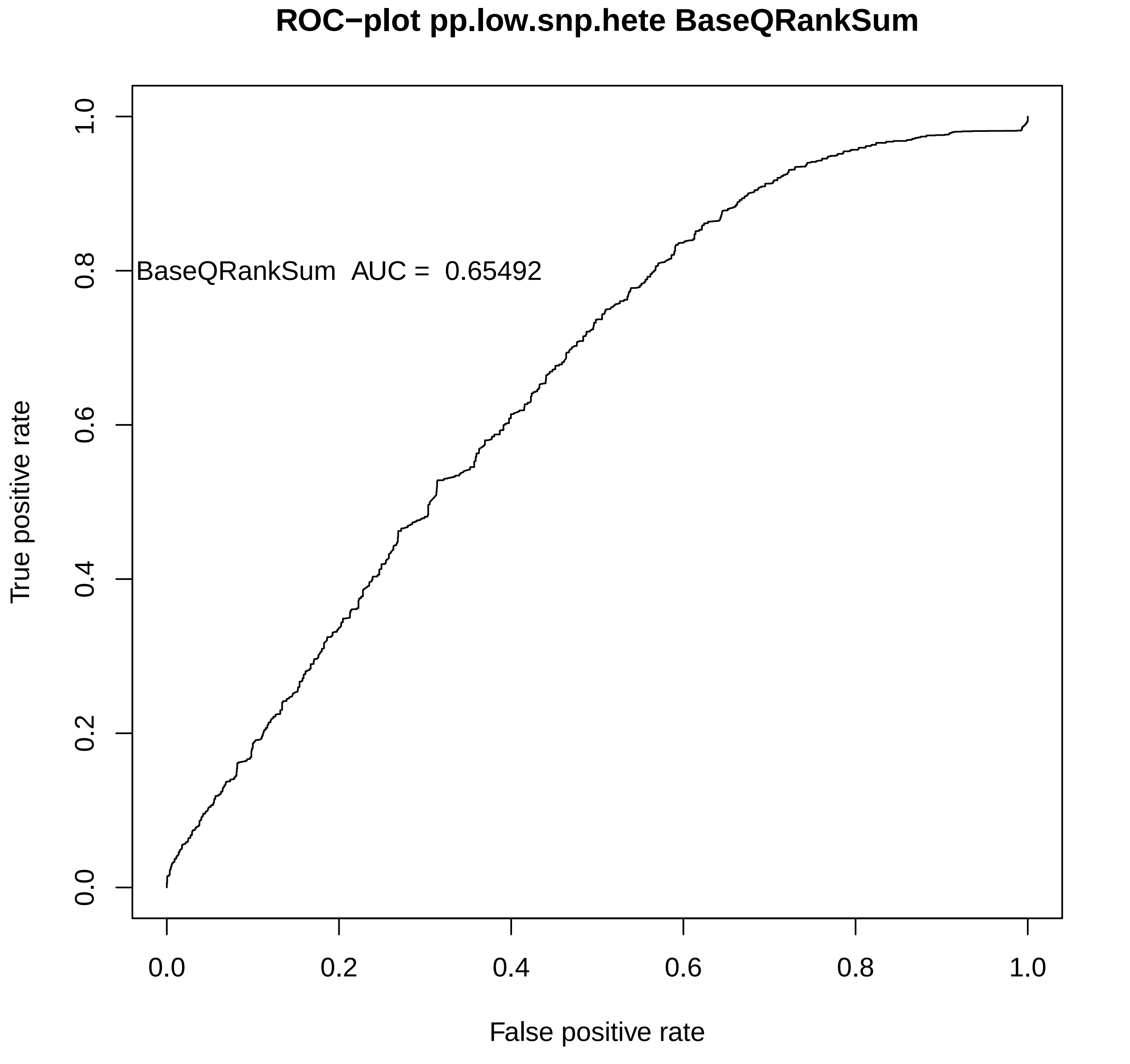
ROC−plot pp.low.snp.hete BaseQRankSum
BaseQRankSum AUC = 0.65492
True positive rate
False positive rate
0.0
0.2
0.4
0.6
0.8
1.0
0.0
0.2
0.4
0.6
0.8
1.0

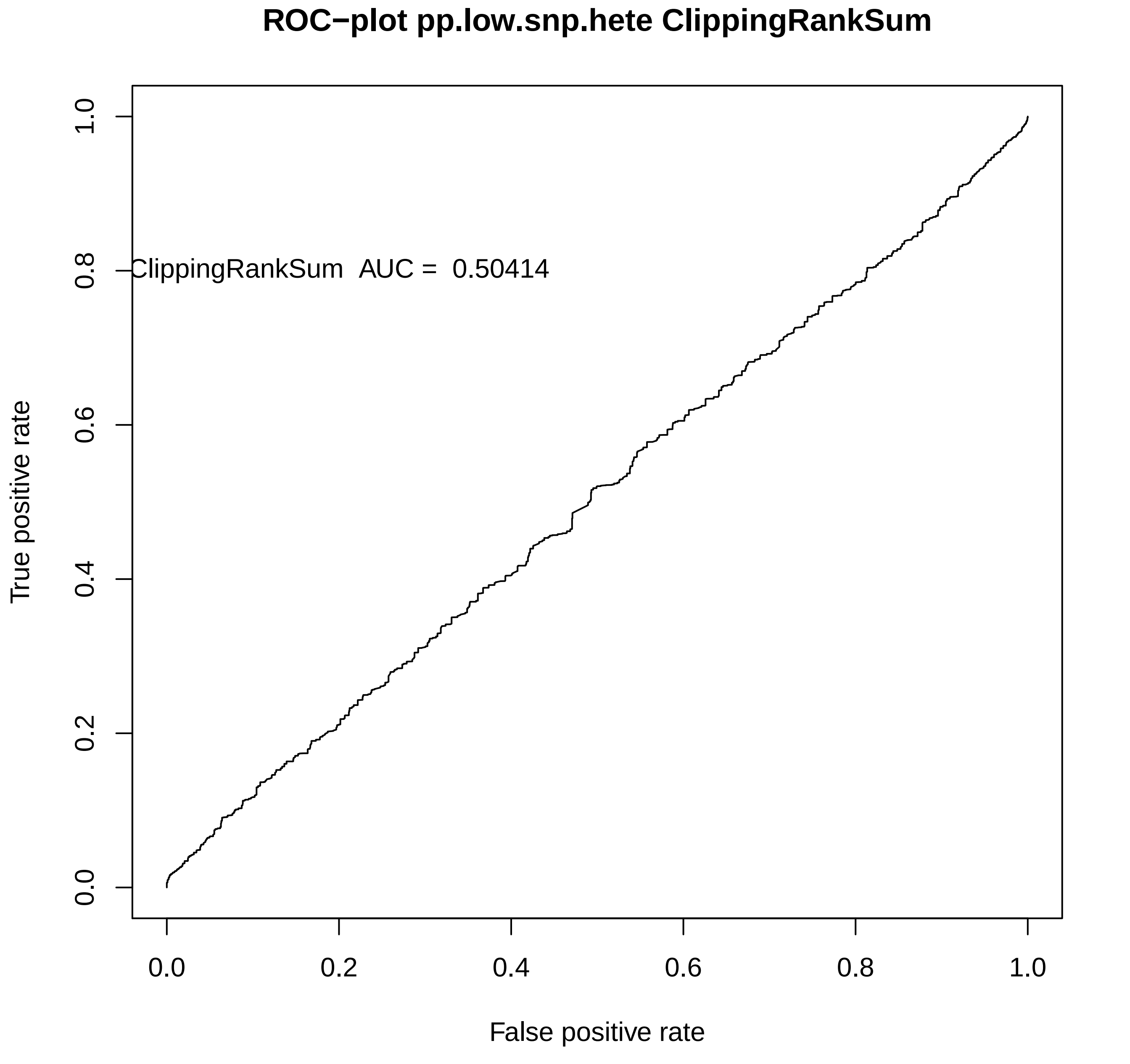
ROC−plot pp.low.snp.hete ClippingRankSum
ClippingRankSum AUC = 0.50414
True positive rate
False positive rate
0.0
0.2
0.4
0.6
0.8
1.0

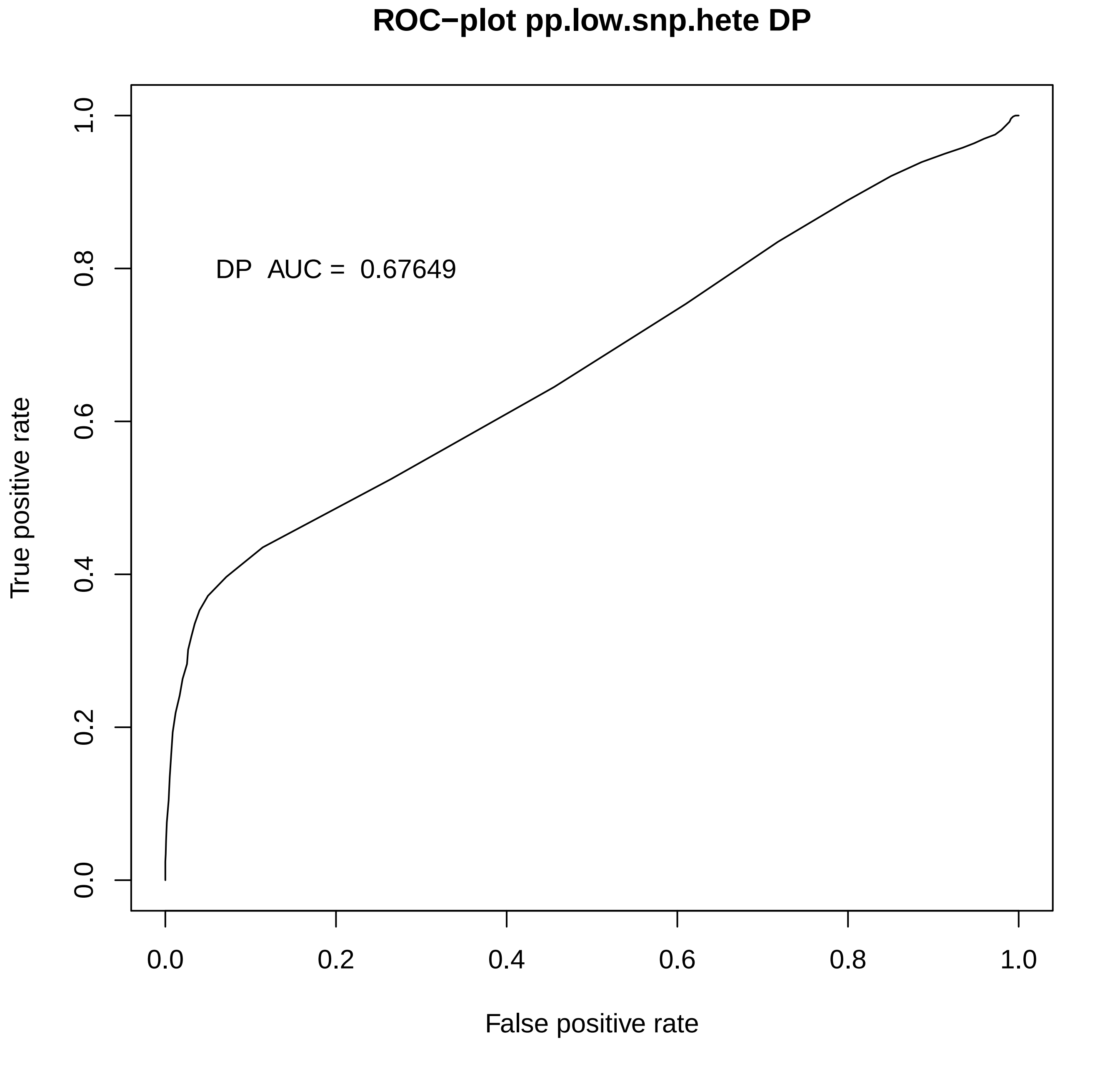

ROC−plot pp.low.snp.hete DP
DP AUC = 0.67649
True positive rate
False positive rate
0.0
0.2
0.4
0.6
0.8
1.0

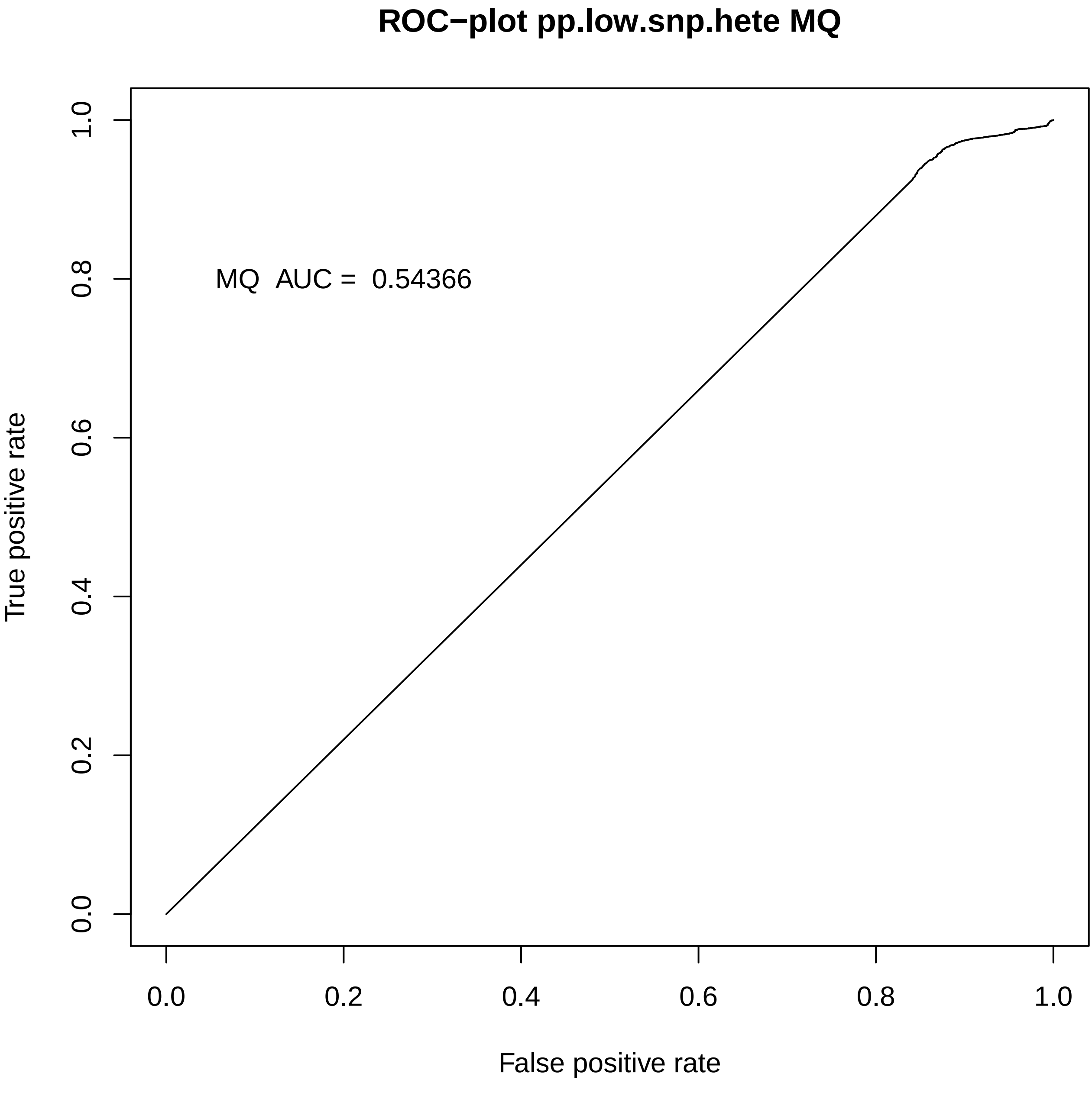

ROC−plot pp.low.snp.hete MQ
MQ AUC = 0.54366
True positive rate
False positive rate
0.0
0.2
0.4
0.6
0.8
1.0

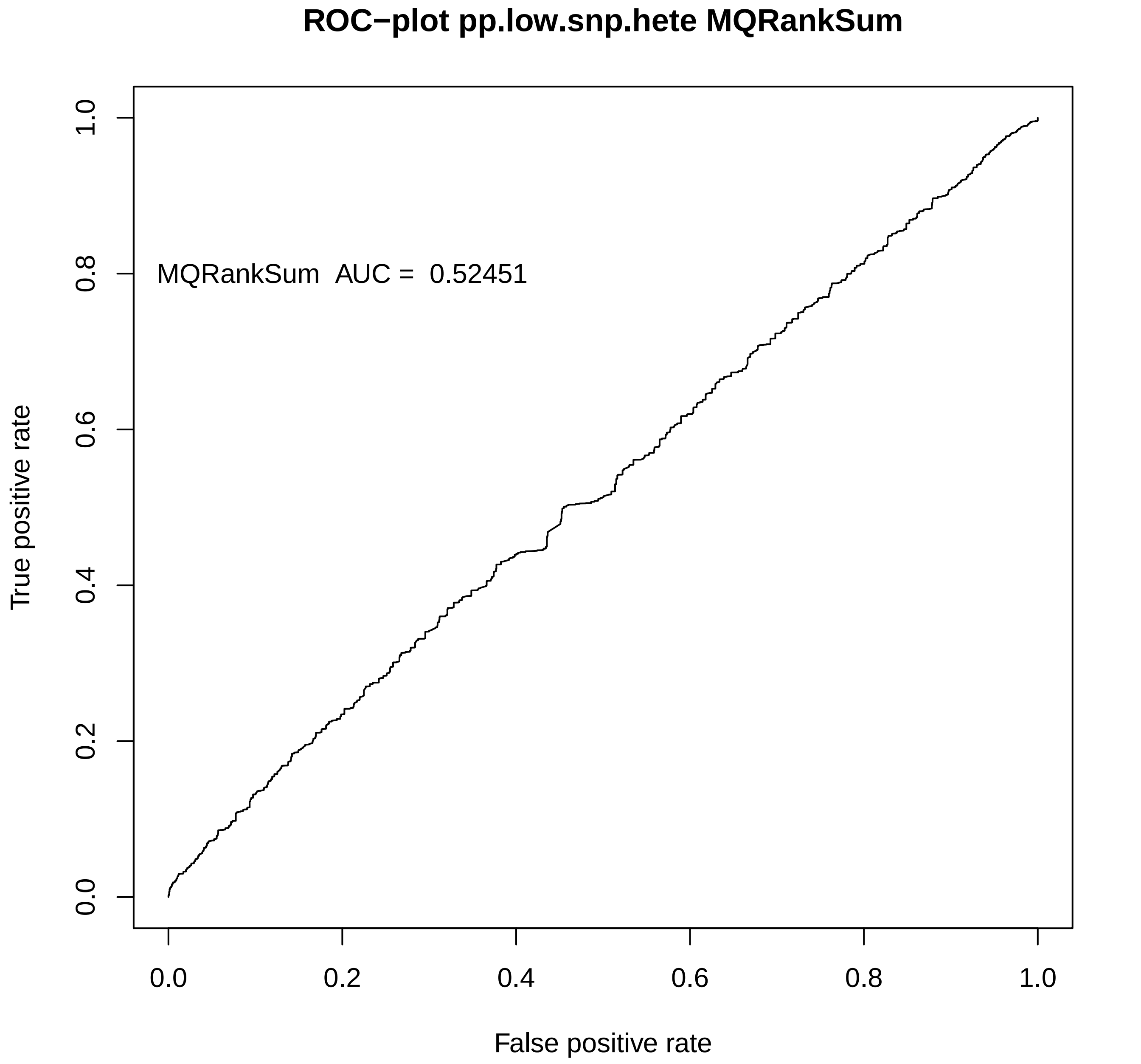
ROC−plot pp.low.snp.hete MQRankSum
MQRankSum AUC = 0.52451
True positive rate
False positive rate
0.0
0.2
0.4
0.6
0.8
1.0

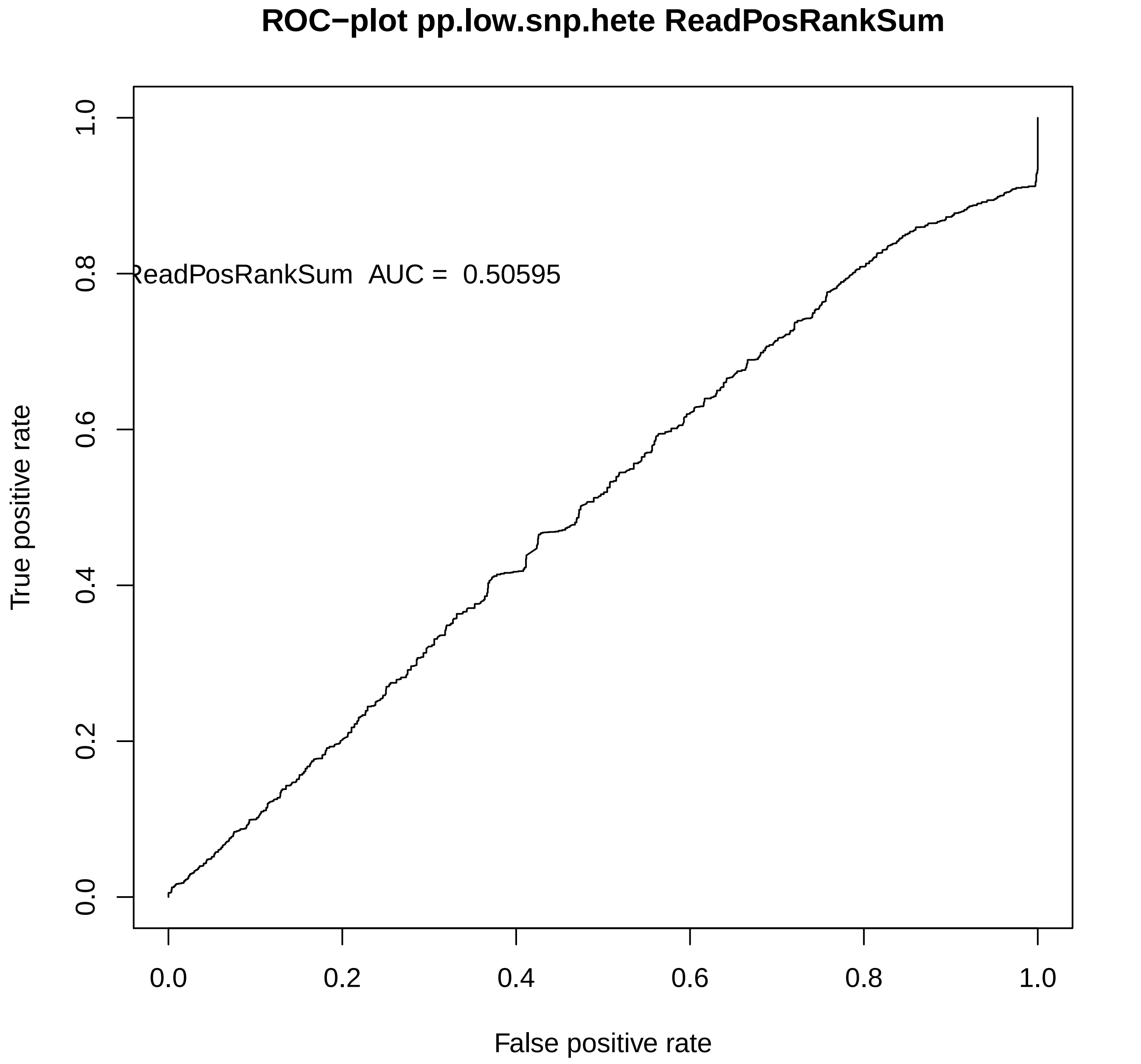
ROC−plot pp.low.snp.hete ReadPosRankSum
ReadPosRankSum AUC = 0.50595
True positive rate
False positive rate
0.0
0.2
0.4
0.6
0.8
1.0

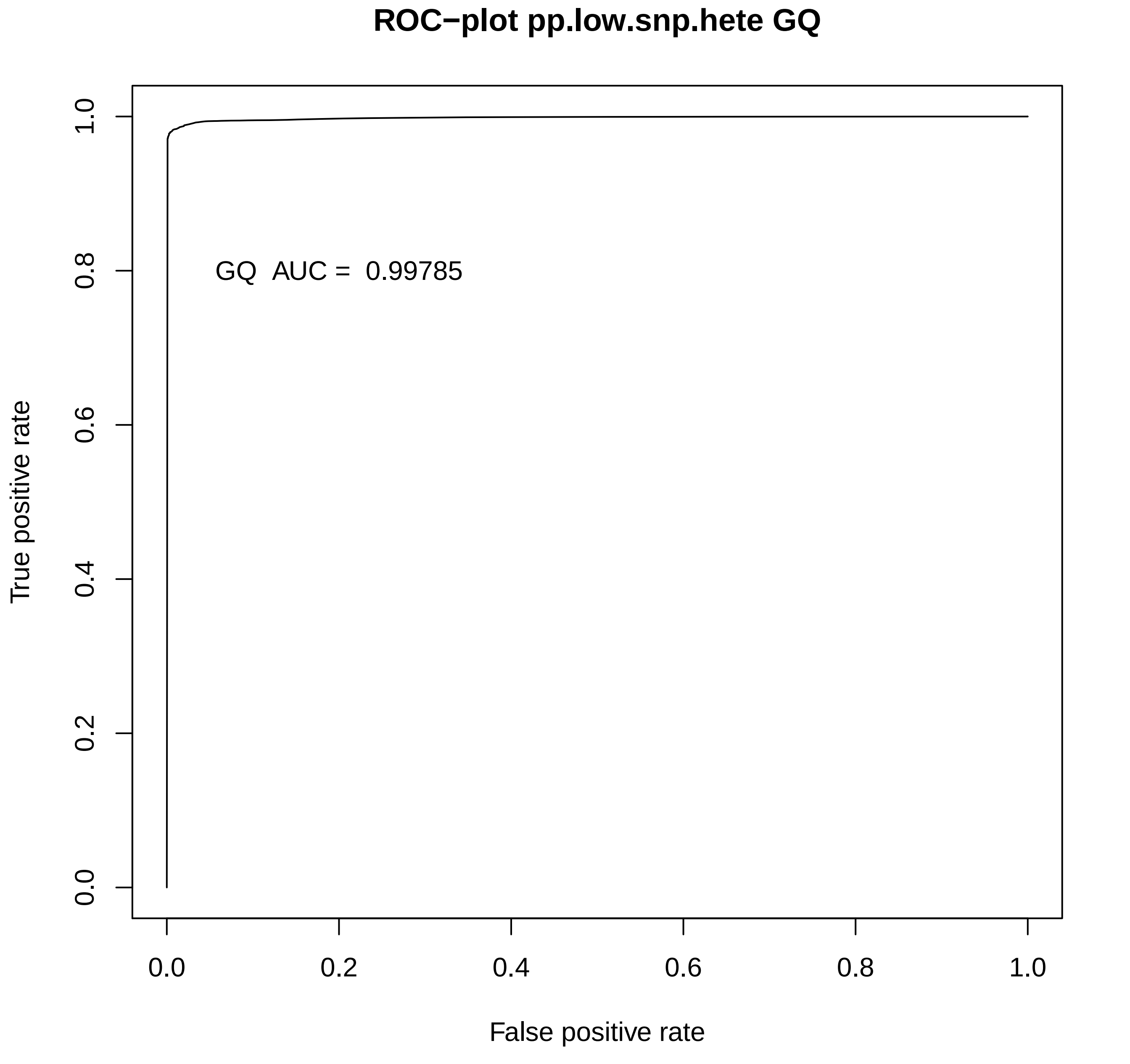
ROC−plot pp.low.snp.hete GQ
GQ AUC = 0.99785
True positive rate
False positive rate
0.0
0.2
0.4
0.6
0.8
1.0

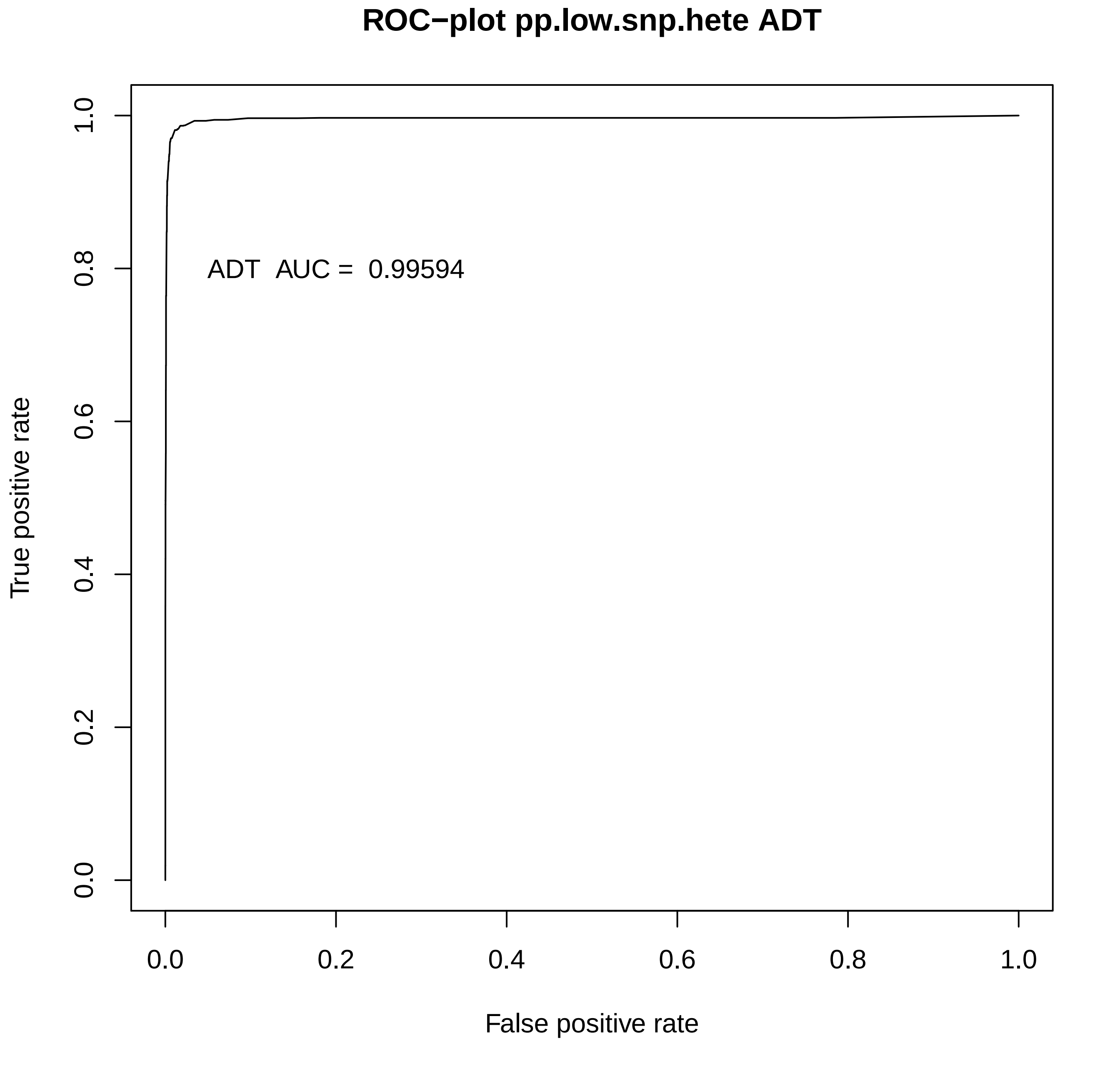
ROC−plot pp.low.snp.hete ADT
ADT AUC = 0.99594
True positive rate
False positive rate
0.0
0.2
0.4
0.6
0.8
1.0

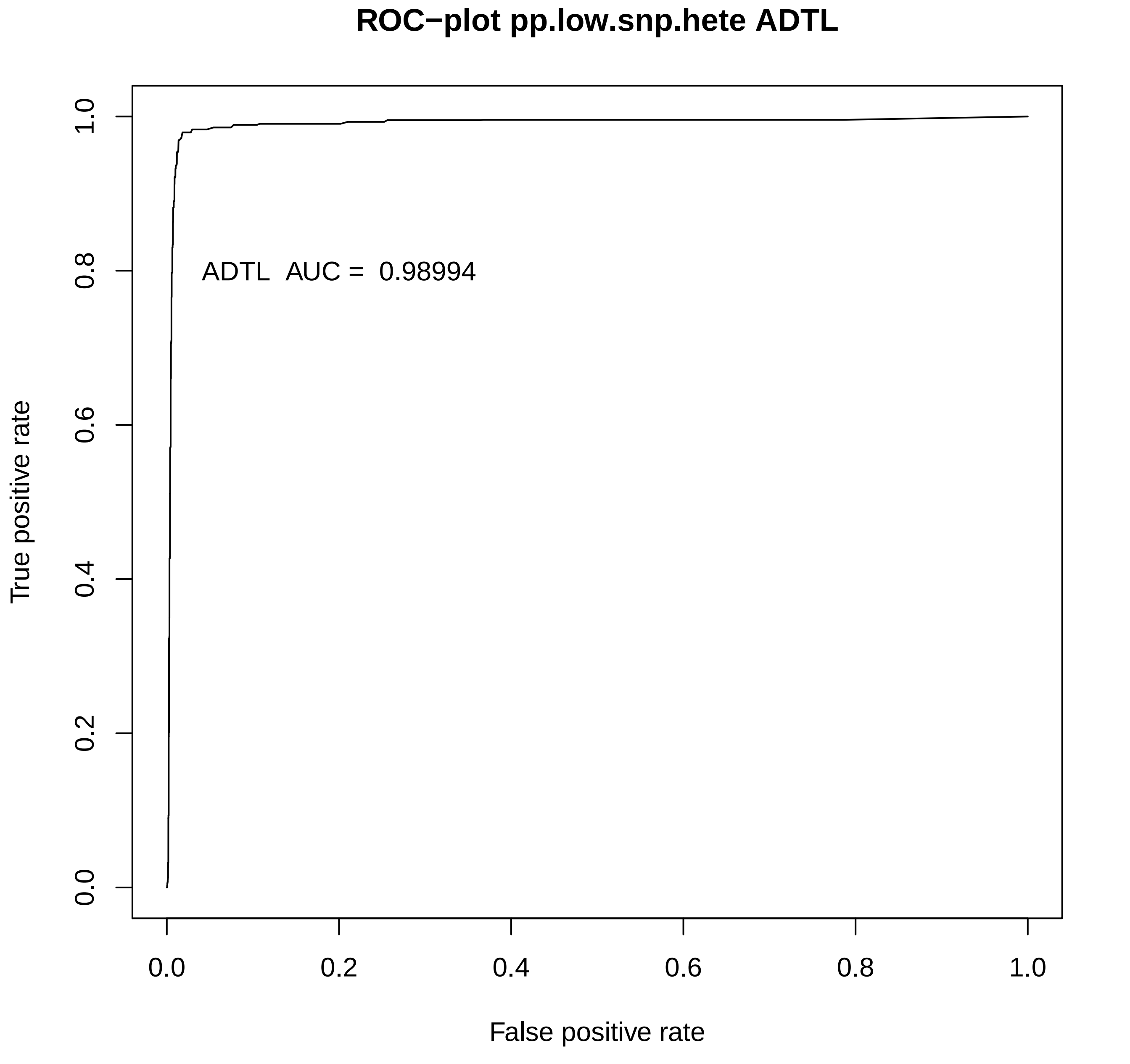
ROC−plot pp.low.snp.hete ADTL
ADTL AUC = 0.98994
True positive rate
False positive rate
0.0
0.2
0.4
0.6
0.8
1.0

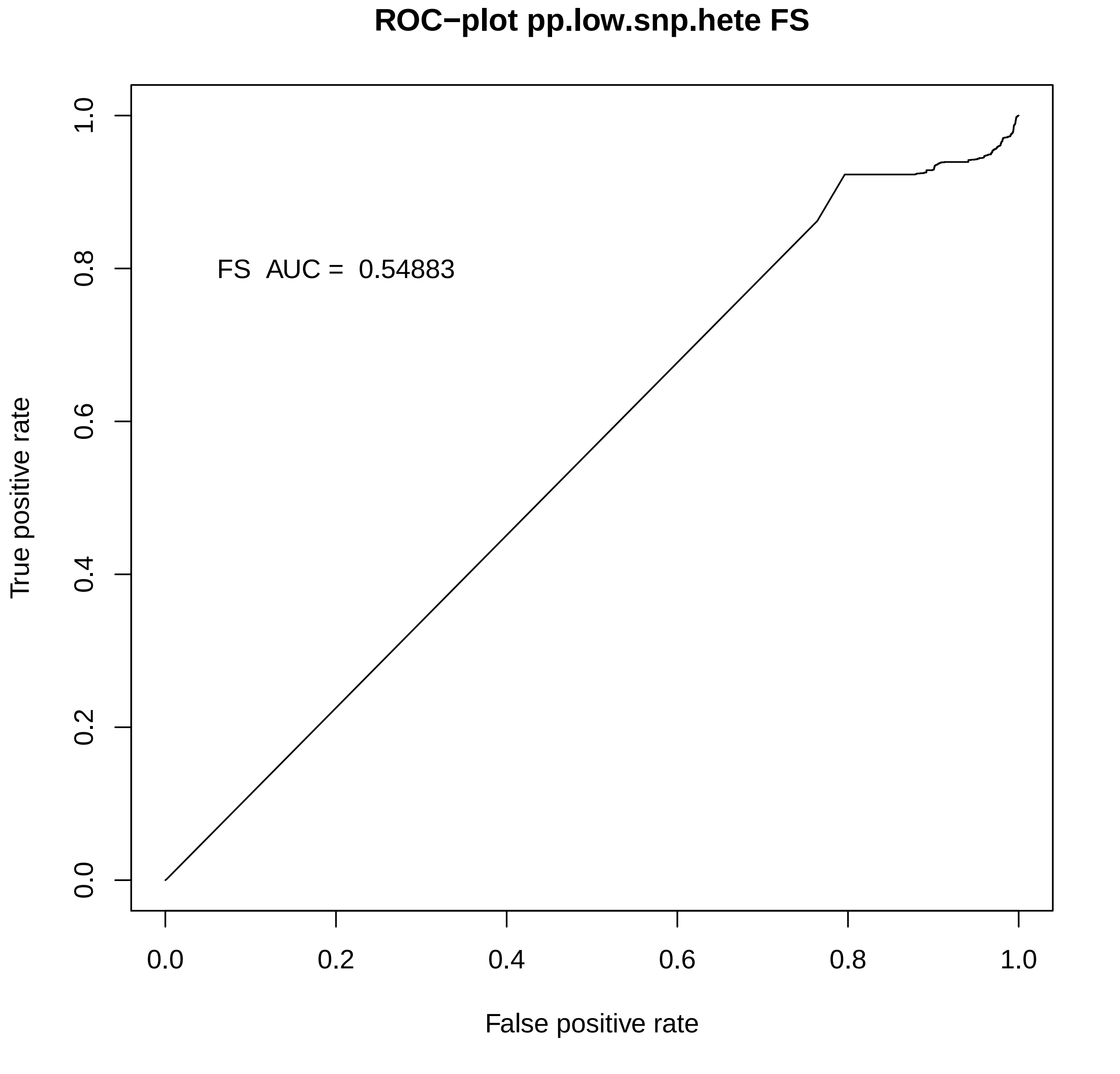
ROC−plot pp.low.snp.hete FS
FS AUC = 0.54883
True positive rate
False positive rate
0.0
0.2
0.4
0.6
0.8
1.0

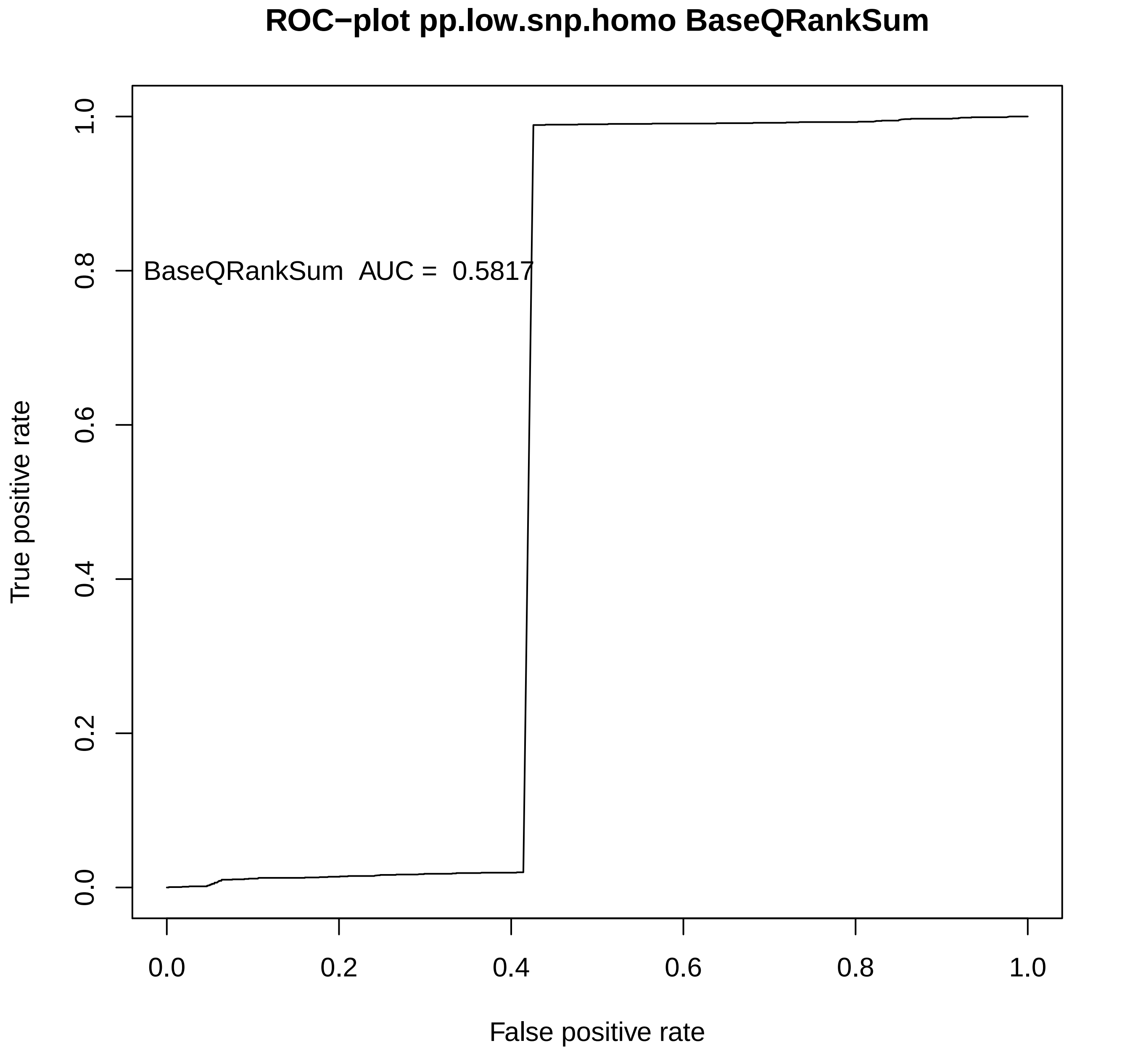

ROC−plot pp.low.snp.homo BaseQRankSum
BaseQRankSum AUC = 0.5817
True positive rate
False positive rate
0.0
0.2
0.4
0.6
0.8
1.0

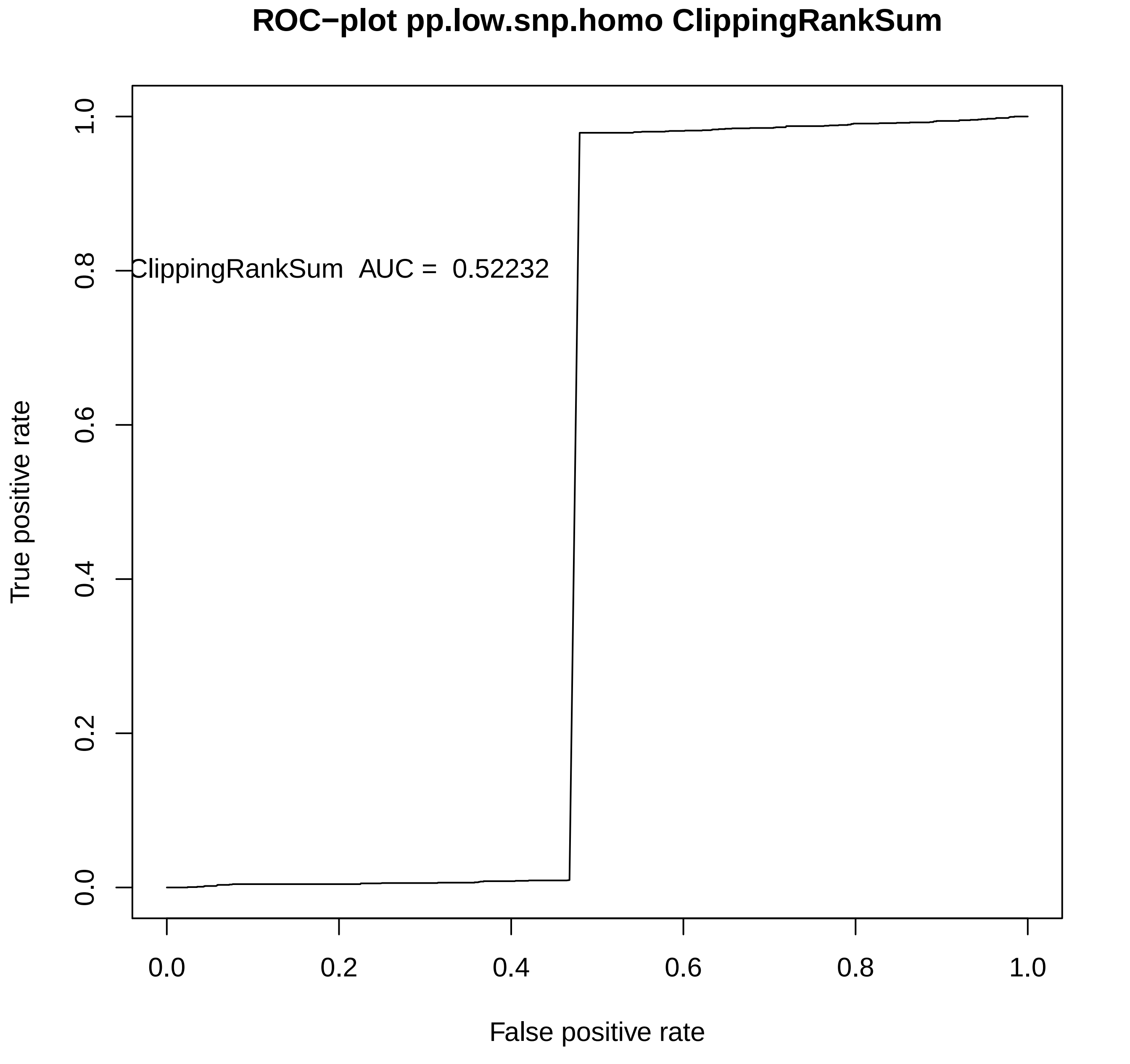
ROC−plot pp.low.snp.homo ClippingRankSum
ClippingRankSum AUC = 0.52232
True positive rate
False positive rate
0.0
0.2
0.4
0.6
0.8
1.0
0.0
0.2
0.4
0.6
0.8
1.0

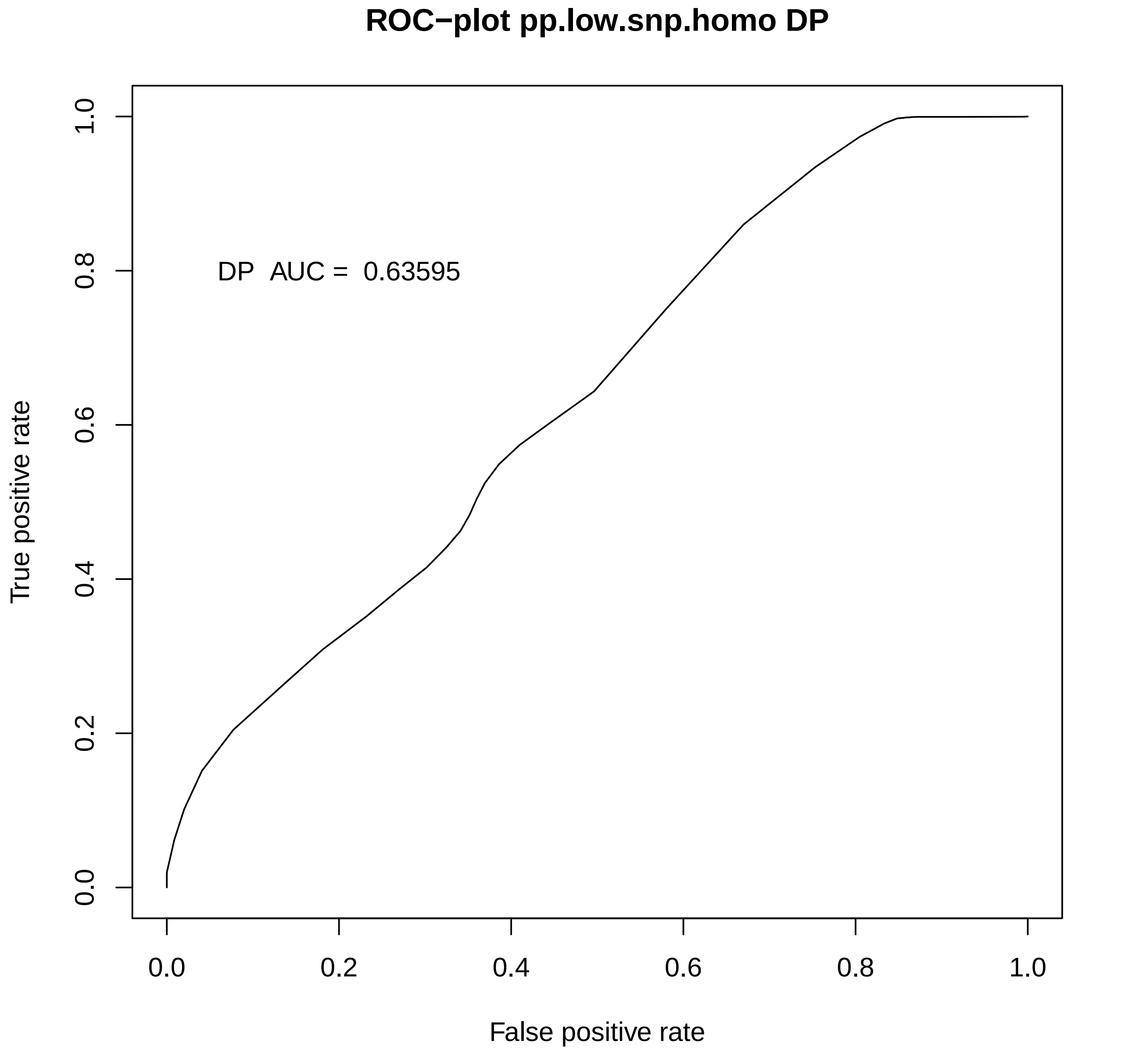
ROC−plot pp.low.snp.homo DP
DP AUC = 0.63595
True positive rate
False positive rate
0.0
0.2
0.4
0.6
0.8
1.0

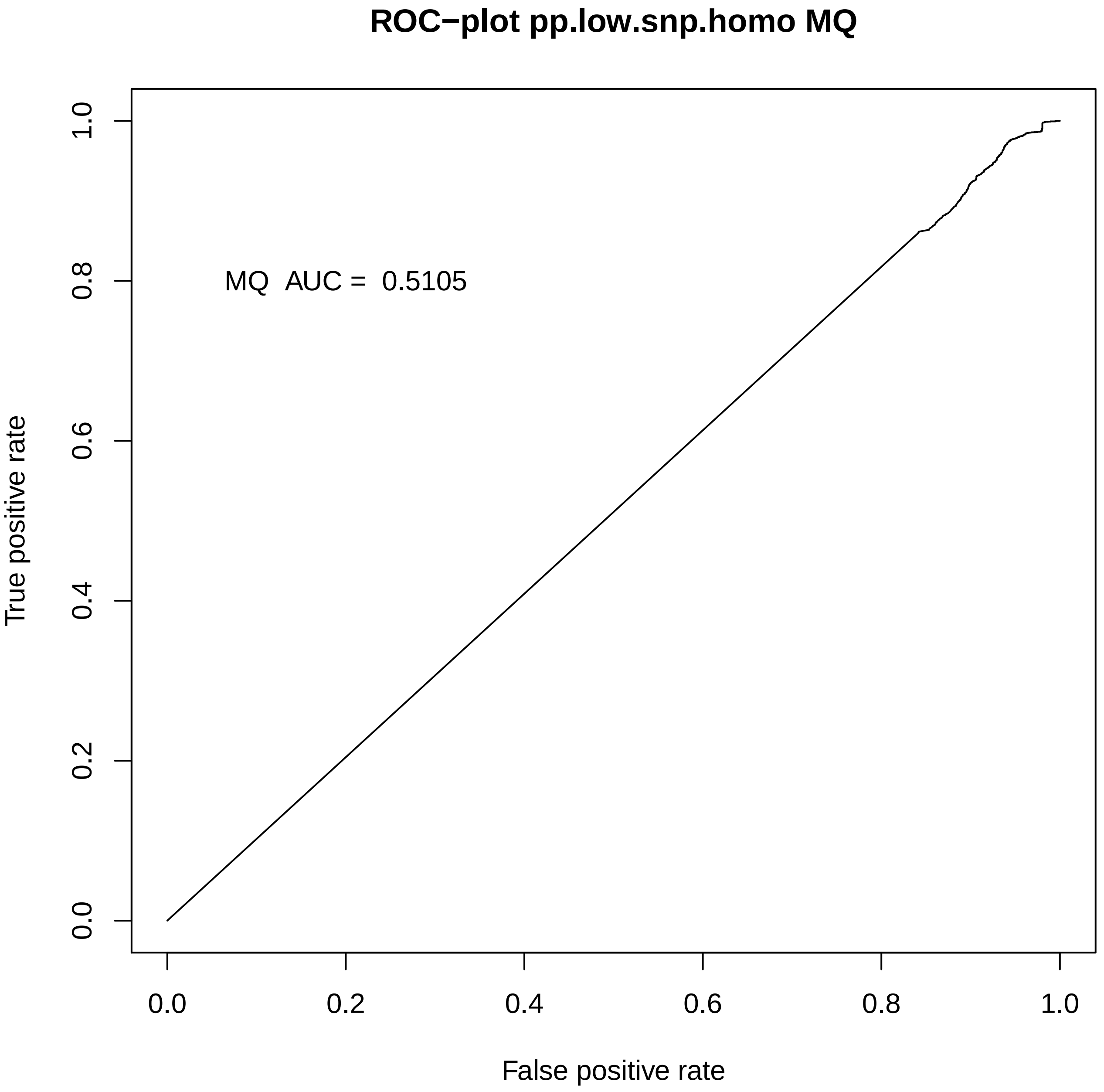
ROC−plot pp.low.snp.homo MQ
MQ AUC = 0.5105
True positive rate
False positive rate
0.0
0.2
0.4
0.6
0.8
1.0

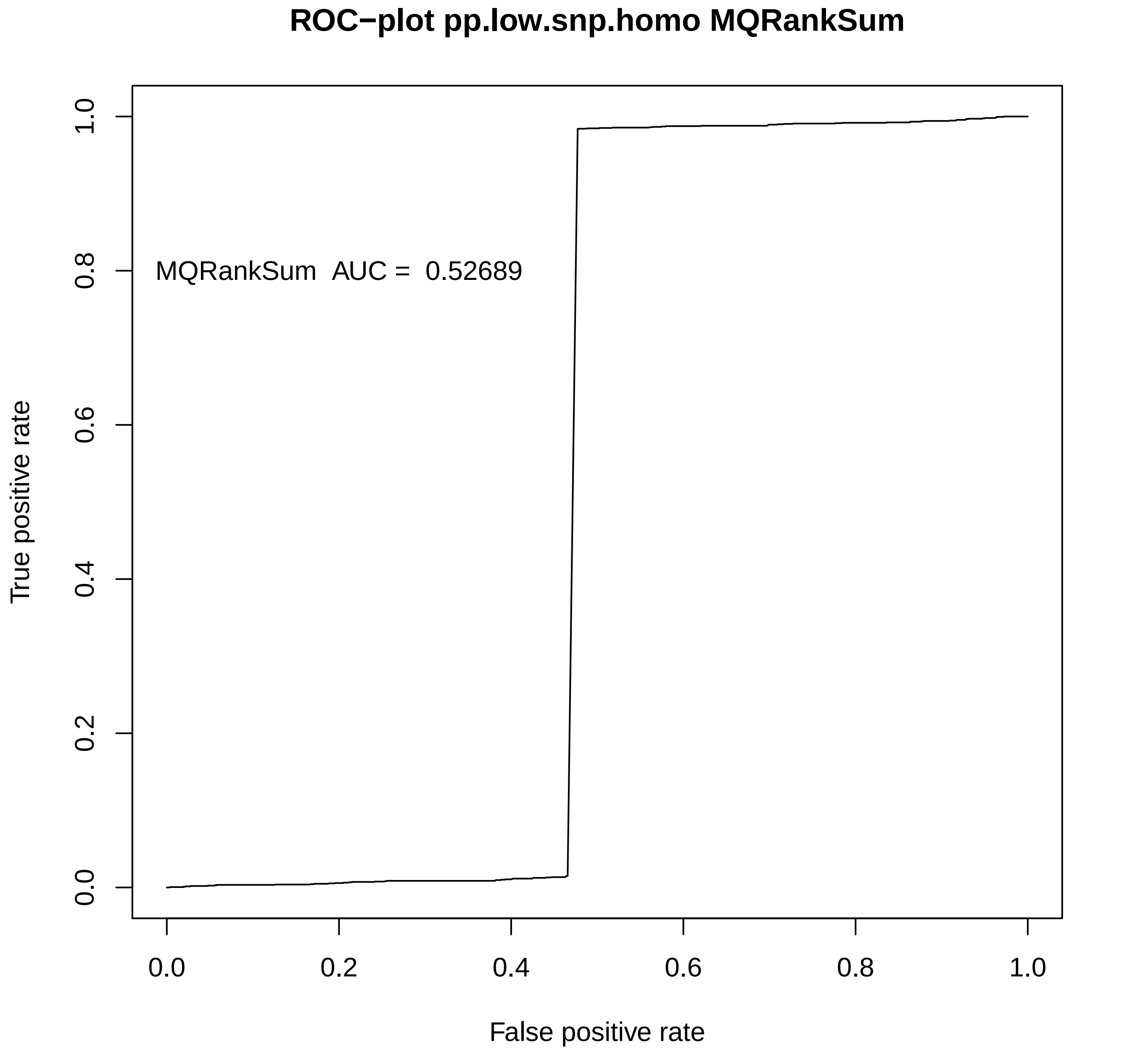

ROC−plot pp.low.snp.homo MQRankSum
MQRankSum AUC = 0.52689
True positive rate
False positive rate
0.0
0.2
0.4
0.6
0.8
1.0

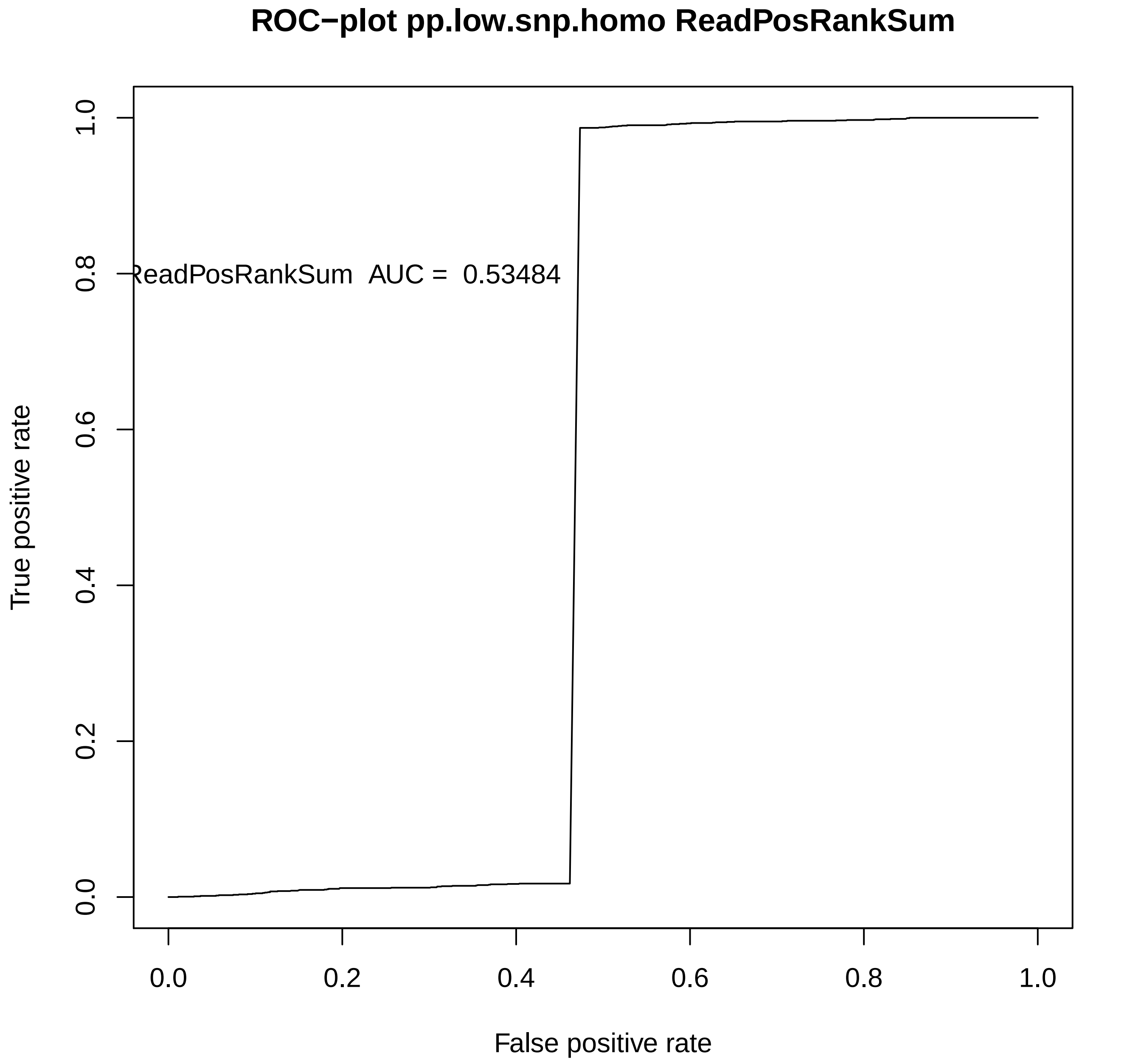
ROC−plot pp.low.snp.homo ReadPosRankSum
ReadPosRankSum AUC = 0.53484
True positive rate
False positive rate
0.0
0.2
0.4
0.6
0.8
1.0

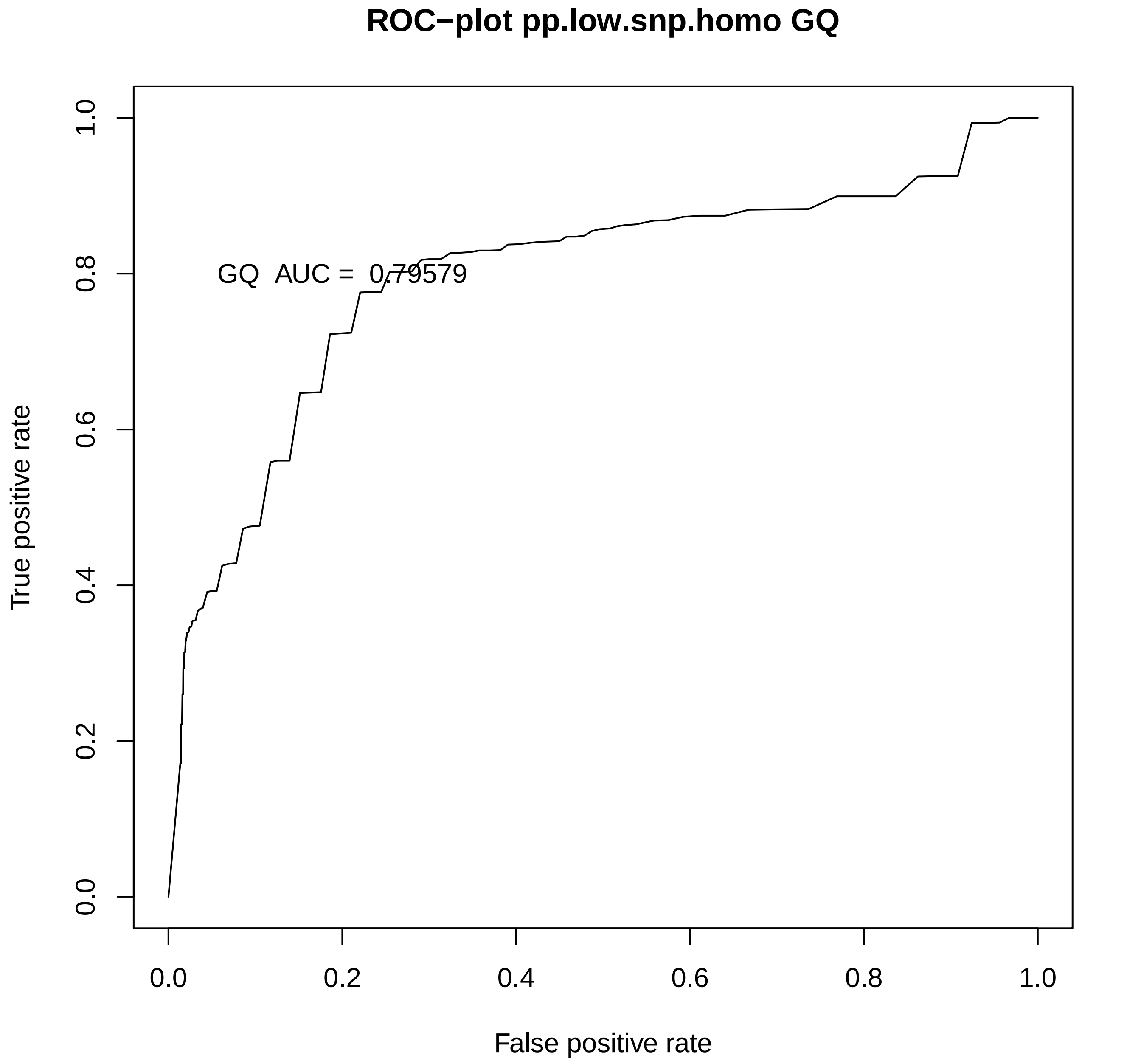
ROC−plot pp.low.snp.homo GQ
GQ AUC = 0.79579
True positive rate
False positive rate
0.0
0.2
0.4
0.6
0.8
1.0

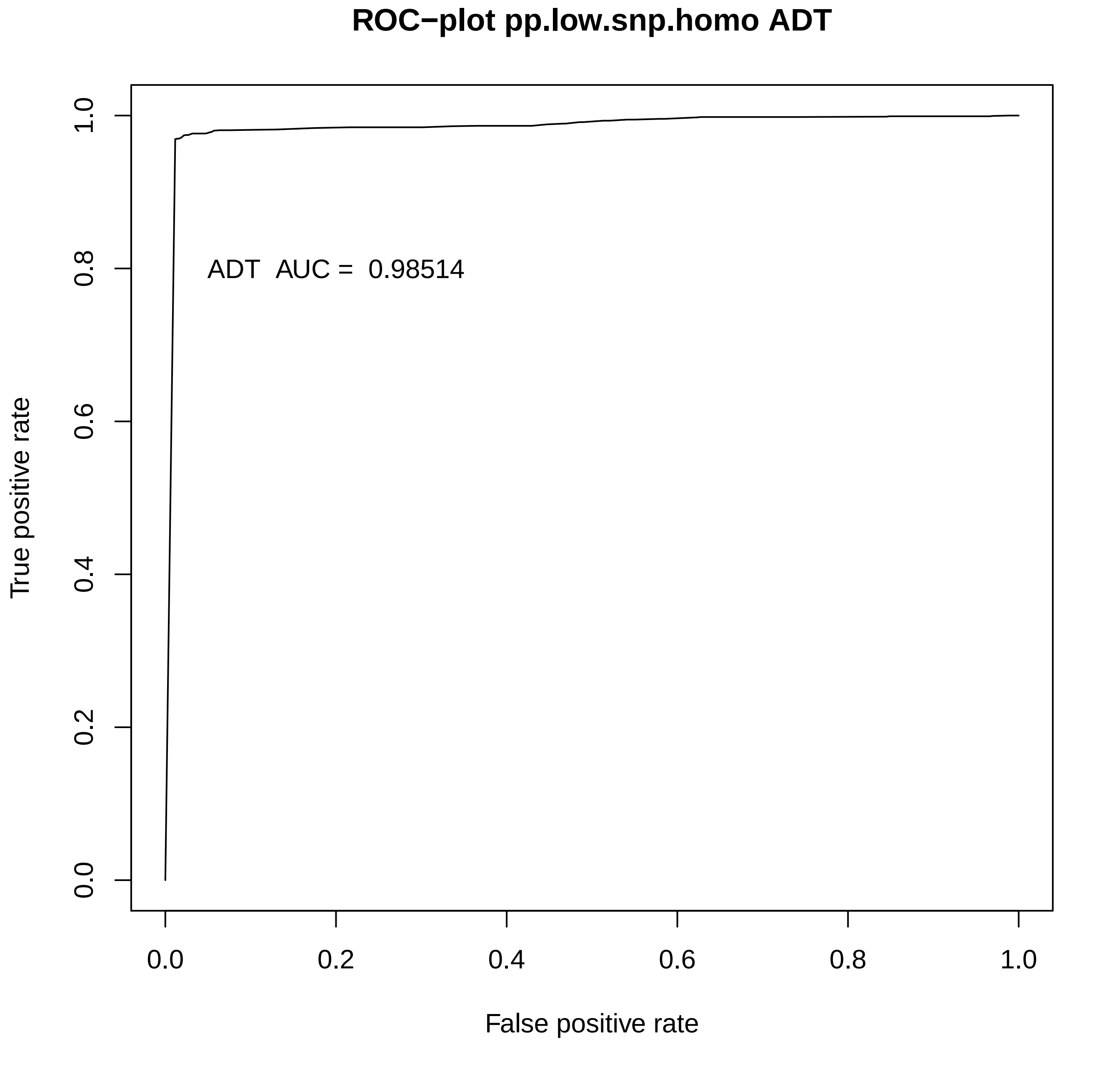

ROC−plot pp.low.snp.homo ADT
ADT AUC = 0.98514
True positive rate
False positive rate
0.0
0.2
0.4
0.6
0.8
1.0

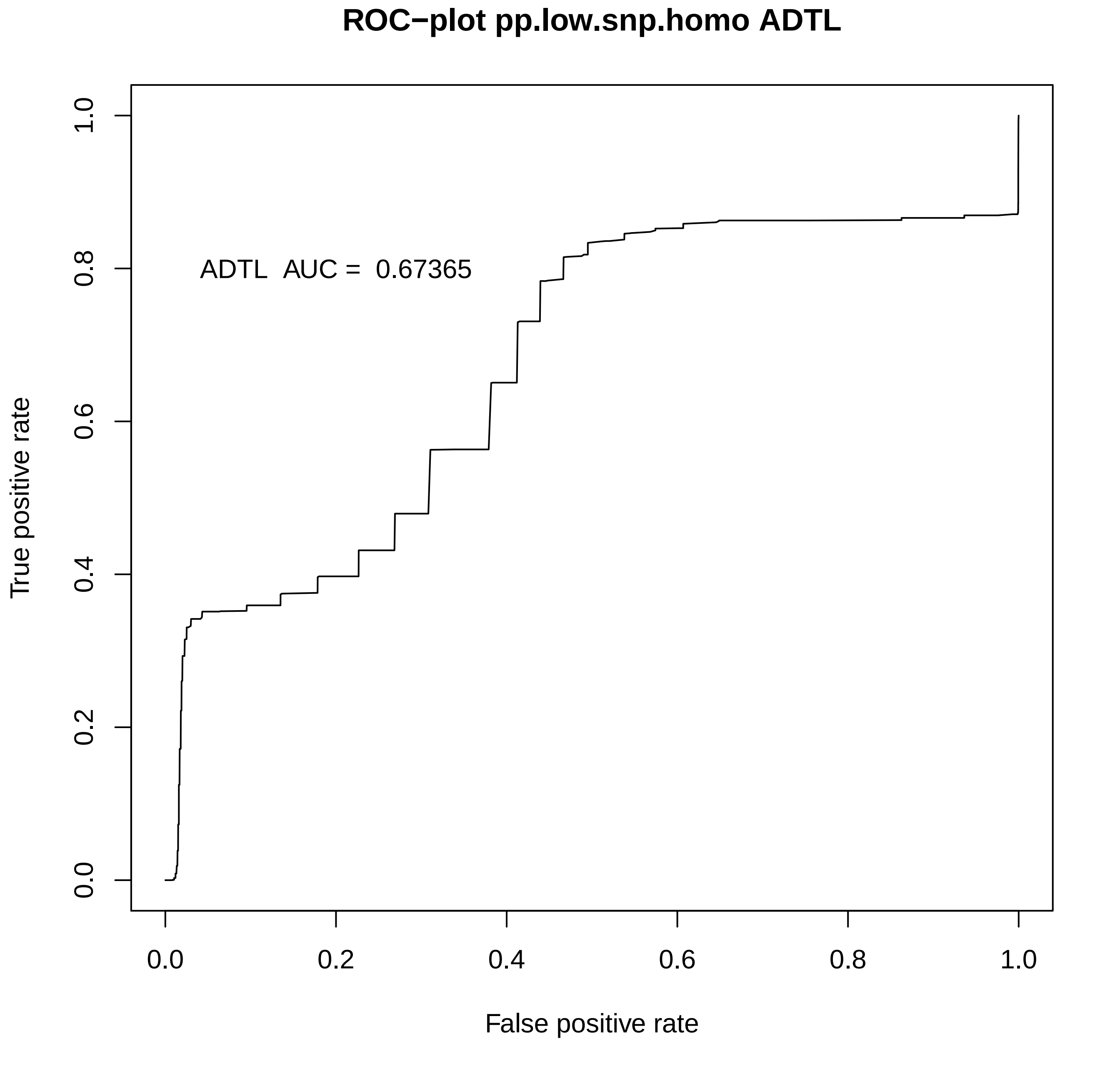
ROC−plot pp.low.snp.homo ADTL
ADTL AUC = 0.67365
True positive rate
False positive rate
0.0
0.2
0.4
0.6
0.8
1.0
0.0
0.2
0.4
0.6
0.8
1.0

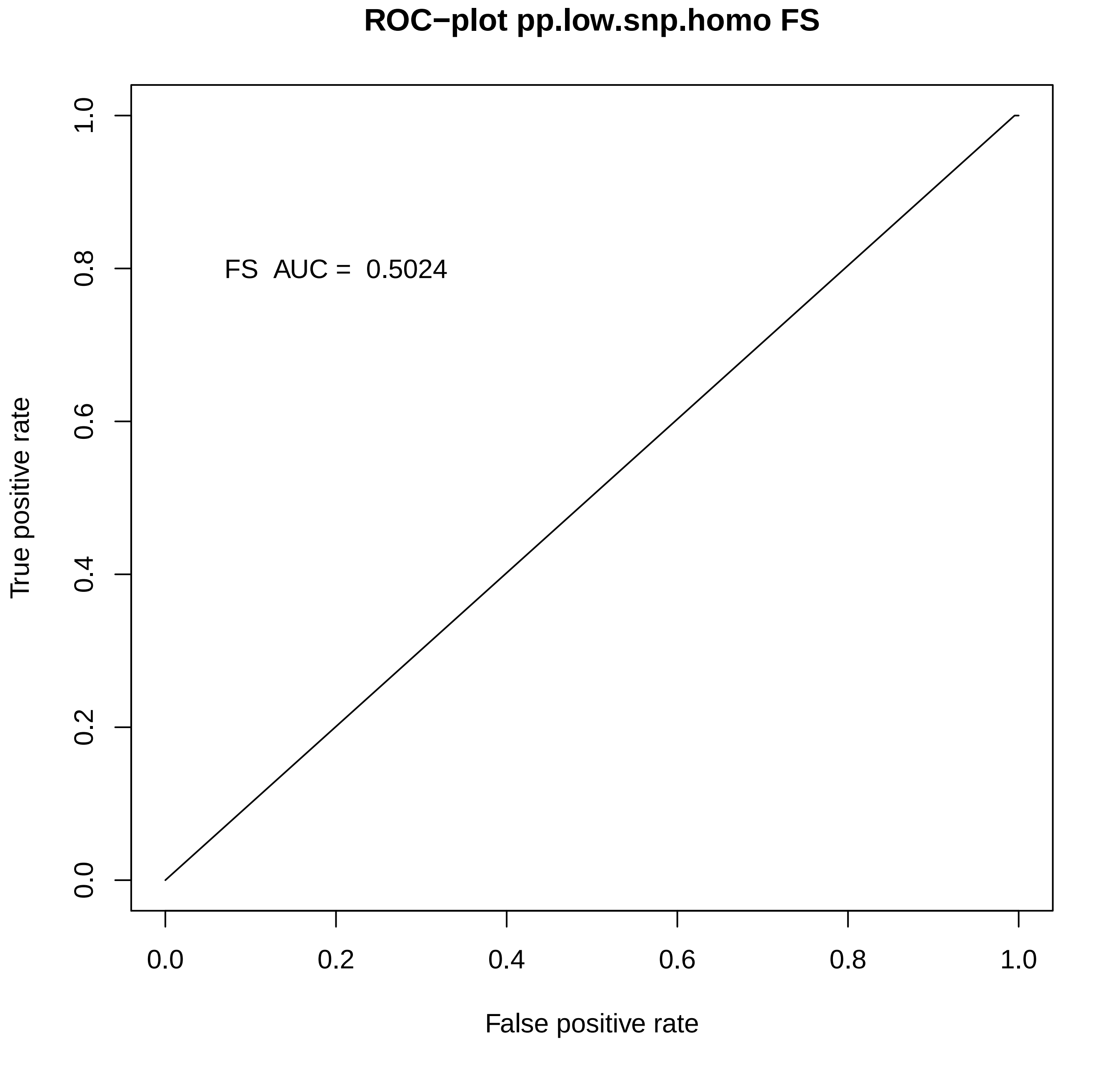
ROC−plot pp.low.snp.homo FS
FS AUC = 0.5024
True positive rate
False positive rate
0.0
0.2
0.4
0.6
0.8
1.0

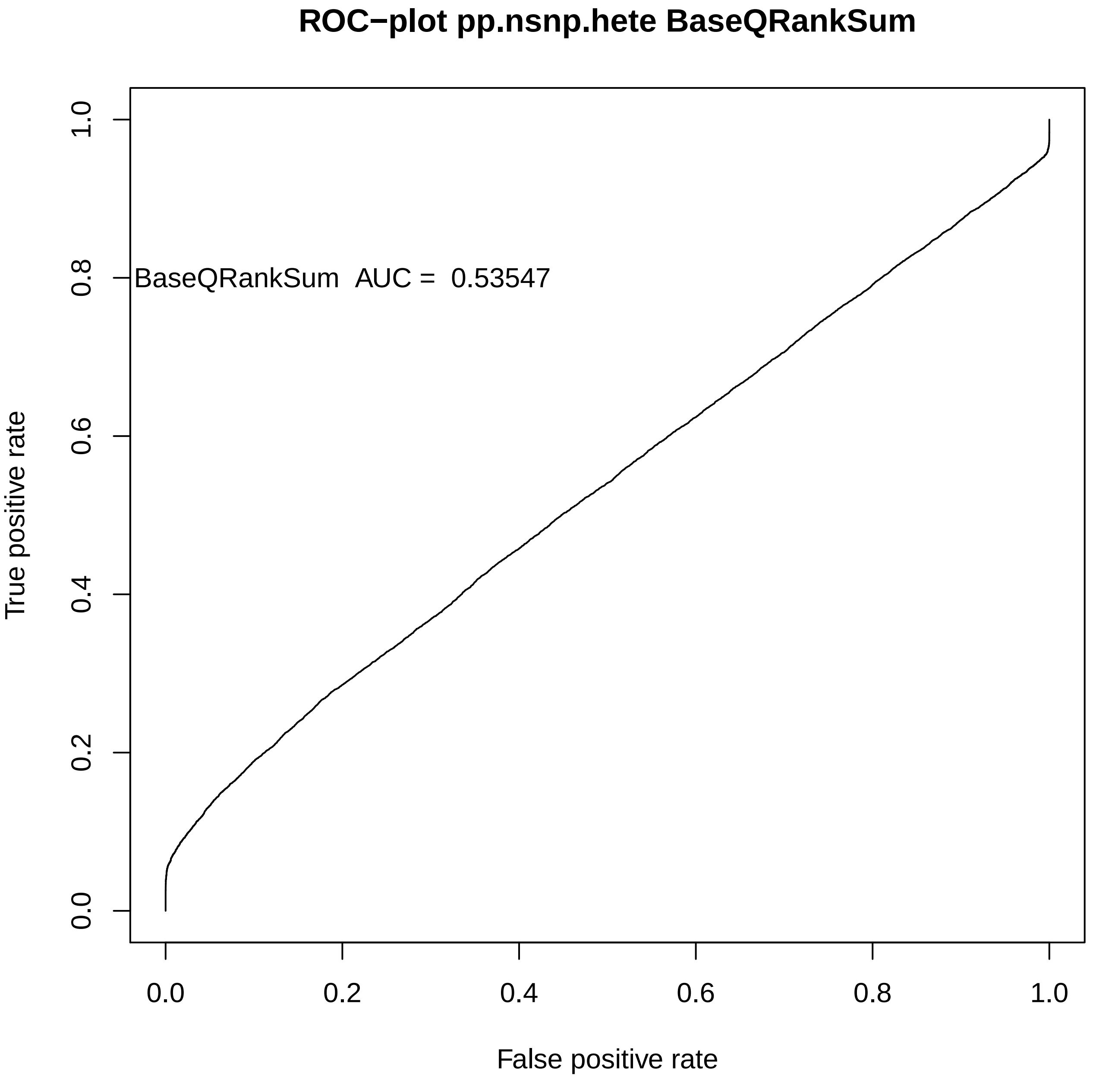

ROC−plot pp.nsnp.hete BaseQRankSum
BaseQRankSum AUC = 0.53547
True positive rate
False positive rate
0.0
0.2
0.4
0.6
0.8
1.0

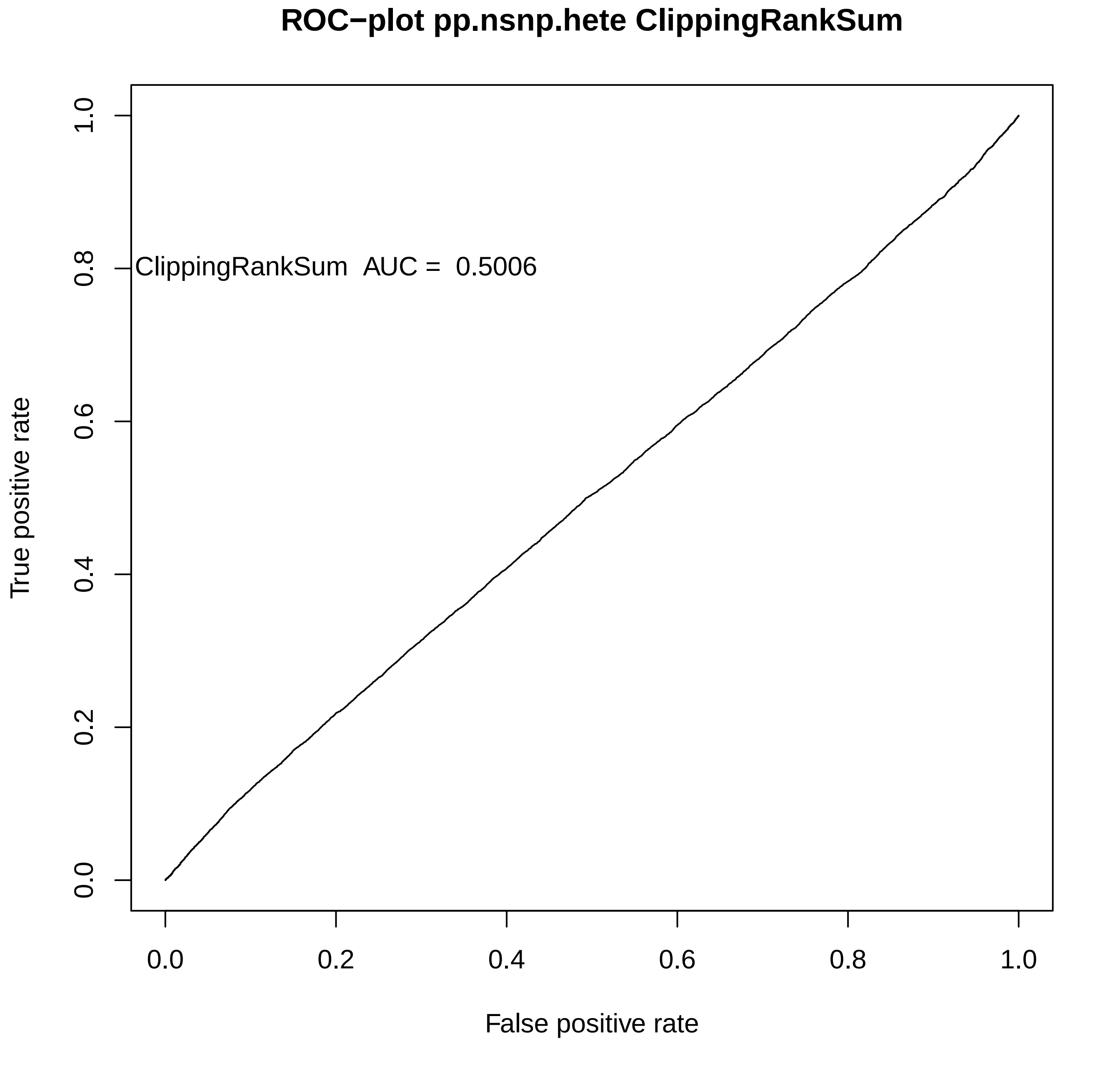
ROC−plot pp.nsnp.hete ClippingRankSum
ClippingRankSum AUC = 0.5006
True positive rate
False positive rate
0.0
0.2
0.4
0.6
0.8
1.0

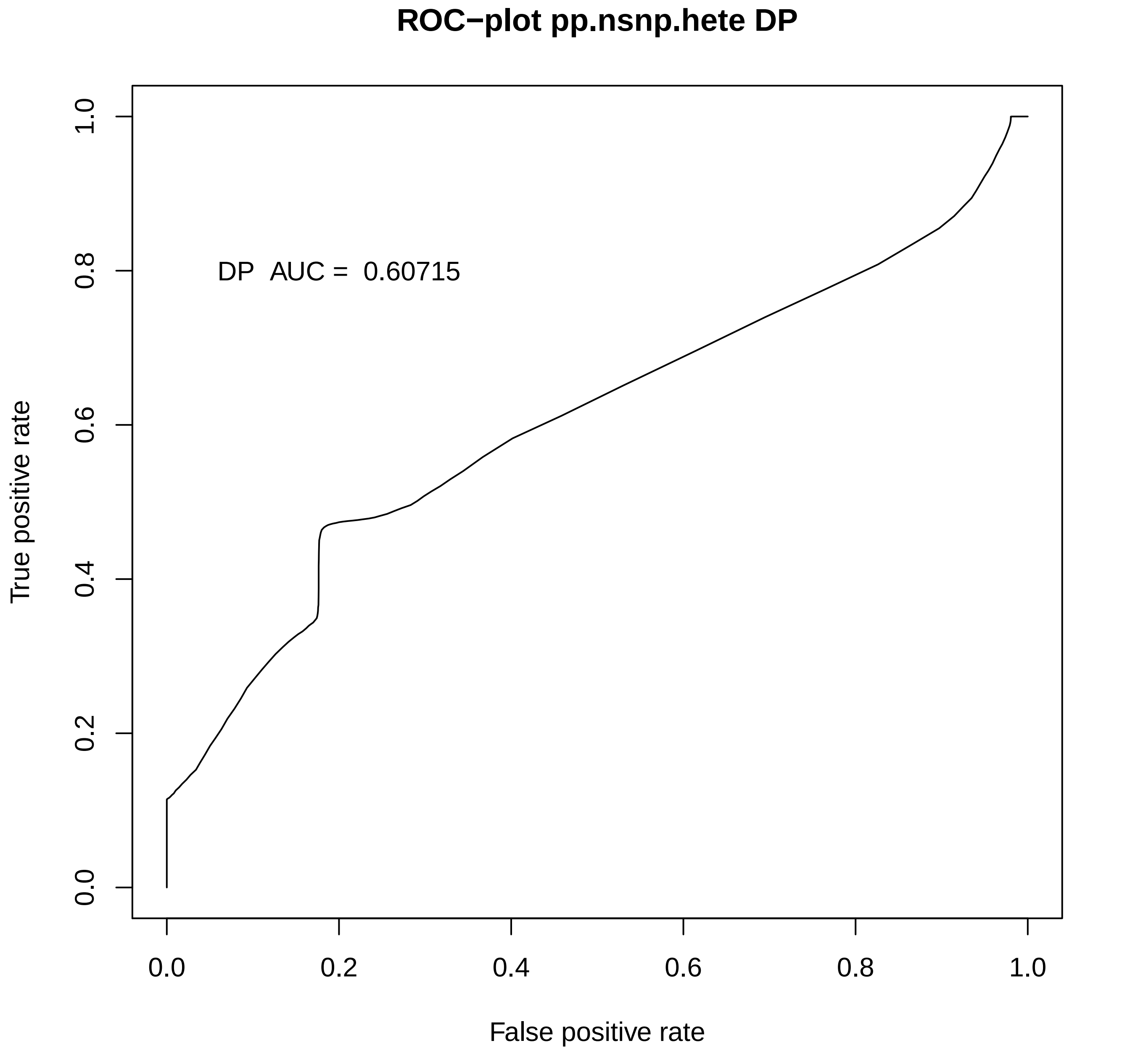
ROC−plot pp.nsnp.hete DP
DP AUC = 0.60715
True positive rate
False positive rate
0.0
0.2
0.4
0.6
0.8
1.0

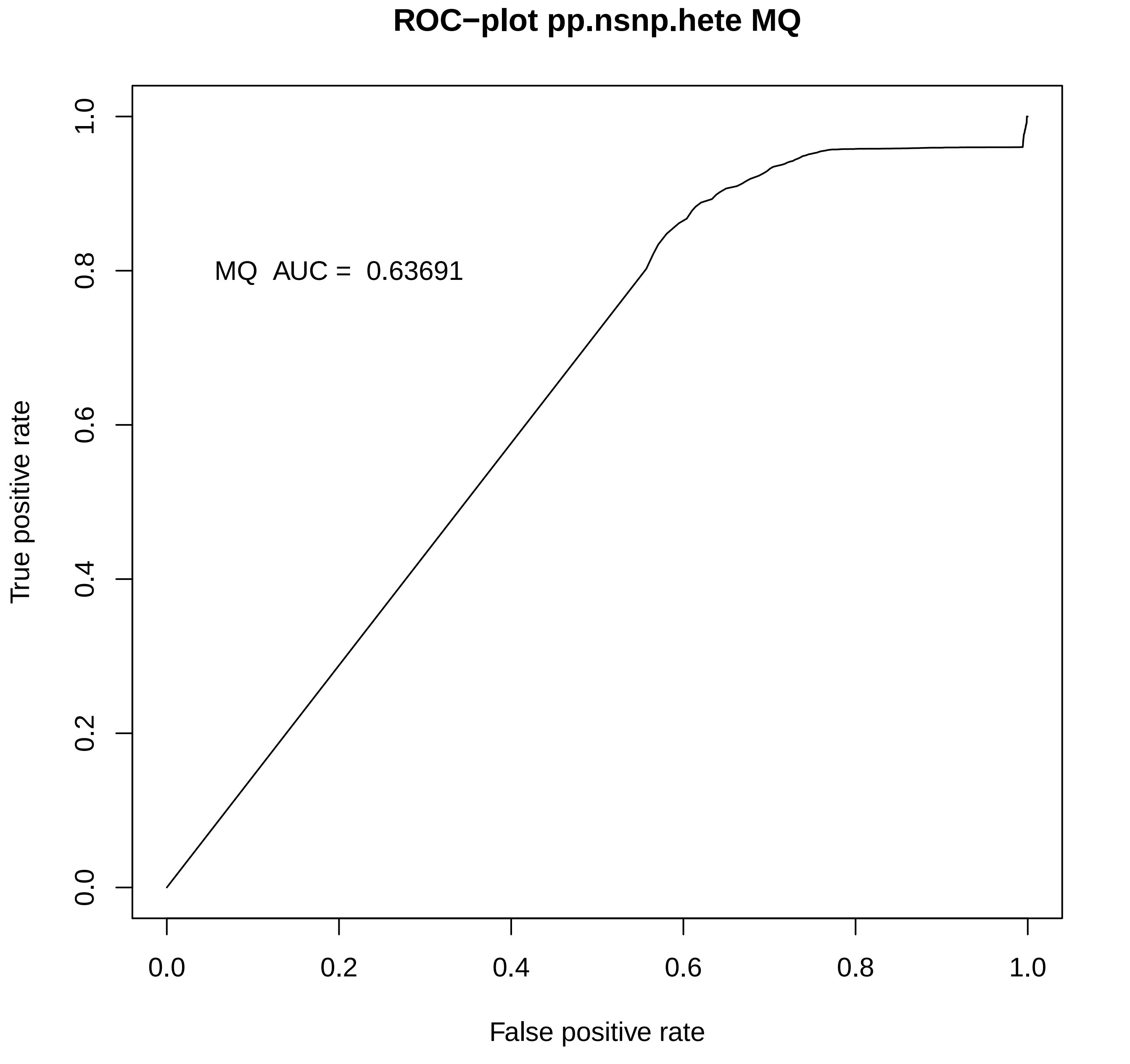
ROC−plot pp.nsnp.hete MQ
MQ AUC = 0.63691
True positive rate
False positive rate
0.0
0.2
0.4
0.6
0.8
1.0

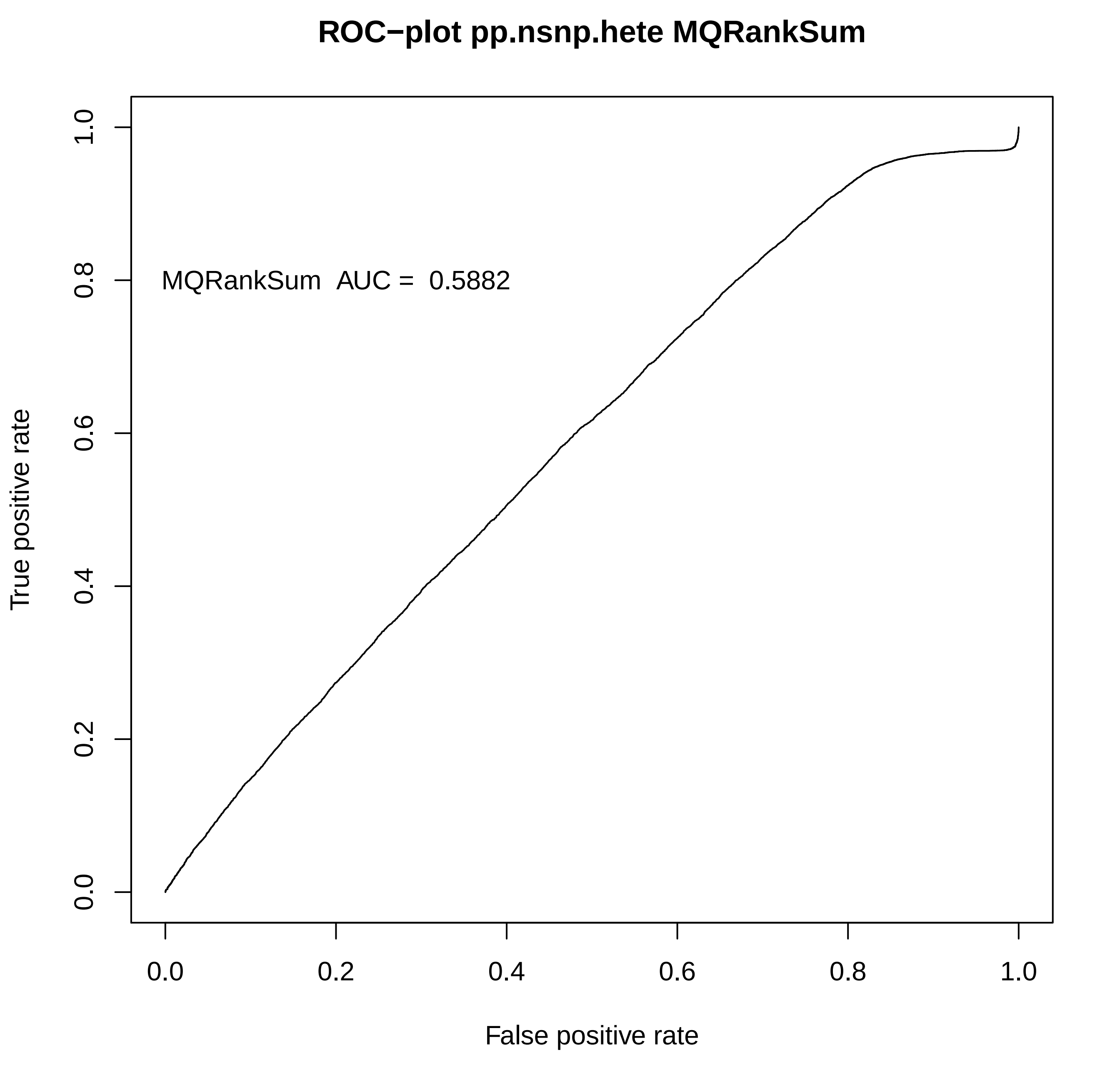
ROC−plot pp.nsnp.hete MQRankSum
MQRankSum AUC = 0.5882
True positive rate
False positive rate
0.0
0.2
0.4
0.6
0.8
1.0

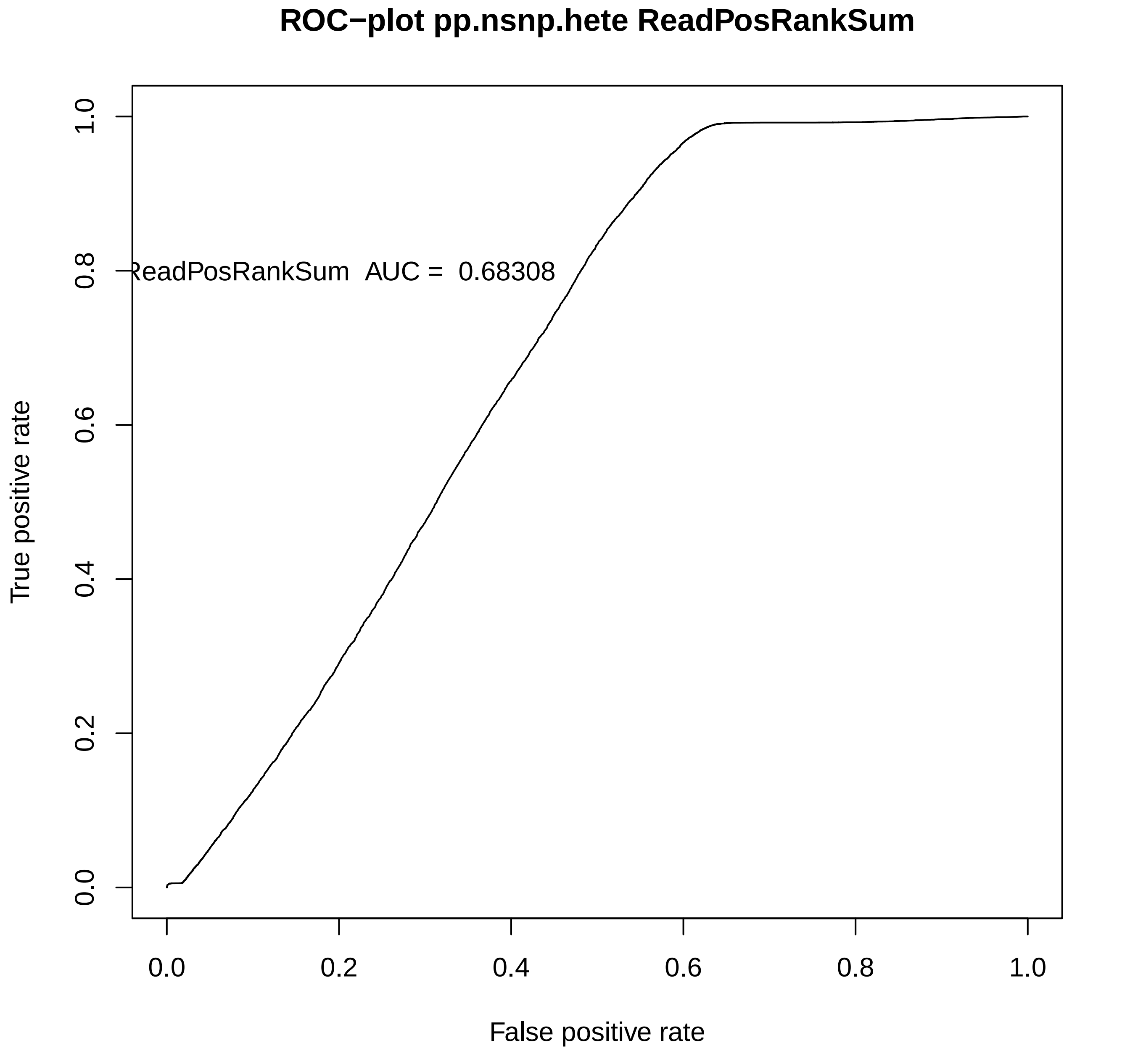
ROC−plot pp.nsnp.hete ReadPosRankSum
ReadPosRankSum AUC = 0.68308
True positive rate
False positive rate
0.0
0.2
0.4
0.6
0.8
1.0

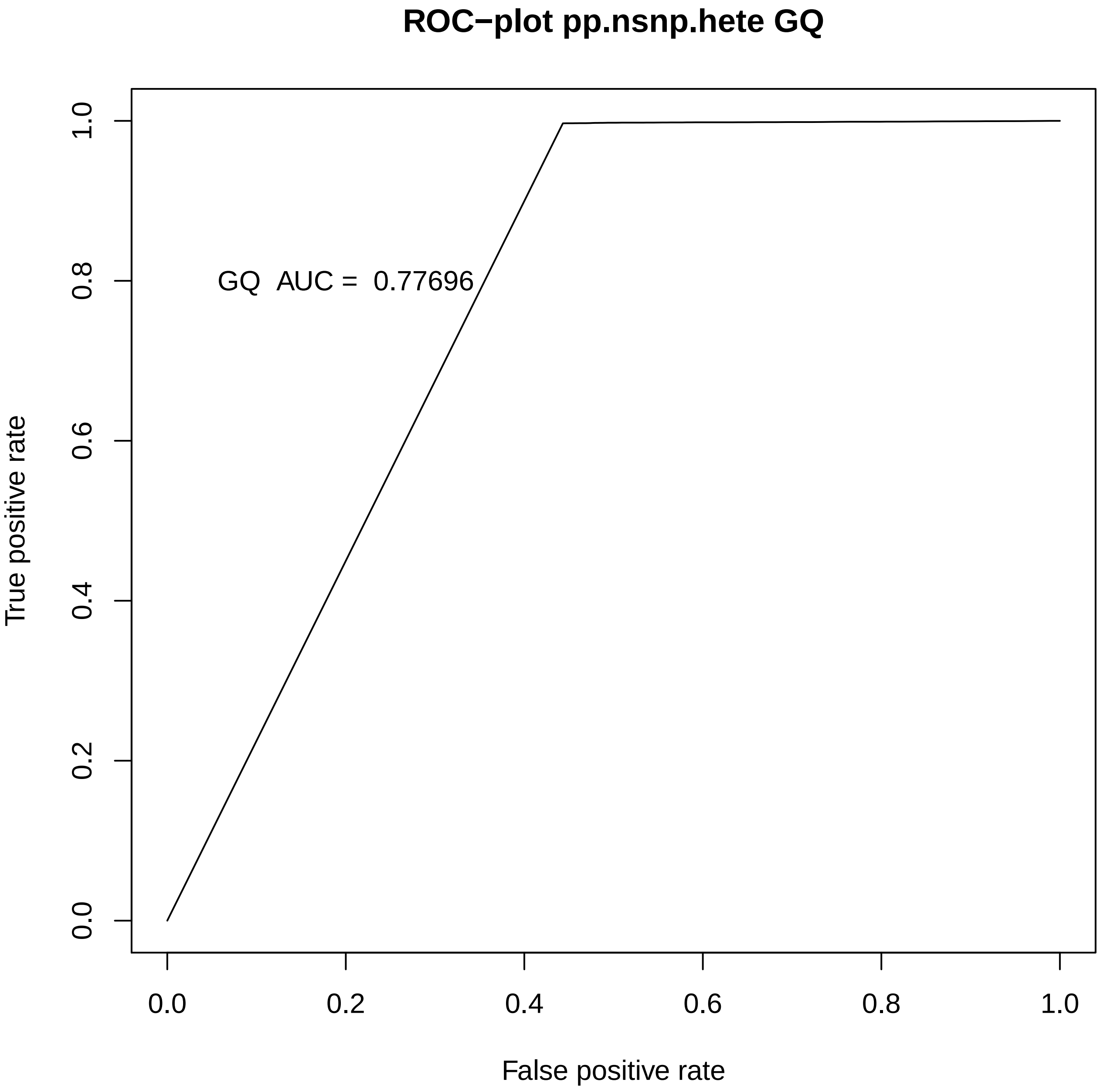
ROC−plot pp.nsnp.hete GQ
GQ AUC = 0.77696
True positive rate
False positive rate
0.0
0.2
0.4
0.6
0.8
1.0

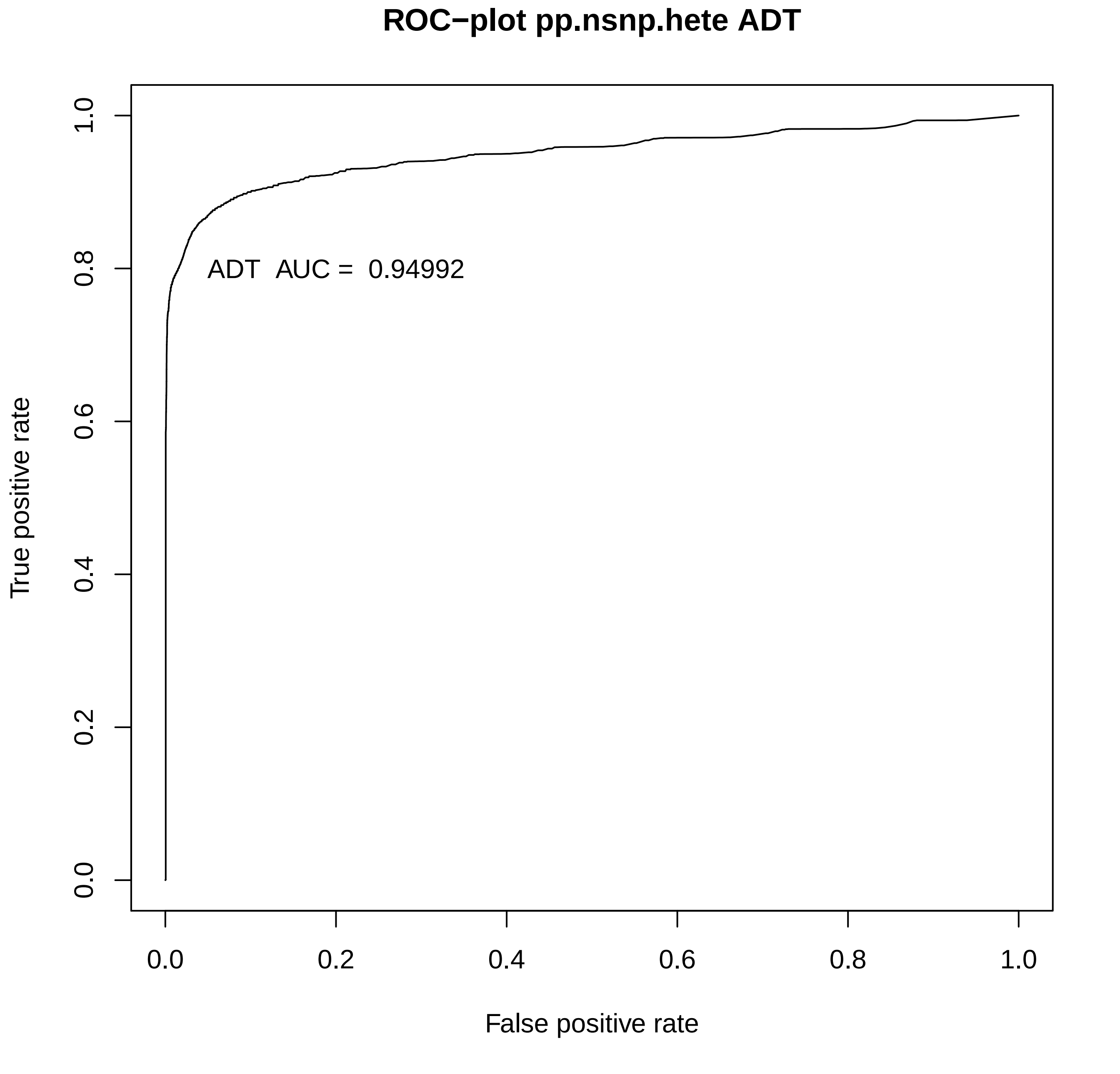
ROC−plot pp.nsnp.hete ADT
ADT AUC = 0.94992
True positive rate
False positive rate
0.0
0.2
0.4
0.6
0.8
1.0

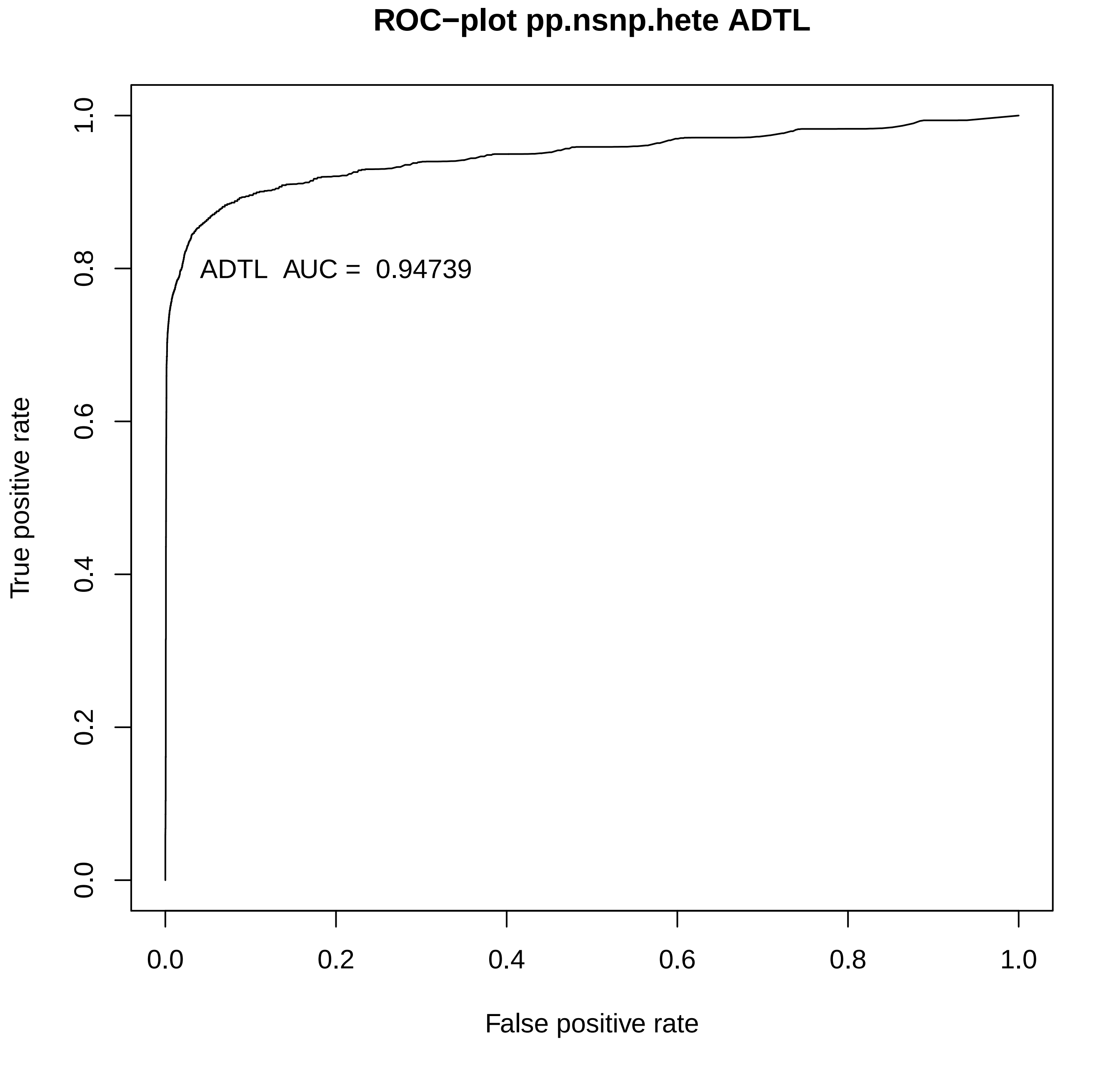
ROC−plot pp.nsnp.hete ADTL
ADTL AUC = 0.94739
True positive rate
False positive rate
0.0
0.2
0.4
0.6
0.8
1.0

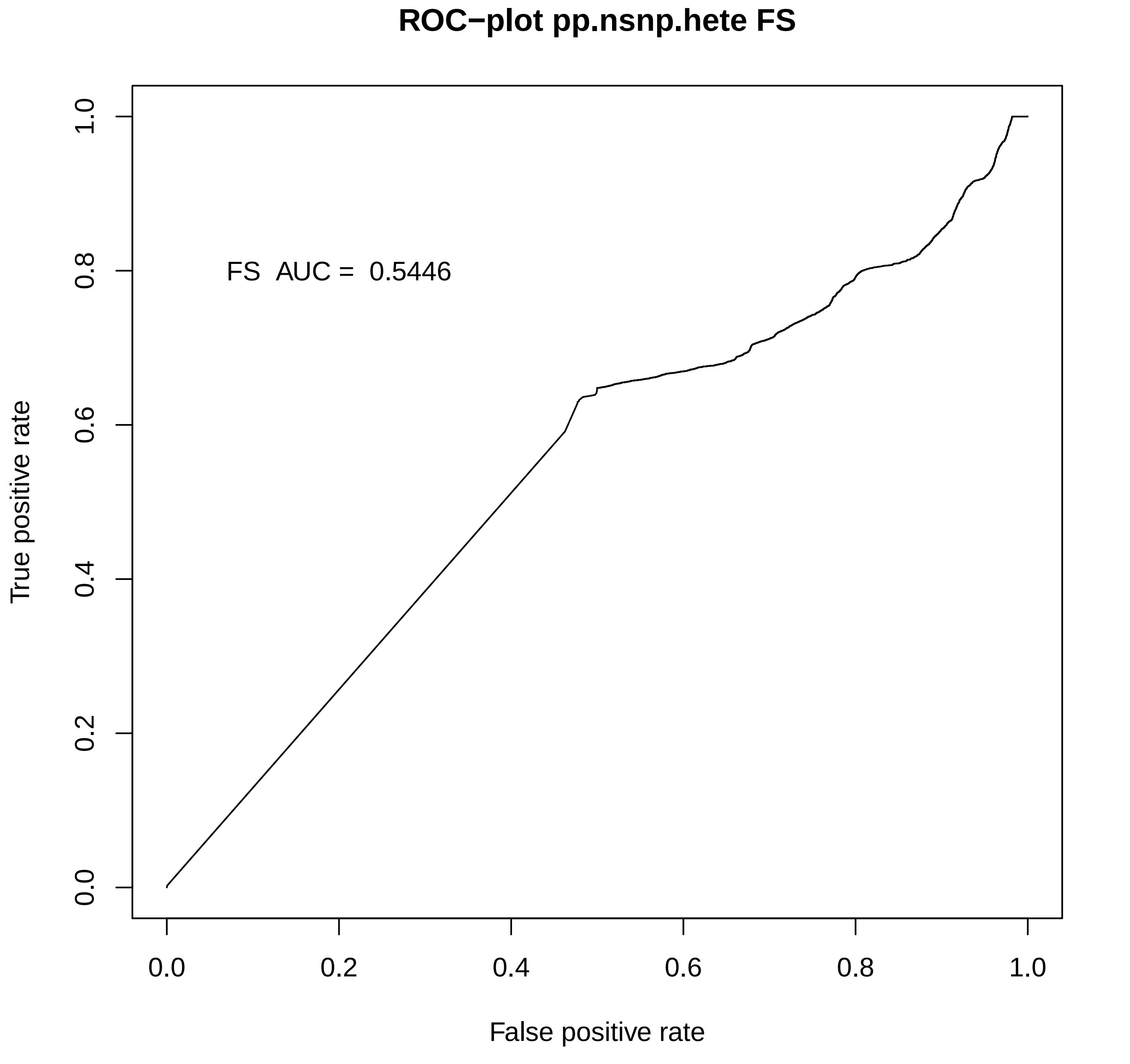
ROC−plot pp.nsnp.hete FS
FS AUC = 0.5446
True positive rate
False positive rate
0.0
0.2
0.4
0.6
0.8
1.0

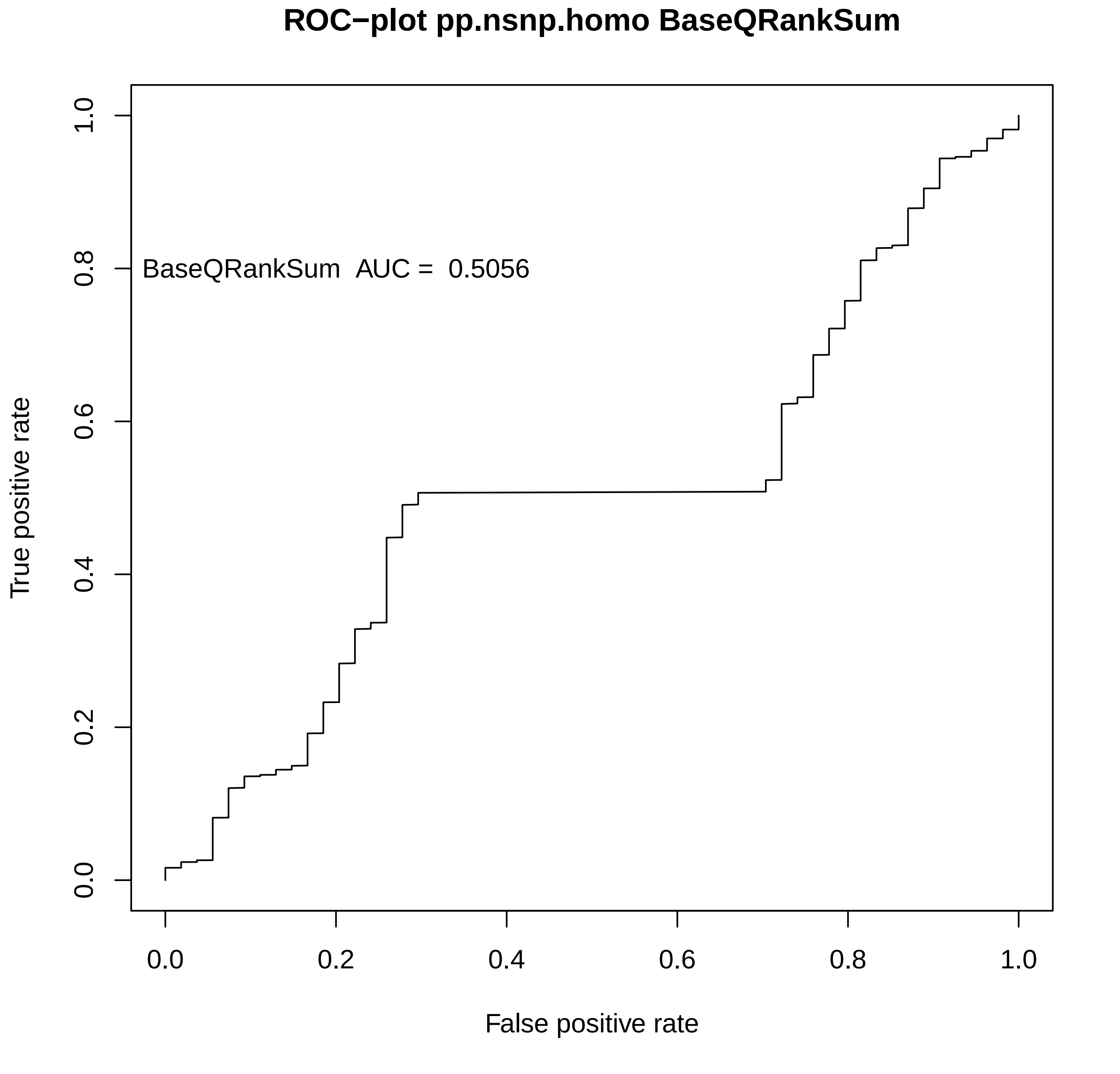
ROC−plot pp.nsnp.homo BaseQRankSum
BaseQRankSum AUC = 0.5056
True positive rate
False positive rate
0.0
0.2
0.4
0.6
0.8
1.0

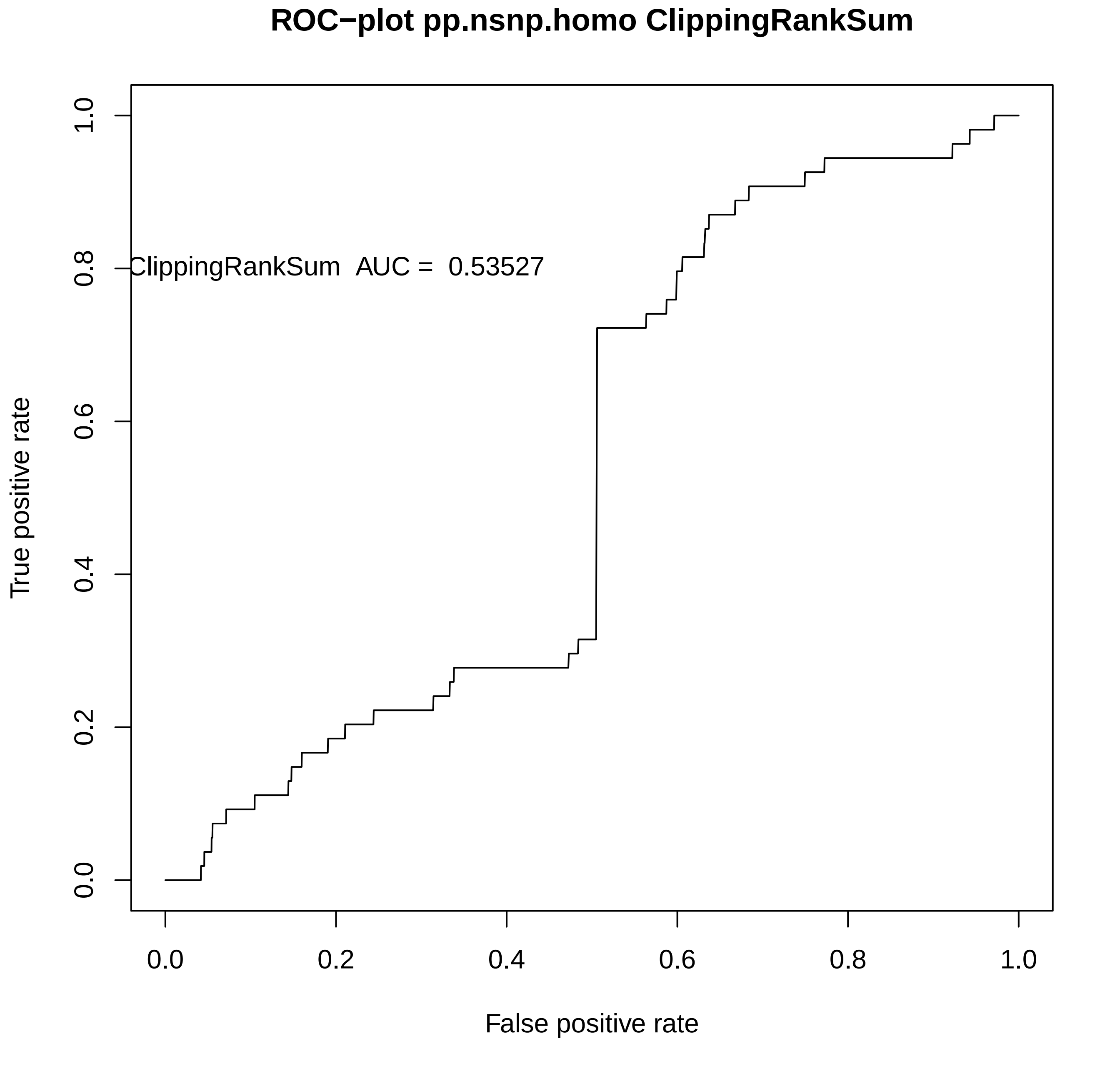
ROC−plot pp.nsnp.homo ClippingRankSum
ClippingRankSum AUC = 0.53527
True positive rate
False positive rate
0.0
0.2
0.4
0.6
0.8
1.0

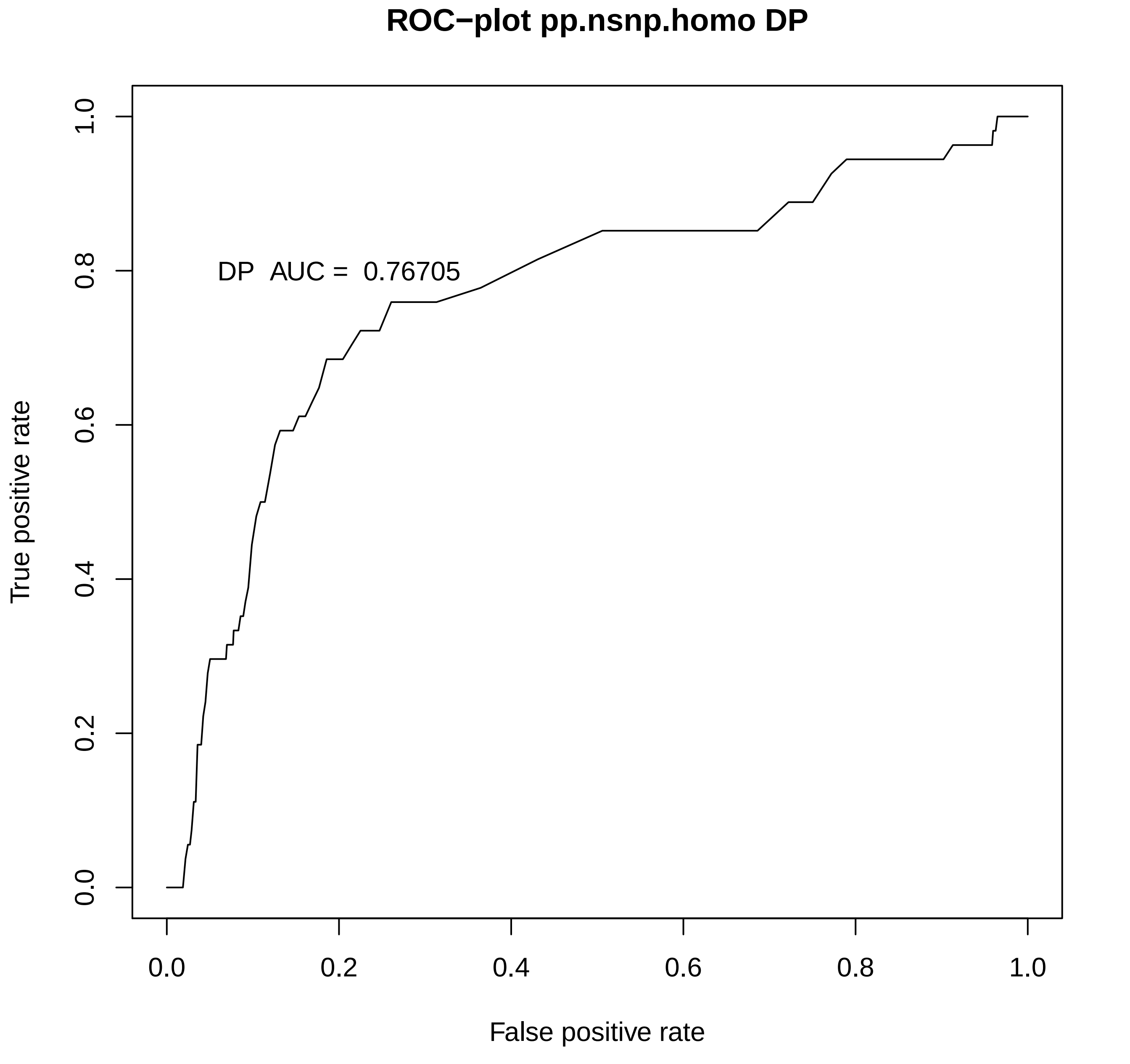
ROC−plot pp.nsnp.homo DP
DP AUC = 0.76705
True positive rate
False positive rate
0.0
0.2
0.4
0.6
0.8
1.0

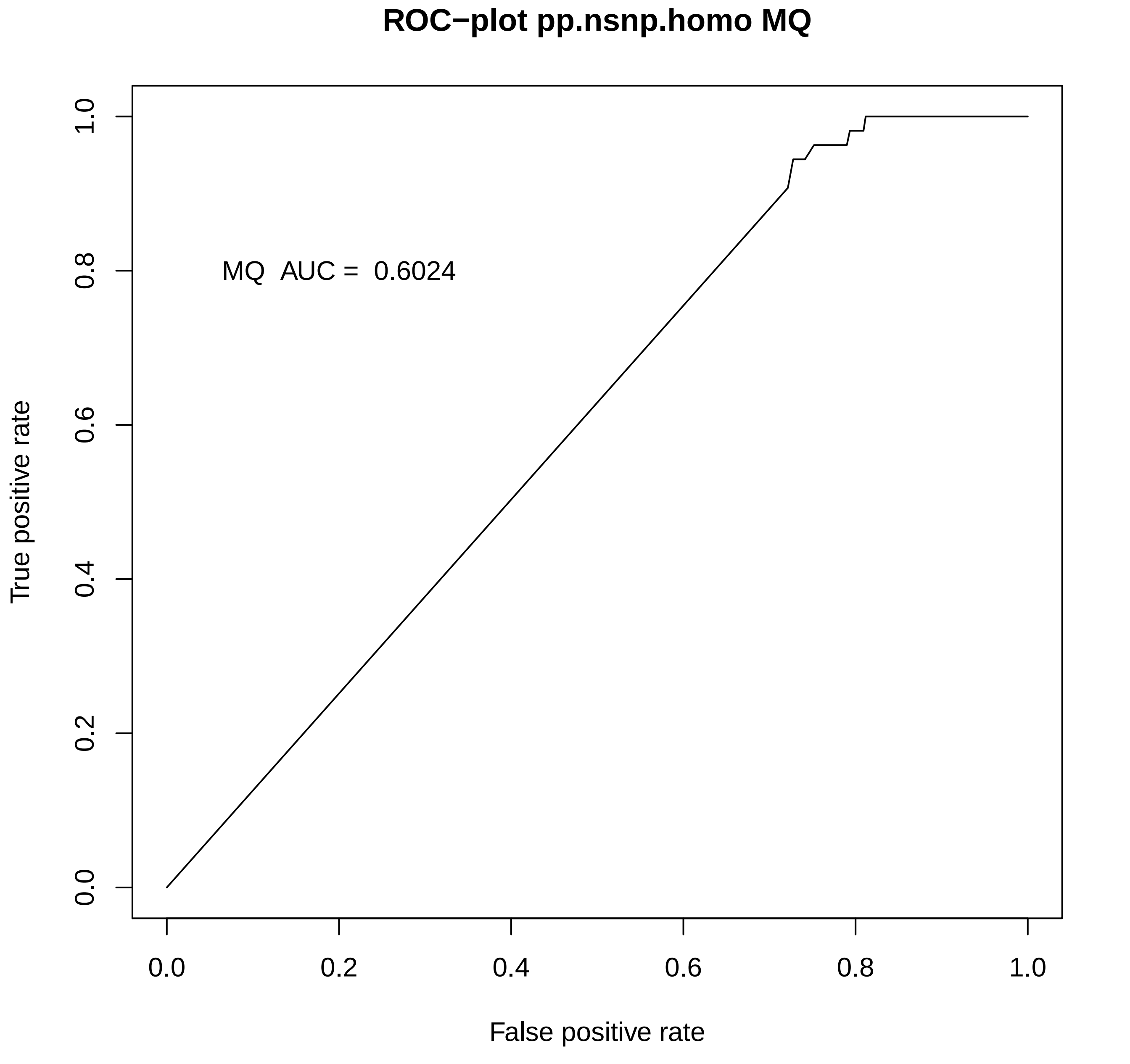
ROC−plot pp.nsnp.homo MQ
MQ AUC = 0.6024
True positive rate
False positive rate
0.0
0.2
0.4
0.6
0.8
1.0

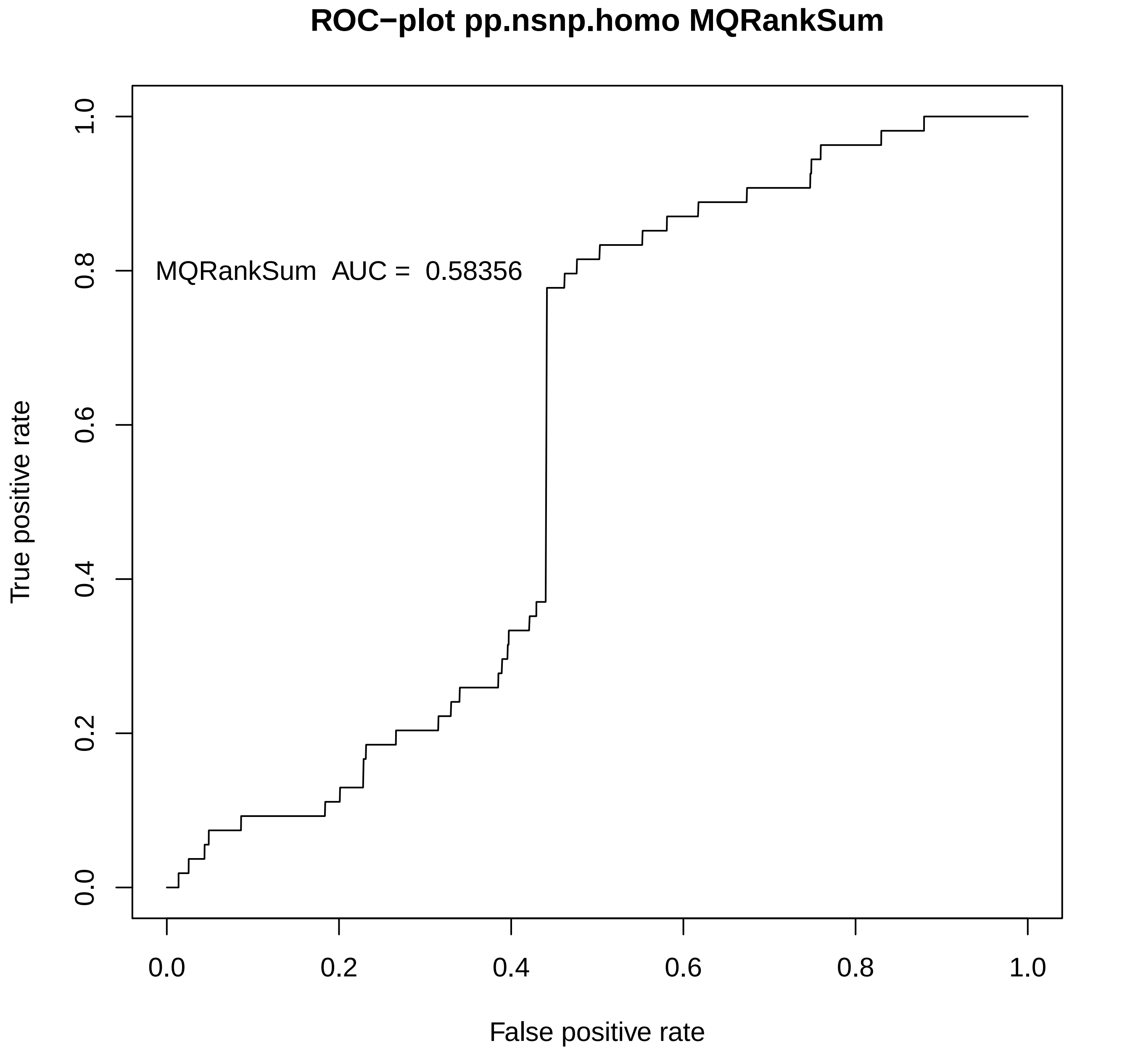
ROC−plot pp.nsnp.homo MQRankSum
MQRankSum AUC = 0.58356
True positive rate
False positive rate
0.0
0.2
0.4
0.6
0.8
1.0

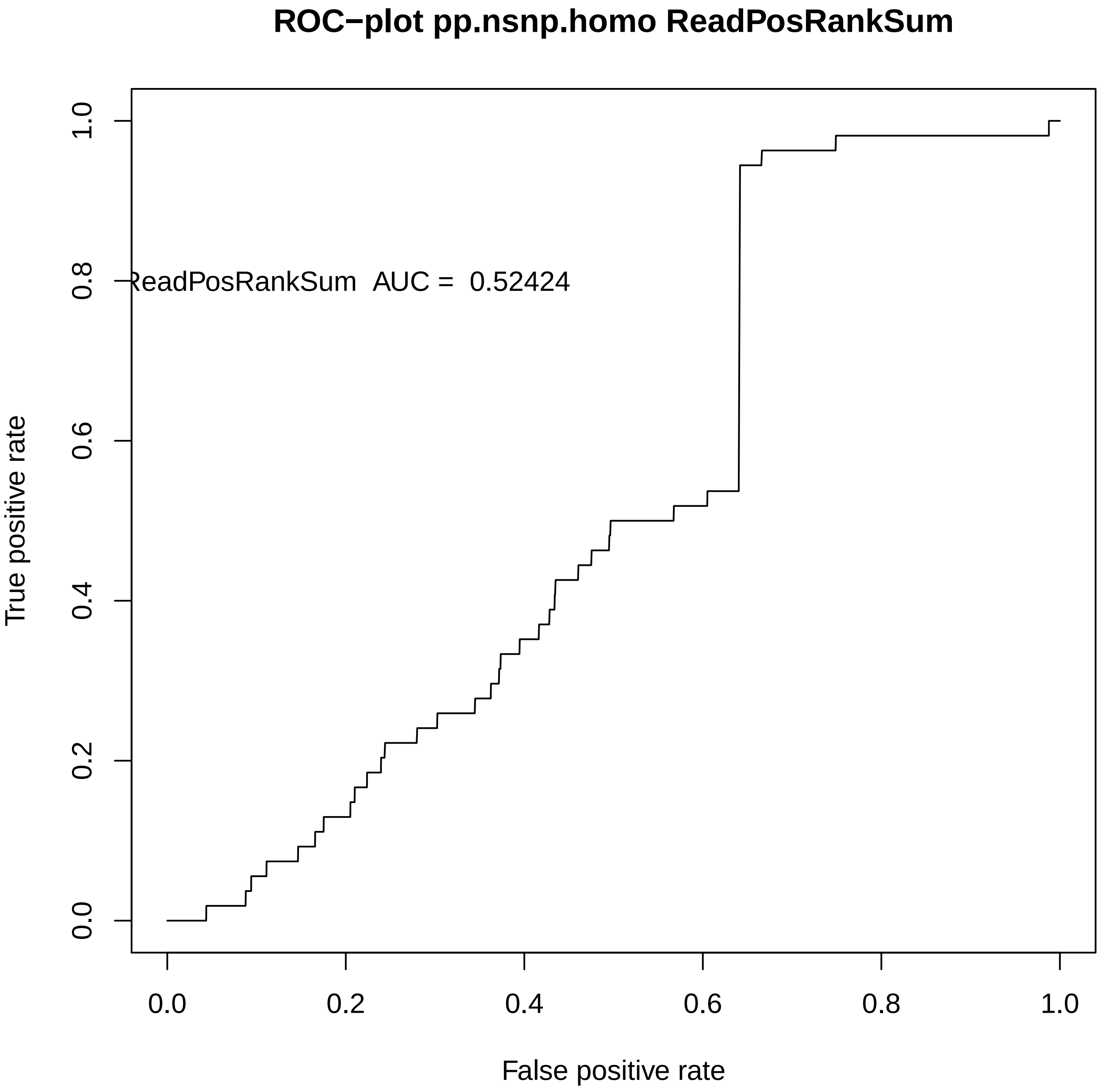
ROC−plot pp.nsnp.homo ReadPosRankSum
ReadPosRankSum AUC = 0.52424
True positive rate
False positive rate
0.0
0.2
0.4
0.6
0.8
1.0

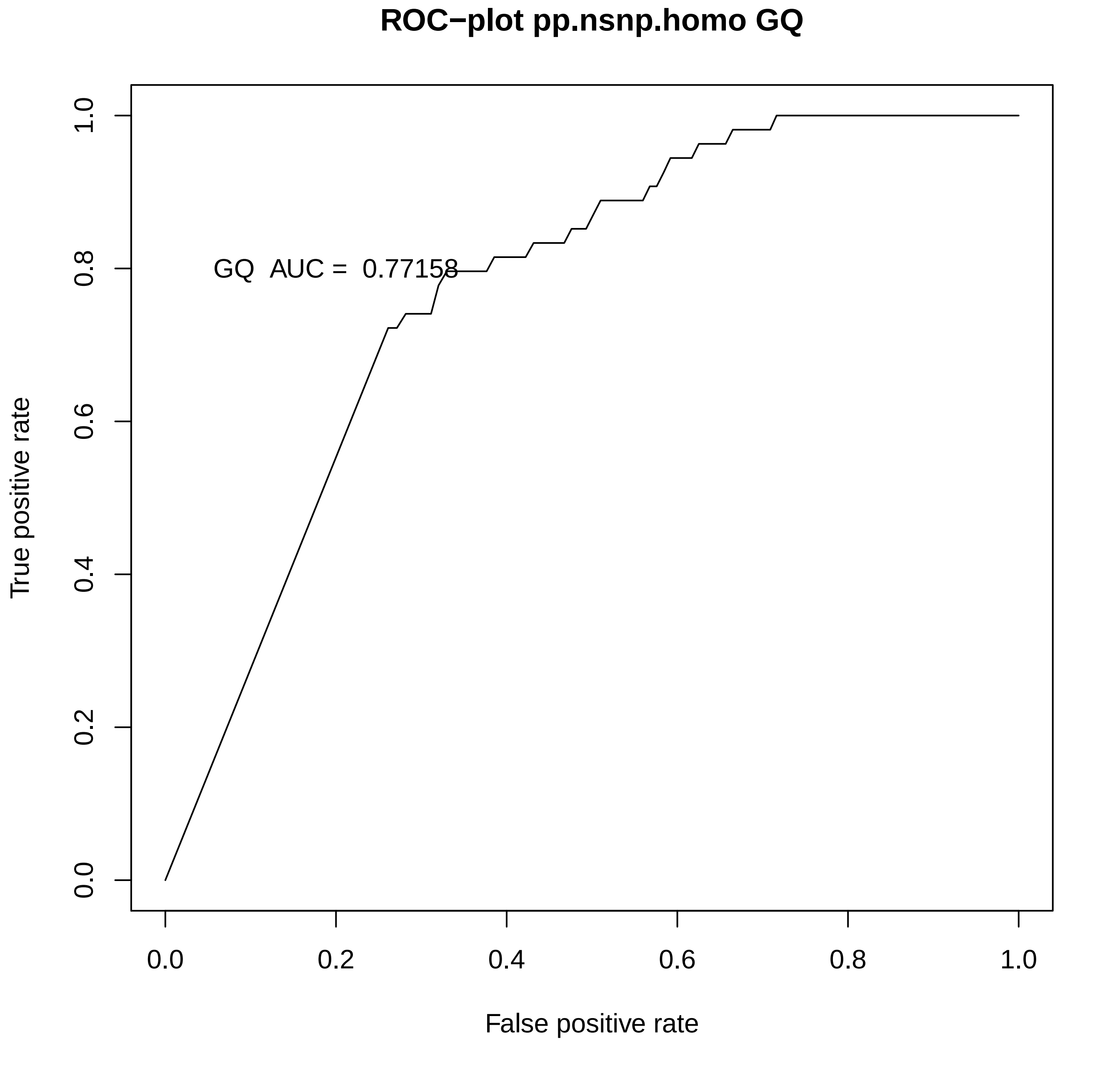

ROC−plot pp.nsnp.homo GQ
GQ AUC = 0.77158
True positive rate
False positive rate
0.0
0.2
0.4
0.6
0.8
1.0

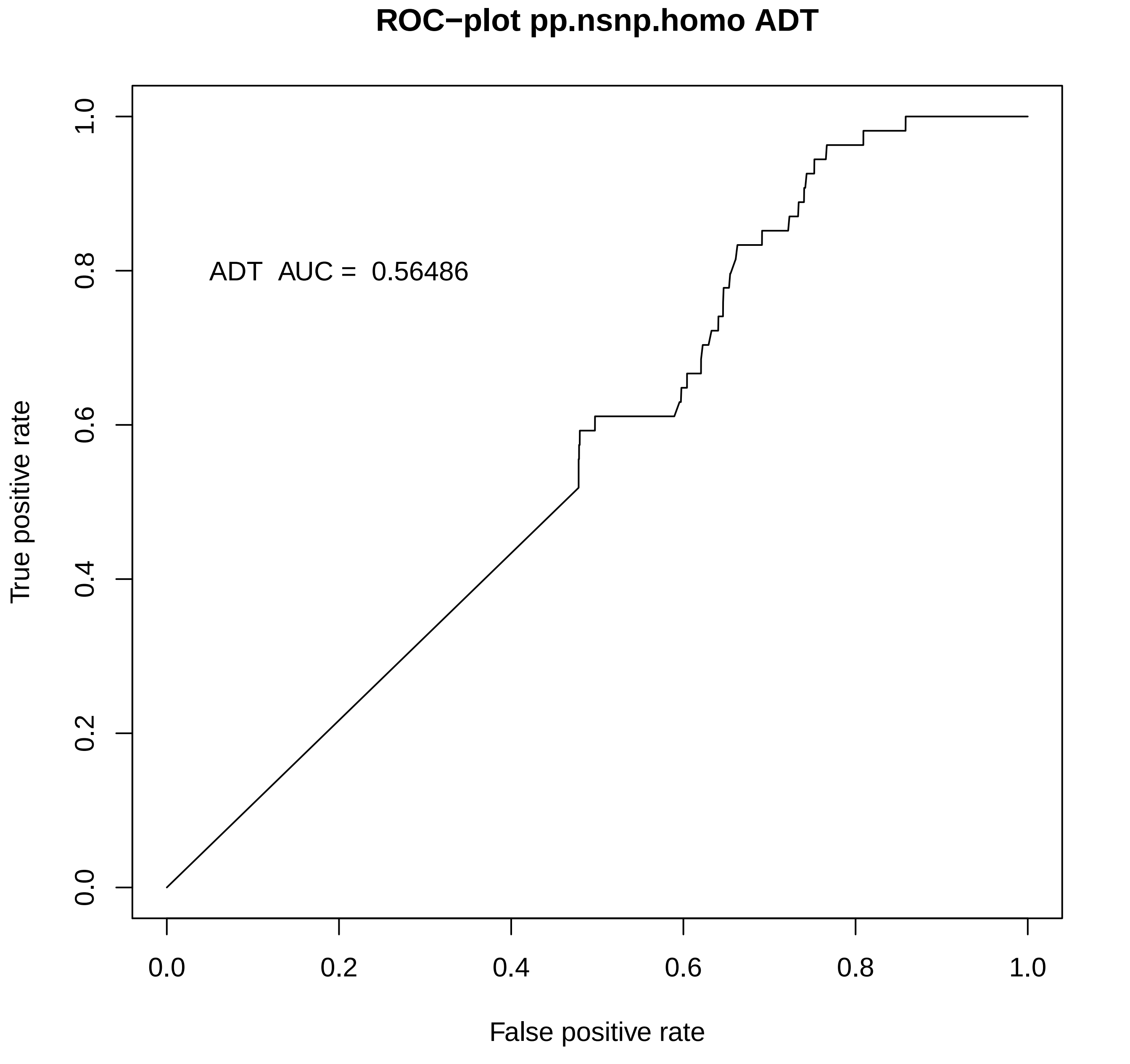
ROC−plot pp.nsnp.homo ADT
ADT AUC = 0.56486
True positive rate
False positive rate
0.0
0.2
0.4
0.6
0.8
1.0

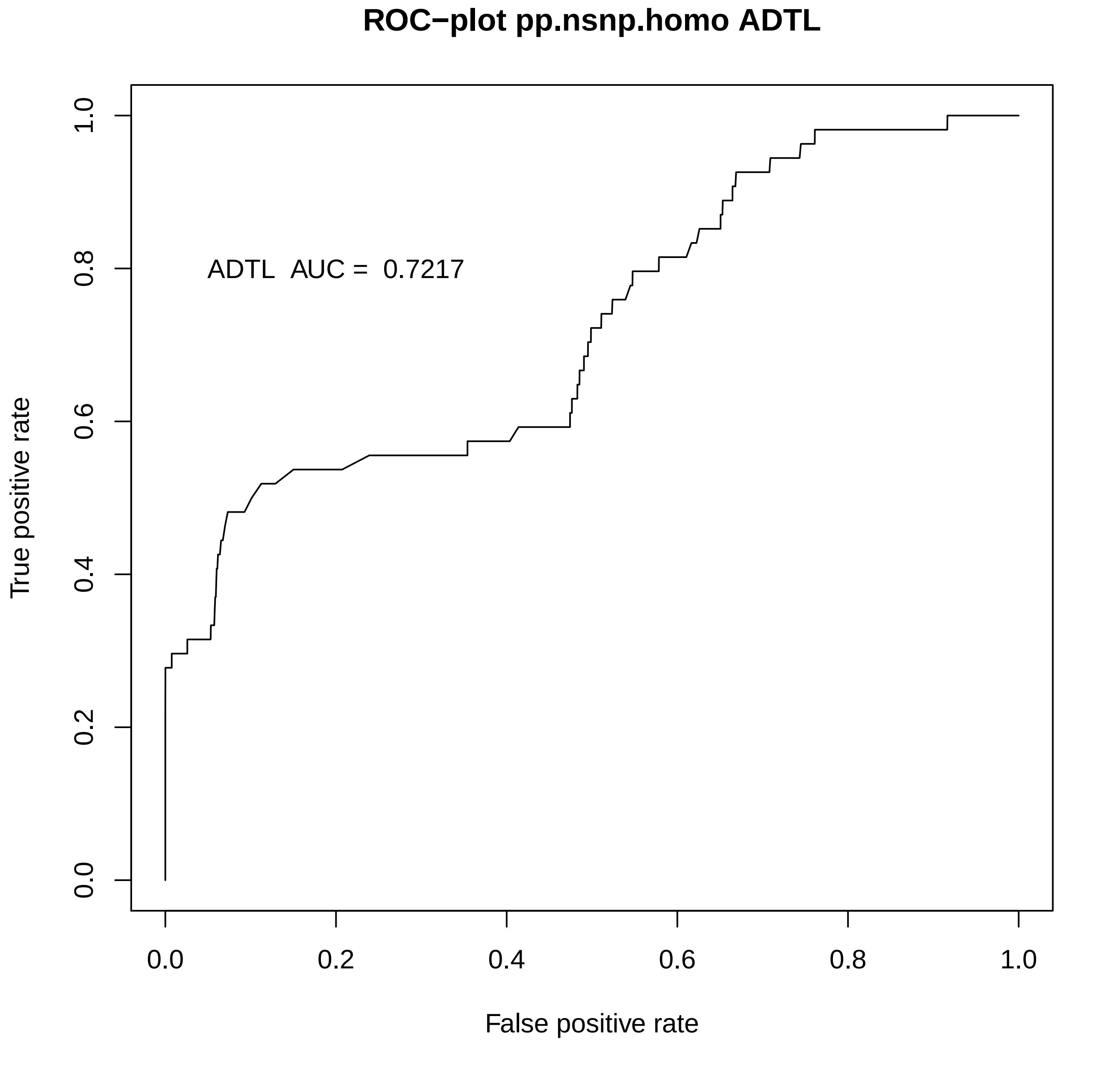

ROC−plot pp.nsnp.homo ADTL
ADTL AUC = 0.7217
True positive rate
False positive rate
0.0
0.2
0.4
0.6
0.8
1.0

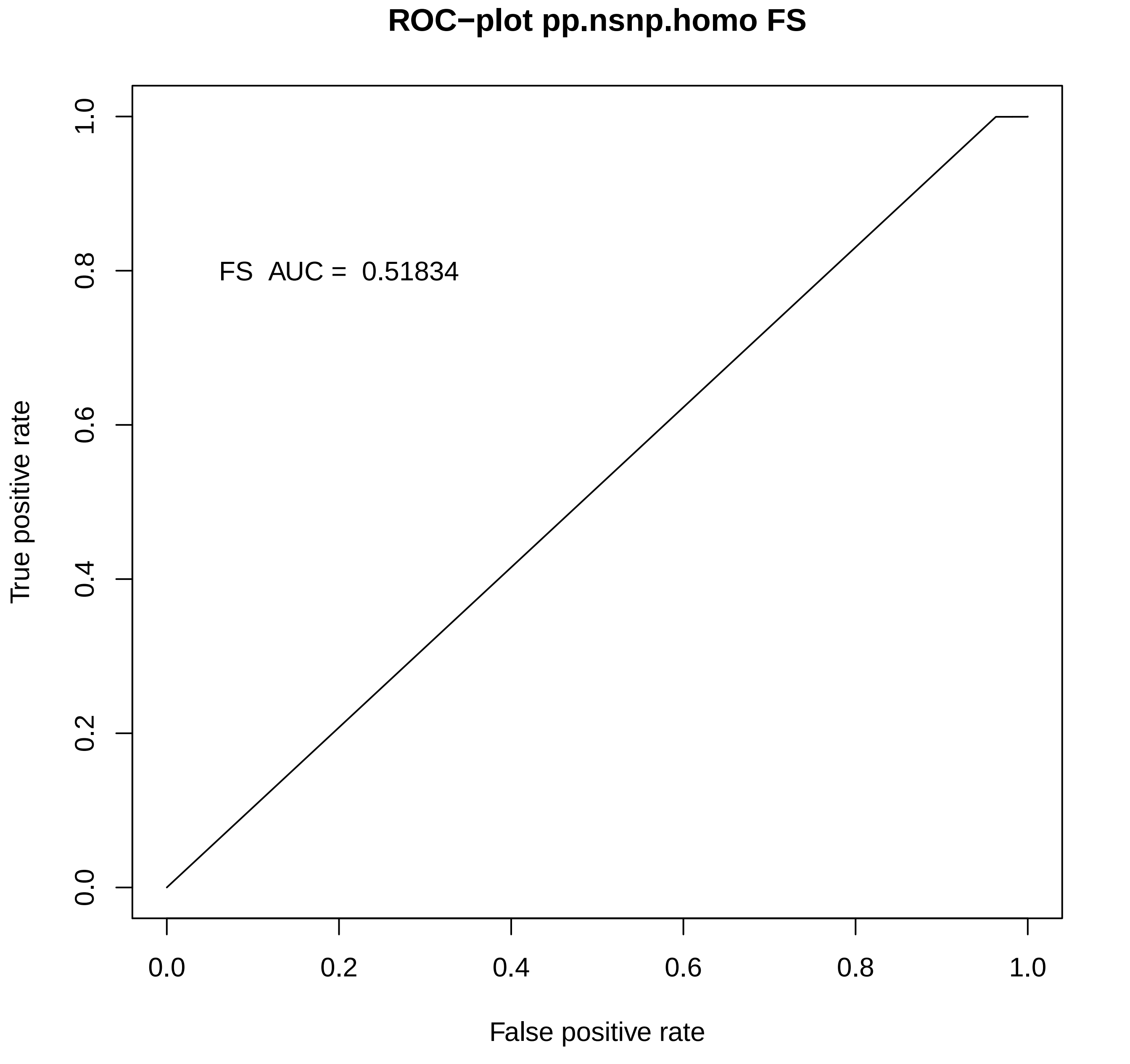
ROC−plot pp.nsnp.homo FS
FS AUC = 0.51834
True positive rate
False positive rate
0.0
0.2
0.4
0.6
0.8
1.0

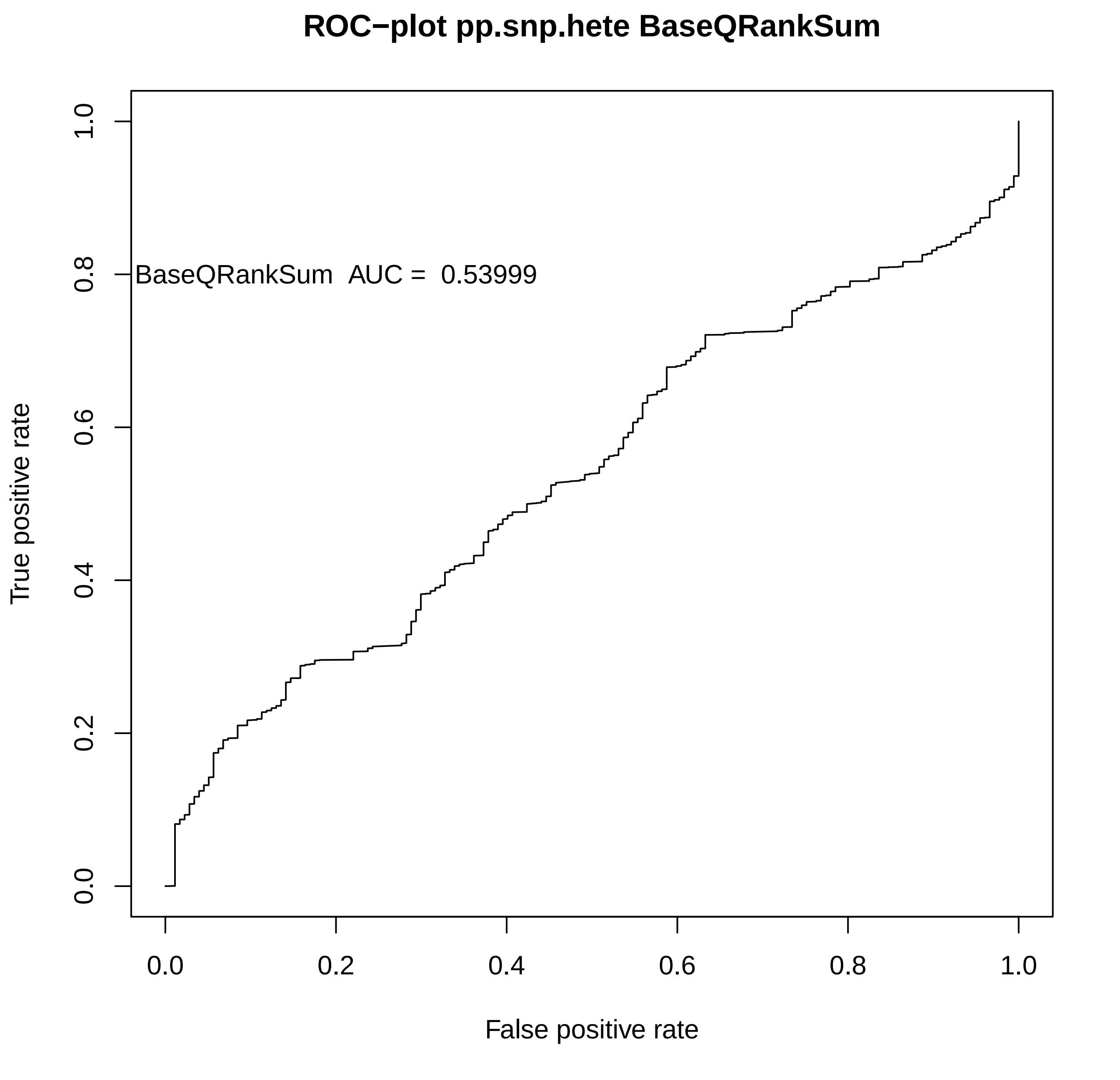
ROC−plot pp.snp.hete BaseQRankSum
BaseQRankSum AUC = 0.53999
True positive rate
False positive rate
0.0
0.2
0.4
0.6
0.8
1.0

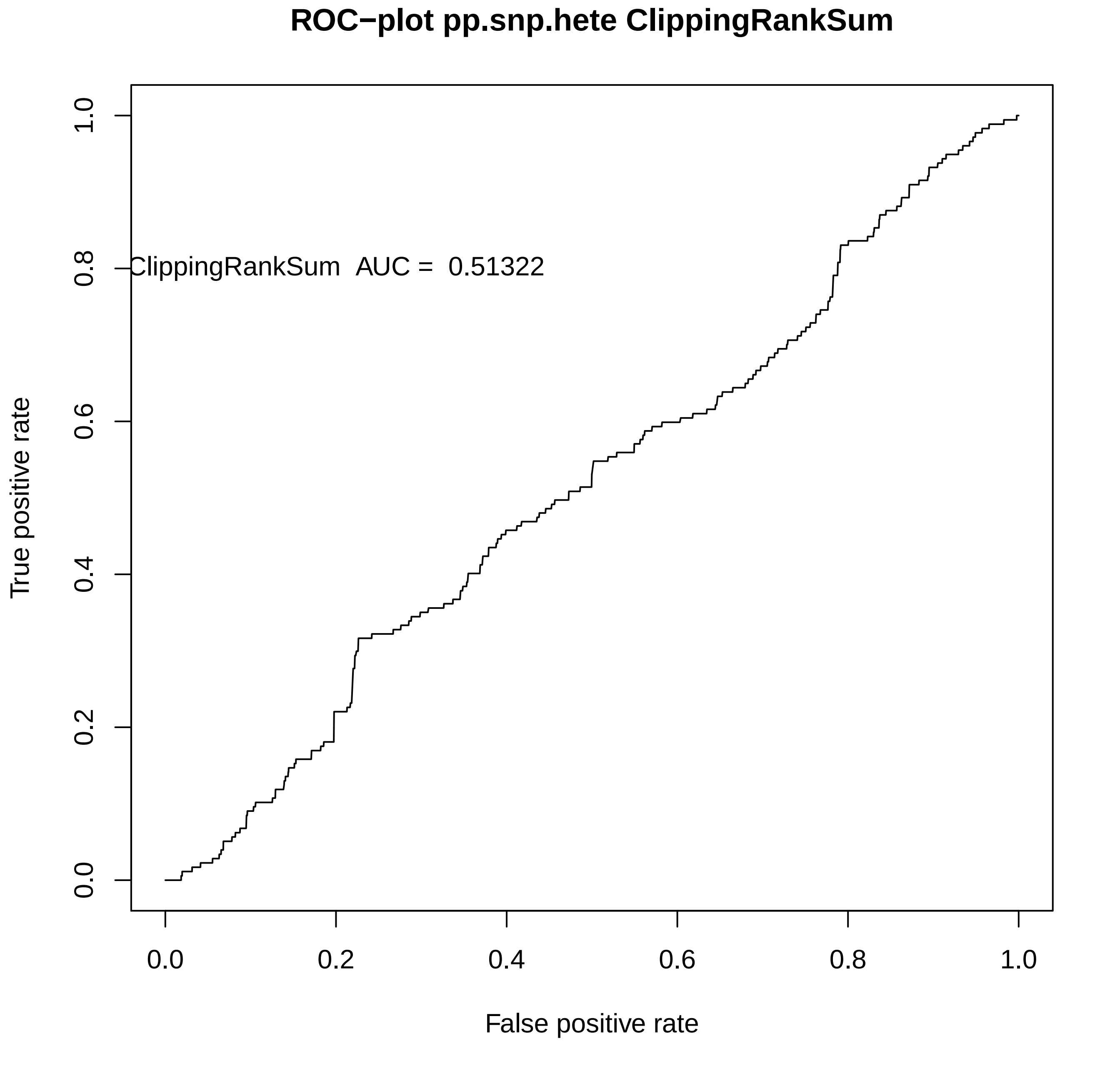

ROC−plot pp.snp.hete ClippingRankSum
ClippingRankSum AUC = 0.51322
True positive rate
False positive rate
0.0
0.2
0.4
0.6
0.8
1.0

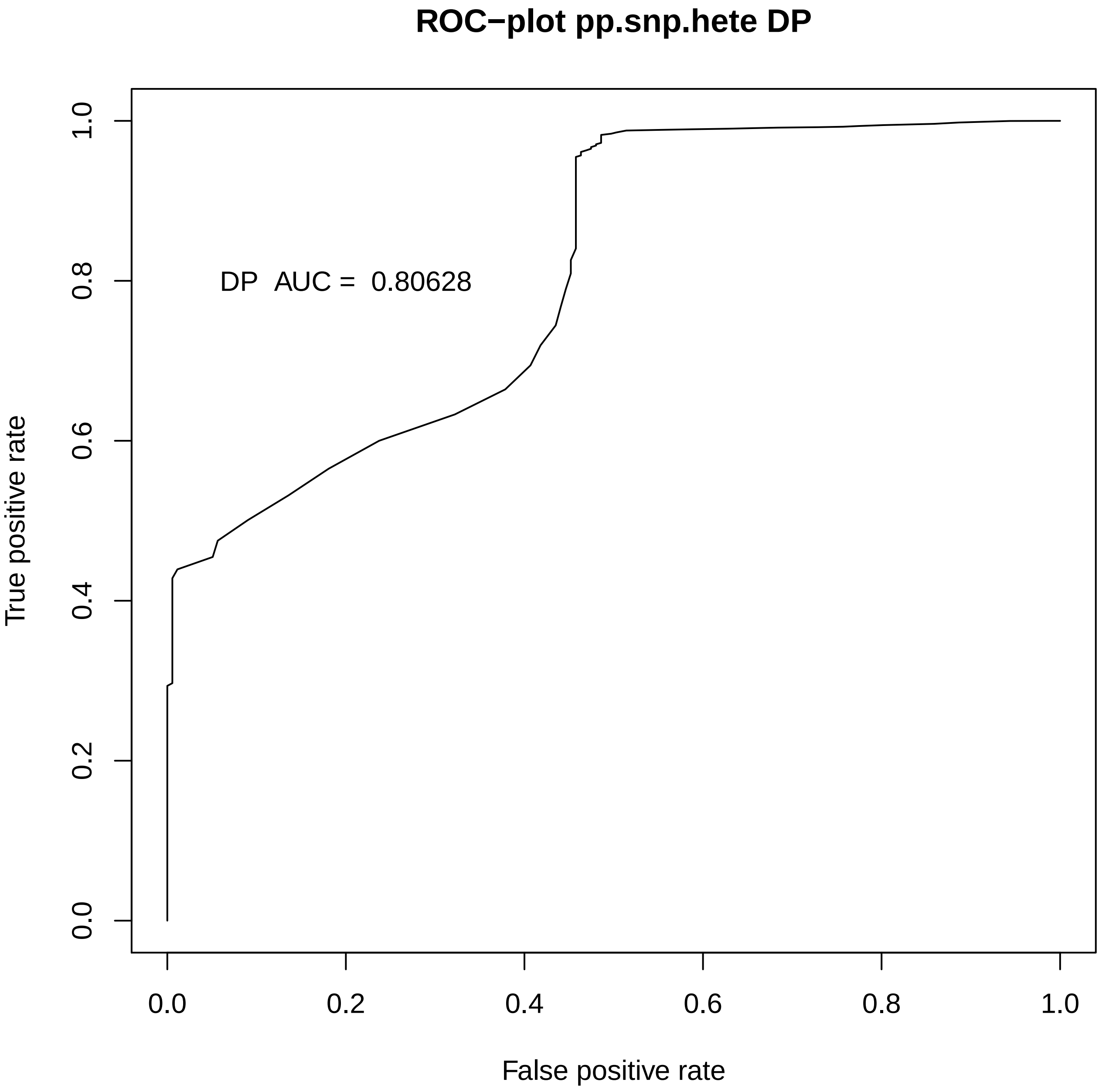
ROC−plot pp.snp.hete DP
DP AUC = 0.80628
True positive rate
False positive rate
0.0
0.2
0.4
0.6
0.8
1.0
0.0
0.2
0.4
0.6
0.8
1.0

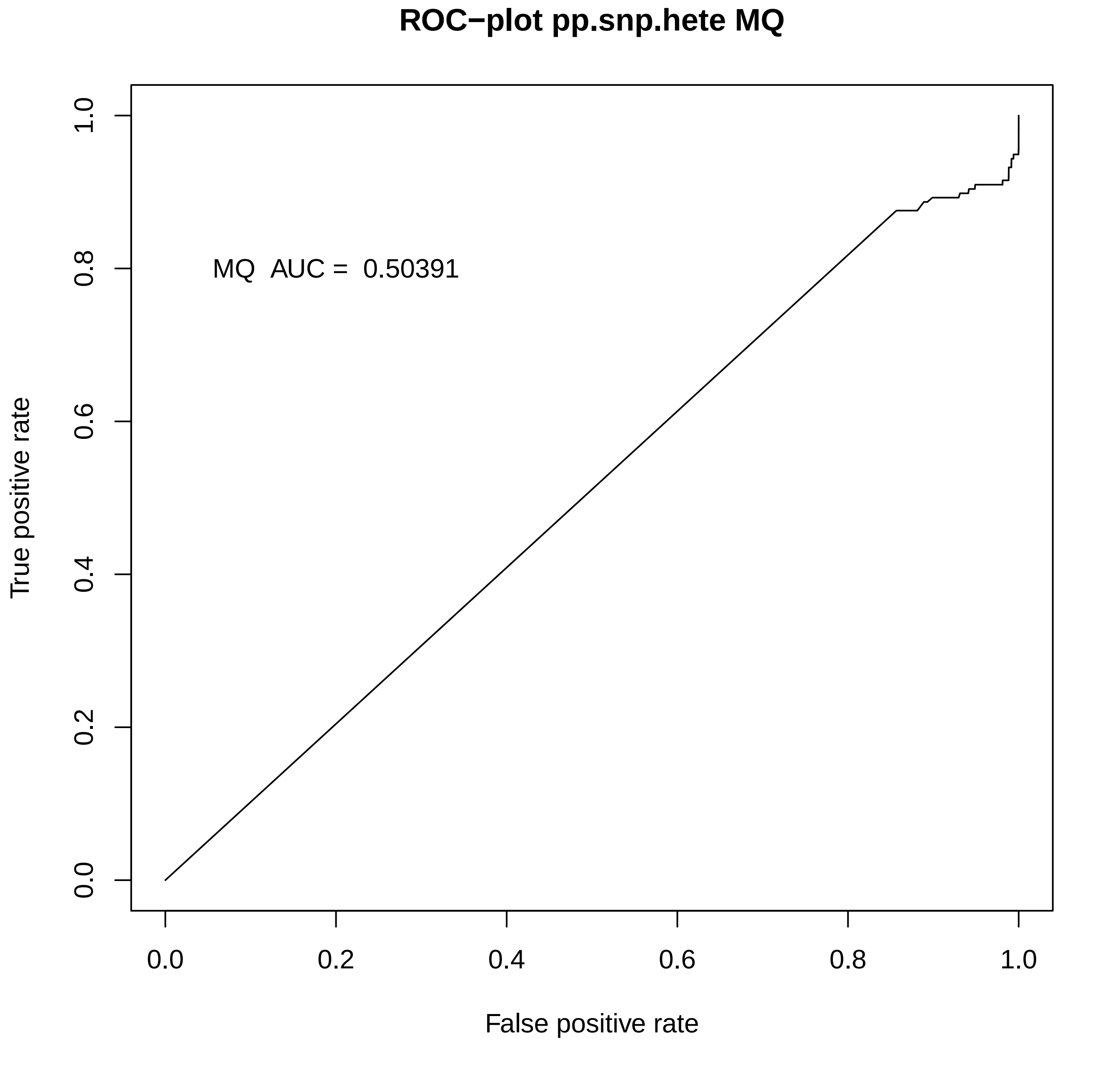
ROC−plot pp.snp.hete MQ
MQ AUC = 0.50391
True positive rate
False positive rate
0.0
0.2
0.4
0.6
0.8
1.0

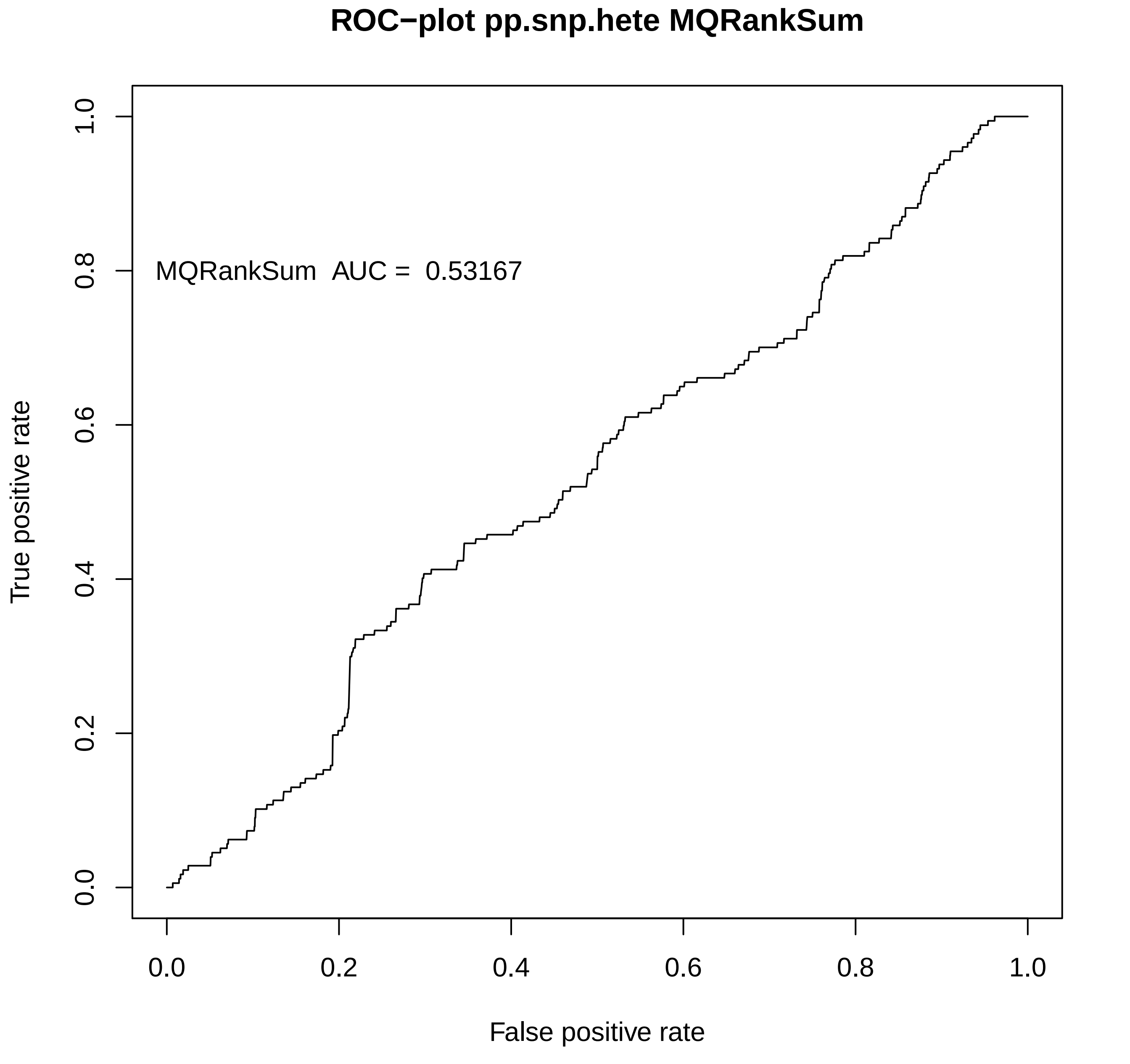
ROC−plot pp.snp.hete MQRankSum
MQRankSum AUC = 0.53167
True positive rate
False positive rate
0.0
0.2
0.4
0.6
0.8
1.0

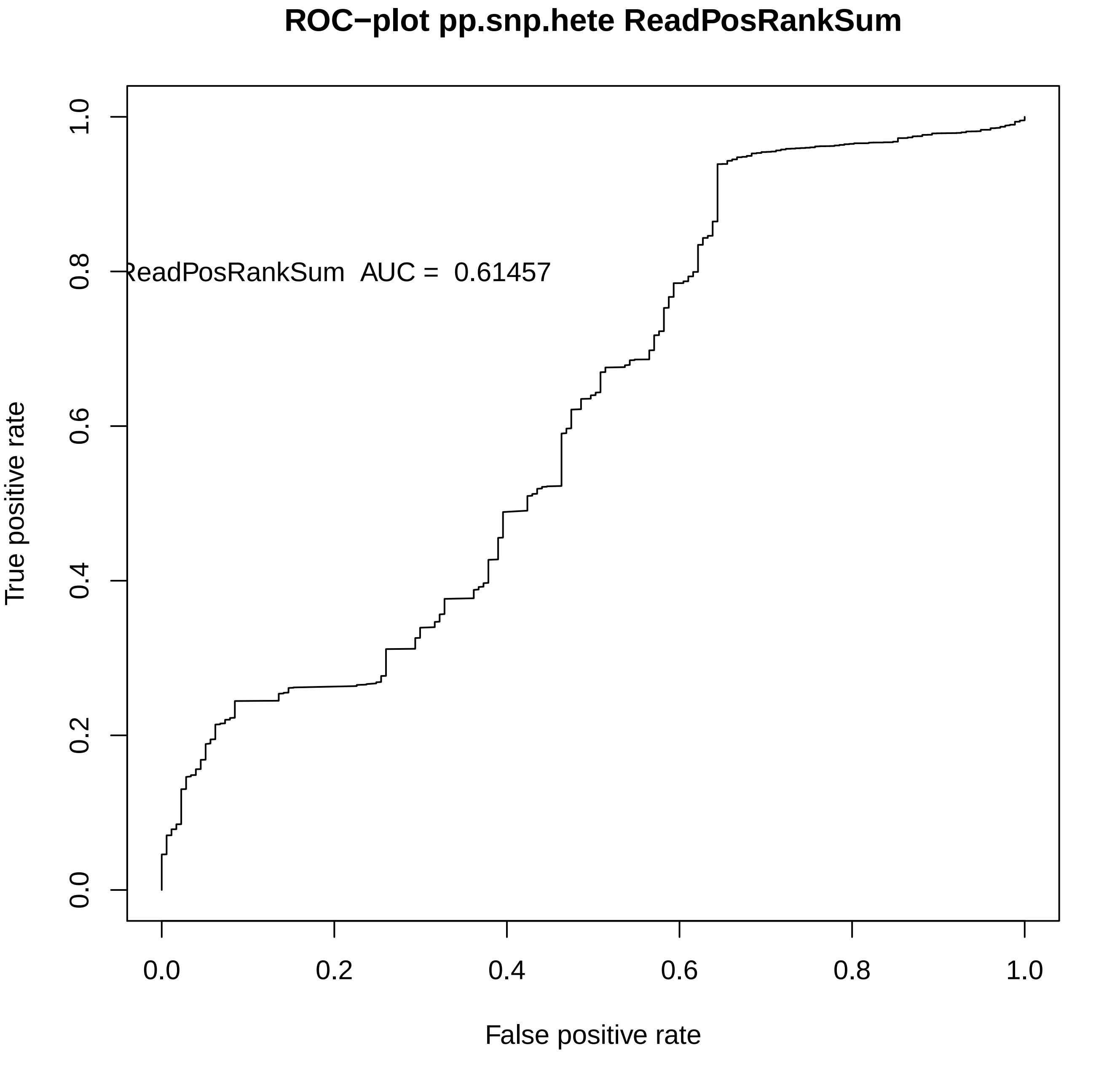

ROC−plot pp.snp.hete ReadPosRankSum
ReadPosRankSum AUC = 0.61457
True positive rate
False positive rate
0.0
0.2
0.4
0.6
0.8
1.0

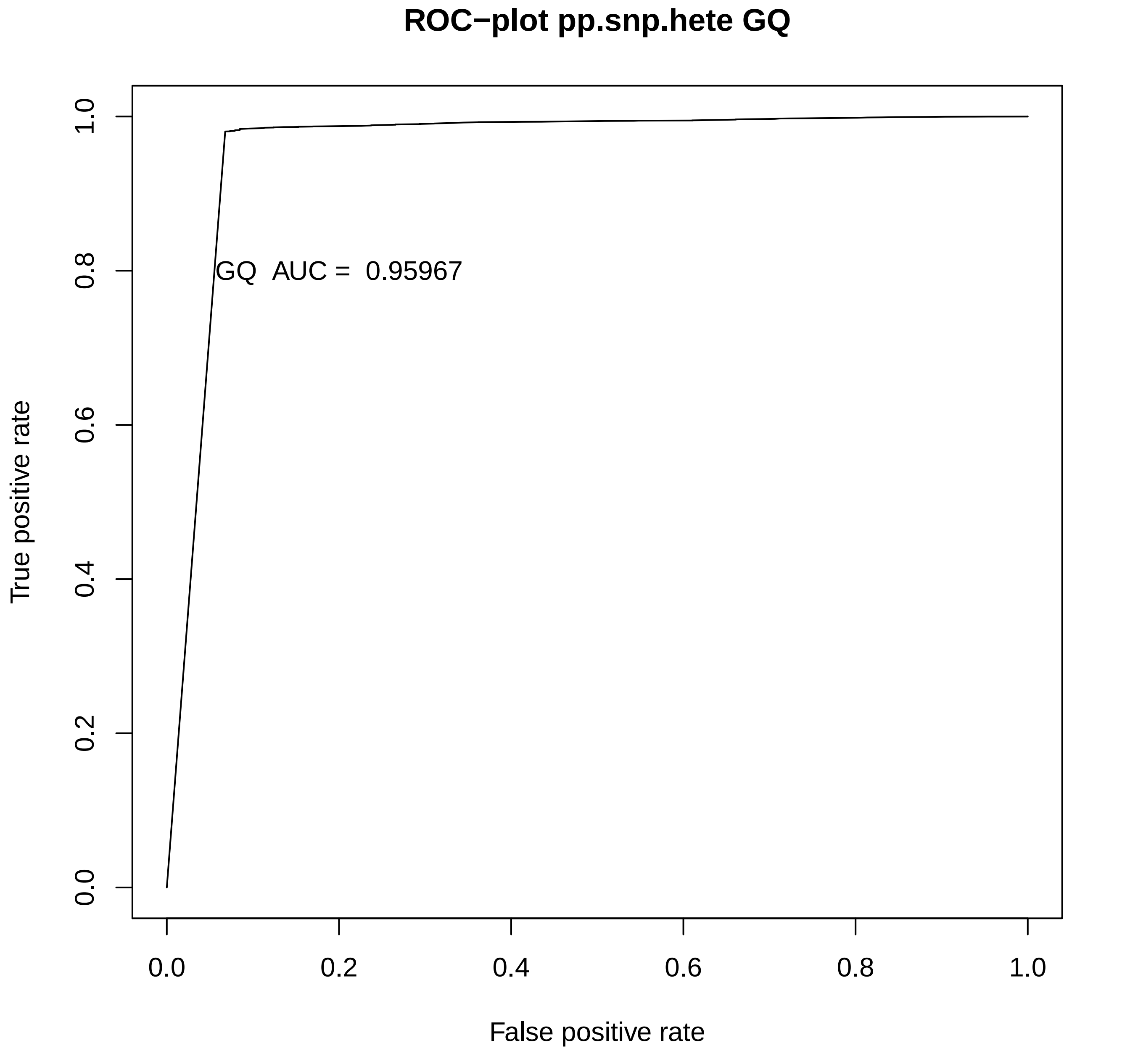
ROC−plot pp.snp.hete GQ
GQ AUC = 0.95967
True positive rate
False positive rate
0.0
0.2
0.4
0.6
0.8
1.0

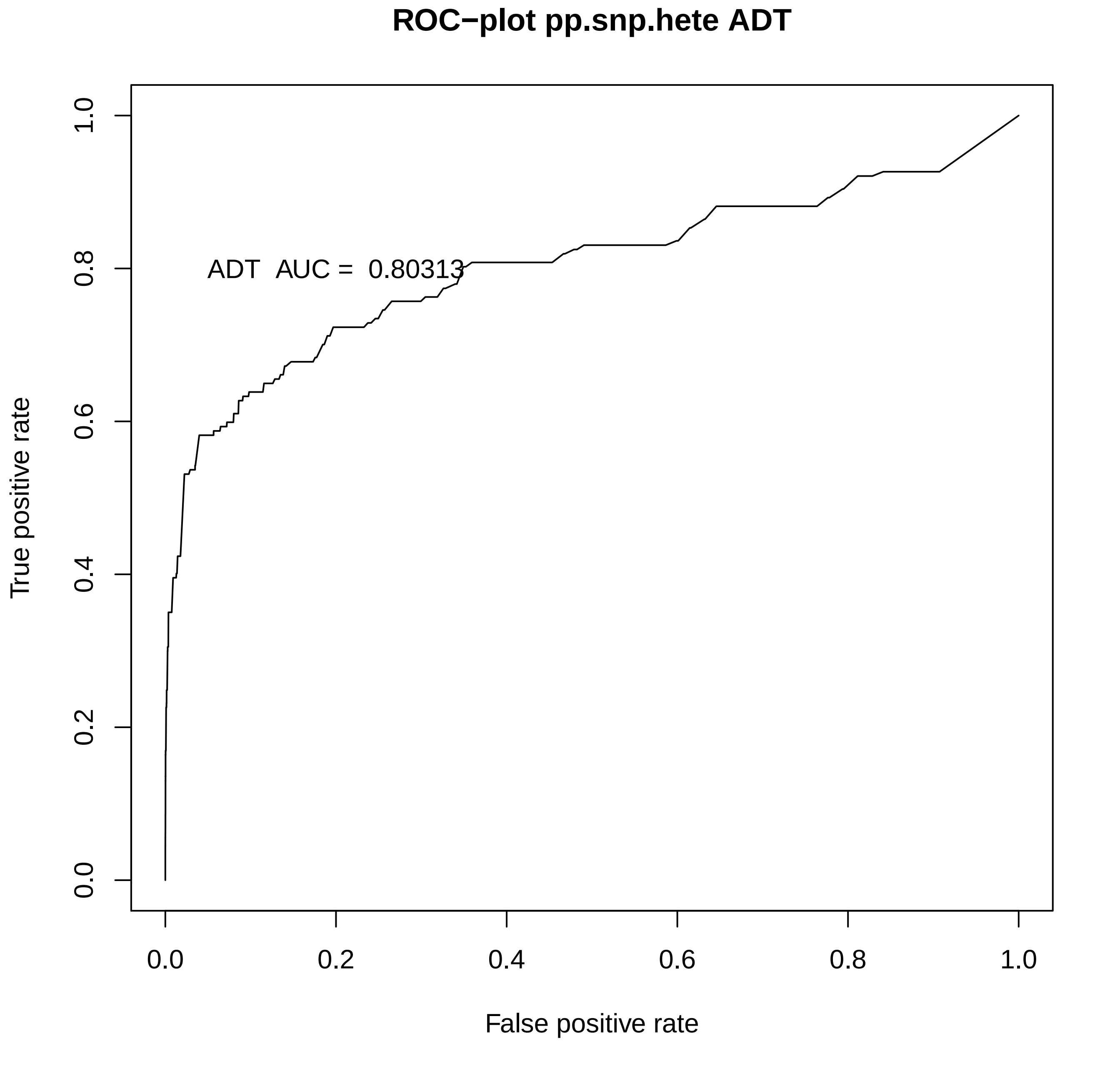

ROC−plot pp.snp.hete ADT
ADT AUC = 0.80313
True positive rate
False positive rate
0.0
0.2
0.4
0.6
0.8
1.0

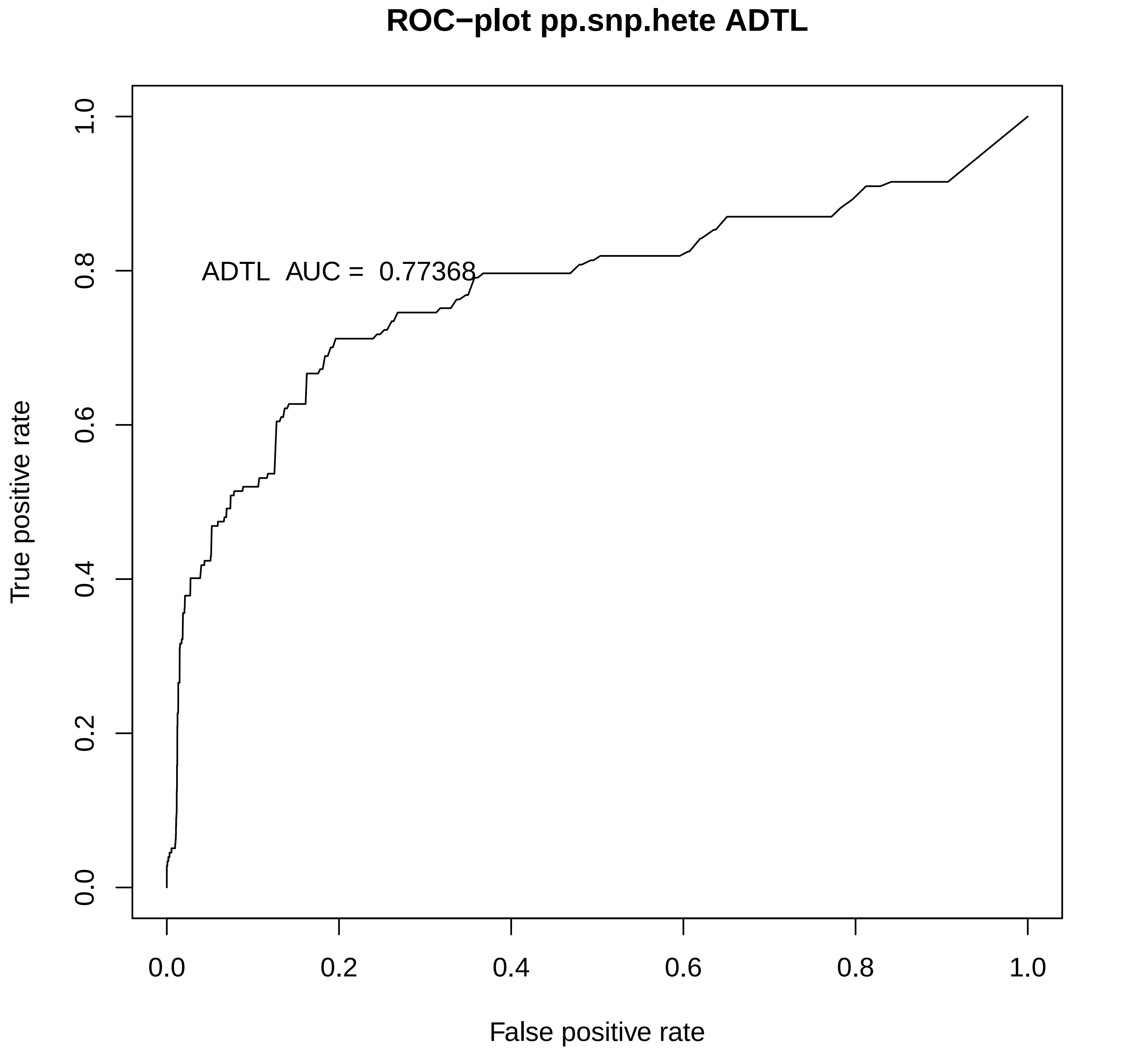

ROC−plot pp.snp.hete ADTL
ADTL AUC = 0.77368
True positive rate
False positive rate
0.0
0.2
0.4
0.6
0.8
1.0

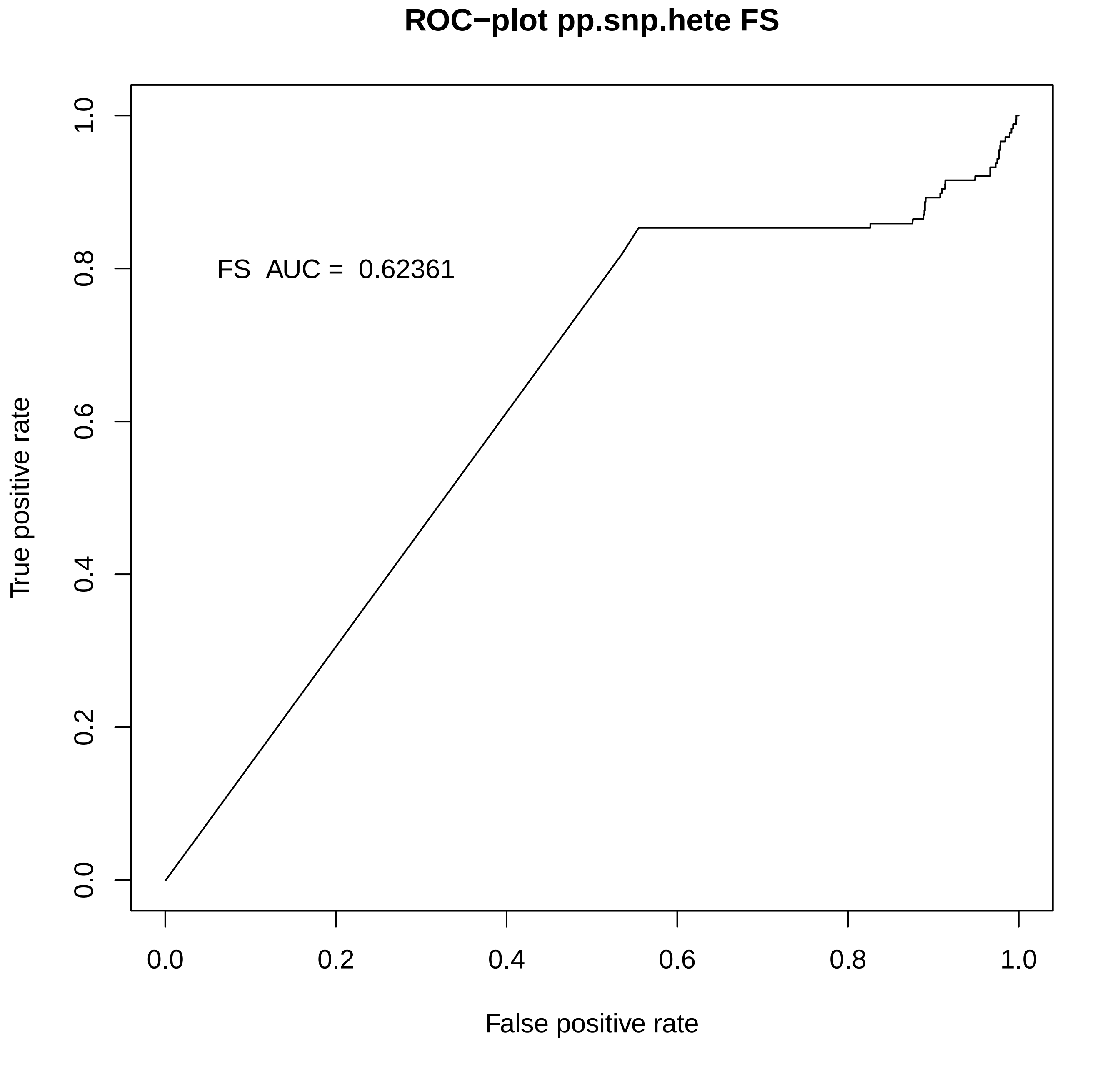

ROC−plot pp.snp.hete FS
FS AUC = 0.62361
True positive rate
False positive rate
0.0
0.2
0.4
0.6
0.8
1.0

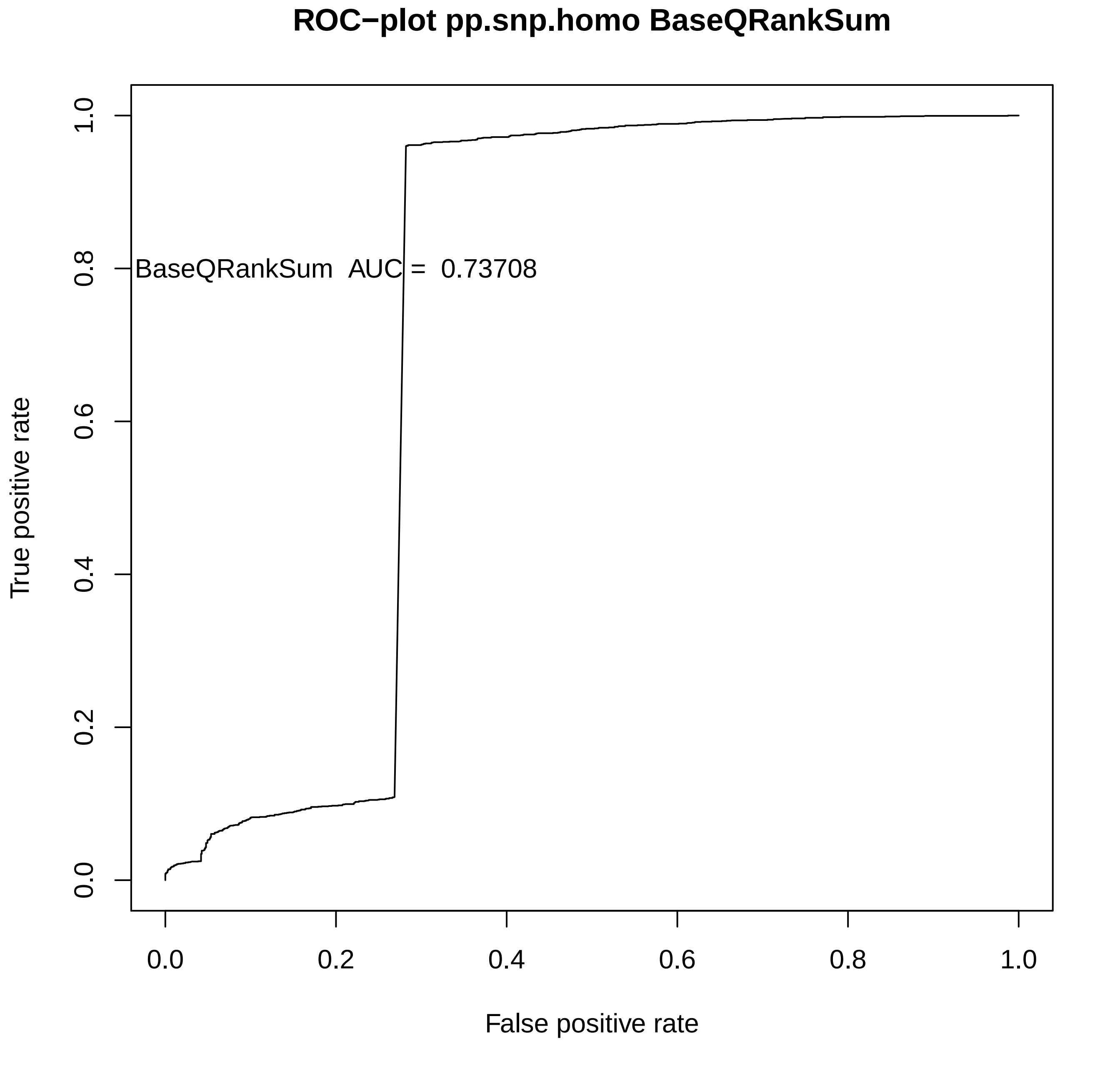

ROC−plot pp.snp.homo BaseQRankSum
BaseQRankSum AUC = 0.73708
True positive rate
False positive rate
0.0
0.2
0.4
0.6
0.8
1.0

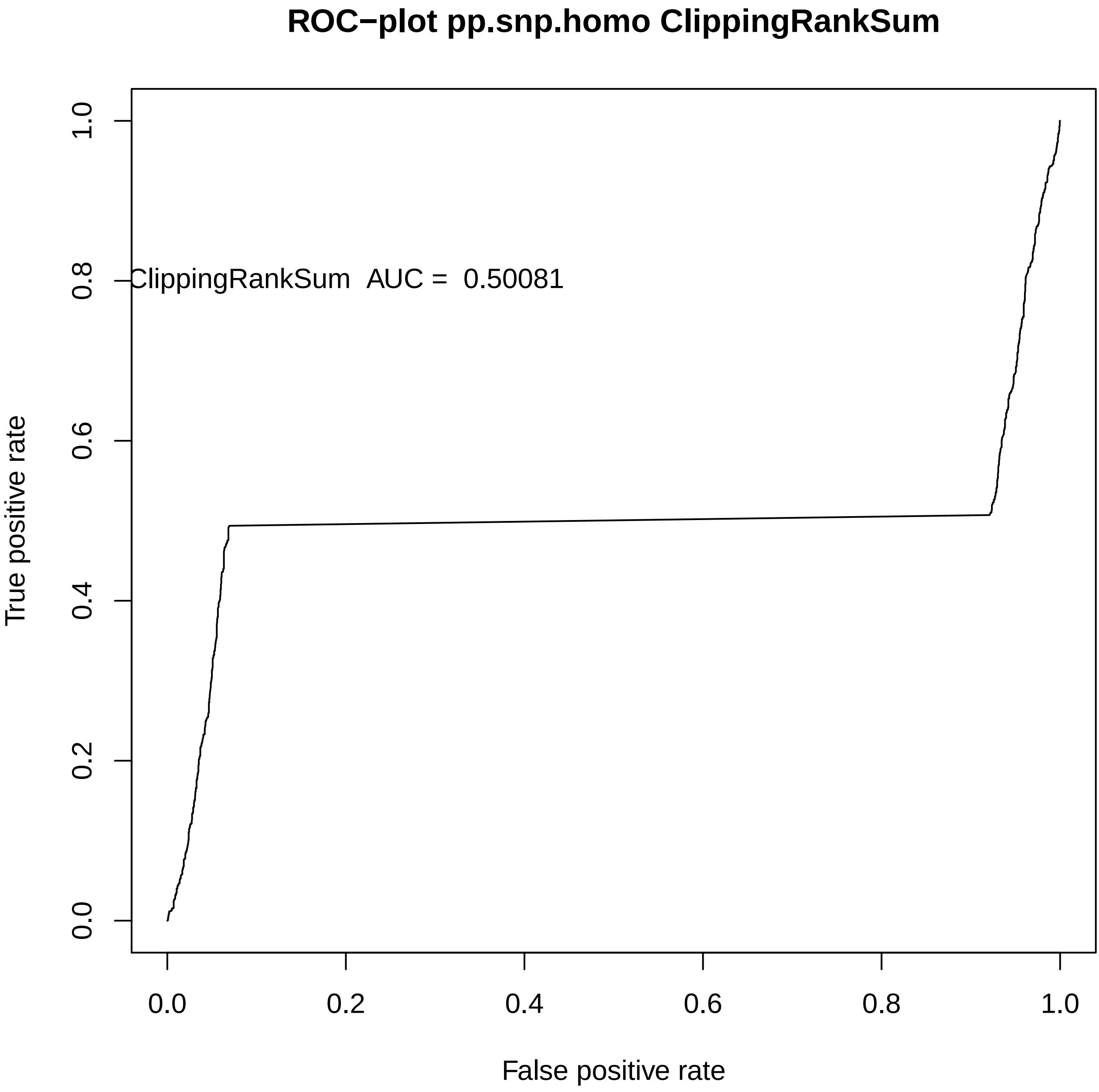
ROC−plot pp.snp.homo ClippingRankSum
ClippingRankSum AUC = 0.50081
True positive rate
False positive rate
0.0
0.2
0.4
0.6
0.8
1.0

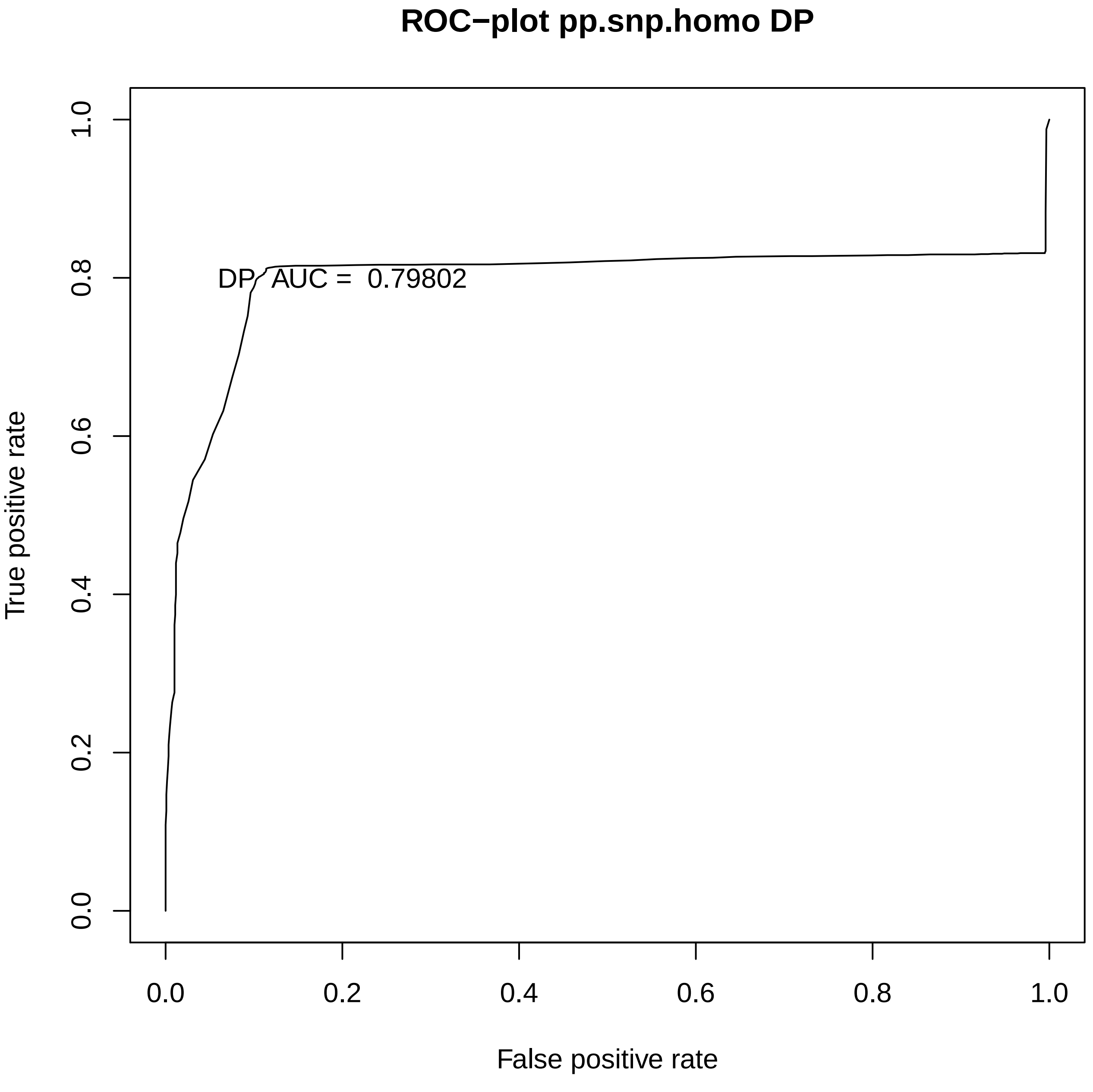

ROC−plot pp.snp.homo DP
DP AUC = 0.79802
True positive rate
False positive rate
0.0
0.2
0.4
0.6
0.8
1.0

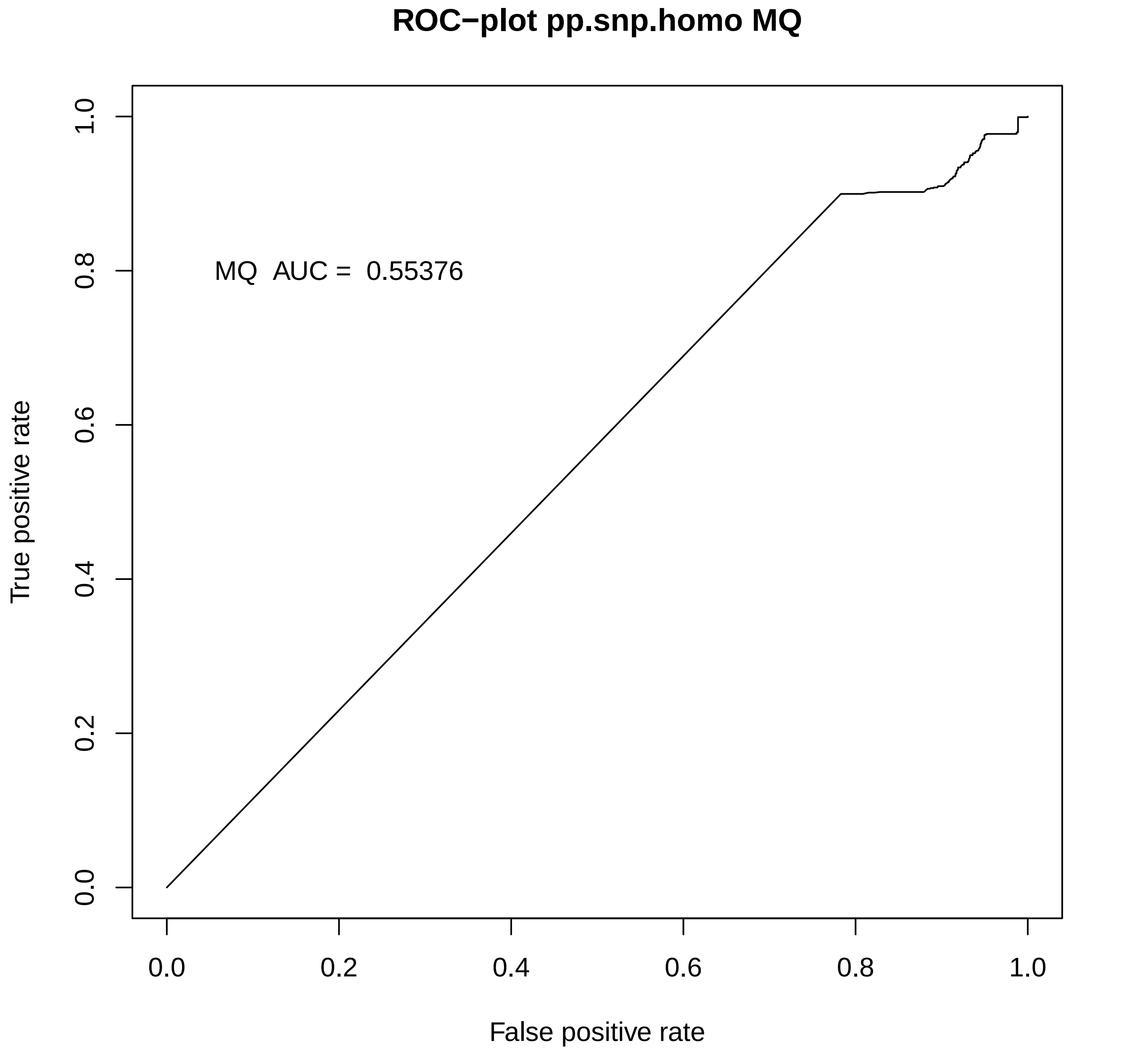

ROC−plot pp.snp.homo MQ
MQ AUC = 0.55376
True positive rate
False positive rate
0.0
0.2
0.4
0.6
0.8
1.0

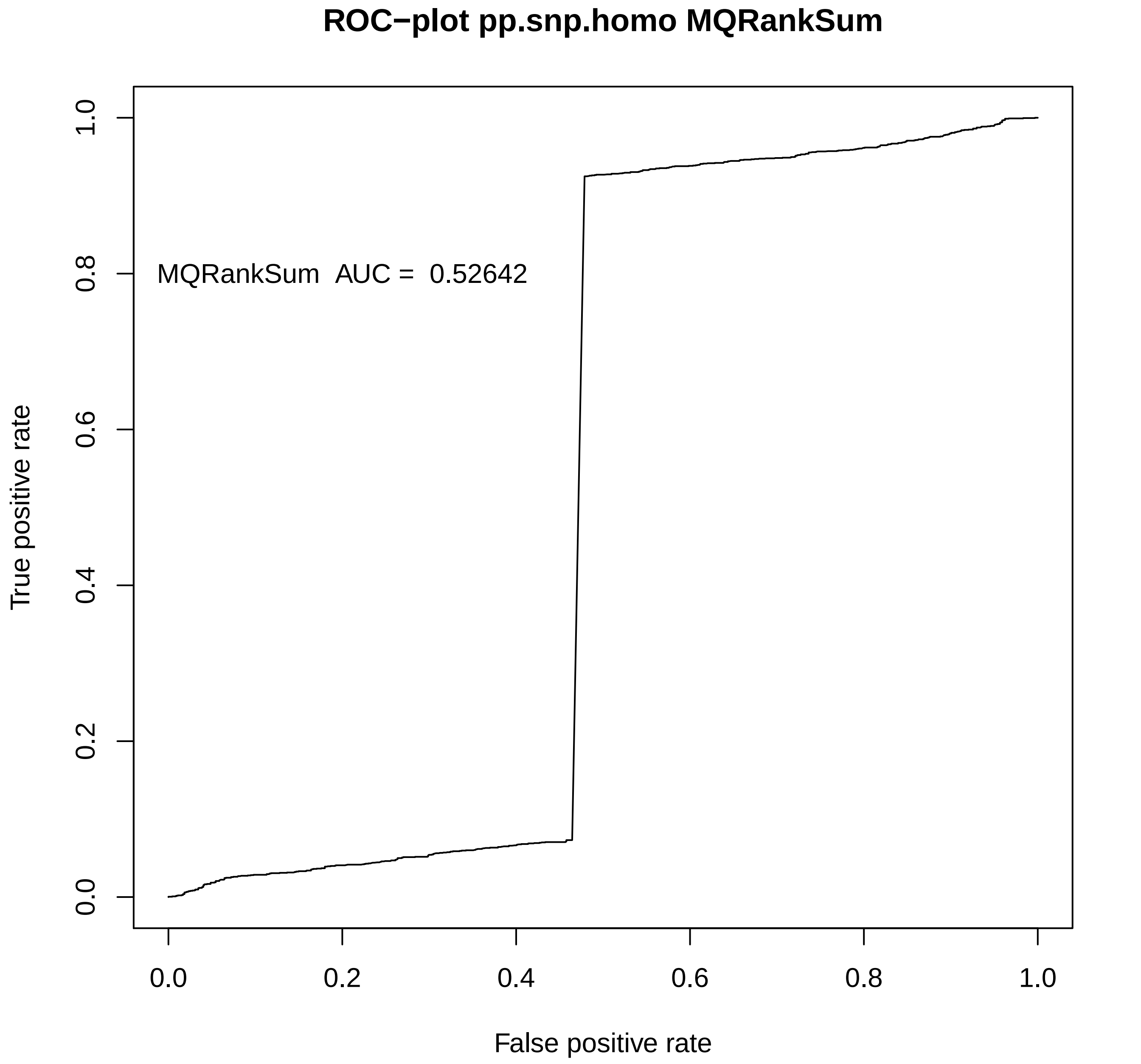
ROC−plot pp.snp.homo MQRankSum
MQRankSum AUC = 0.52642
True positive rate
False positive rate
0.0
0.2
0.4
0.6
0.8
1.0

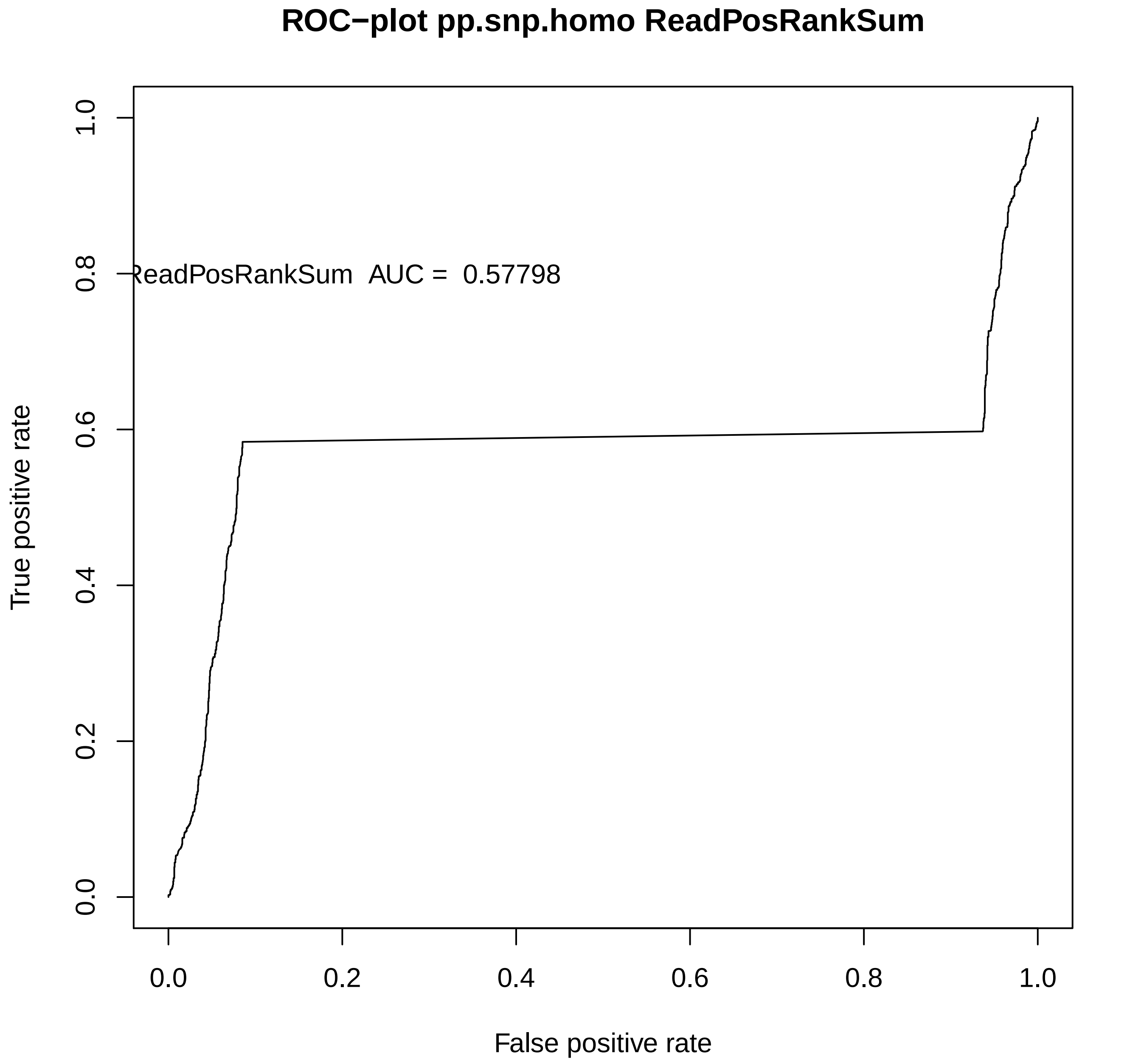

ROC−plot pp.snp.homo ReadPosRankSum
ReadPosRankSum AUC = 0.57798
True positive rate
False positive rate
0.0
0.2
0.4
0.6
0.8
1.0

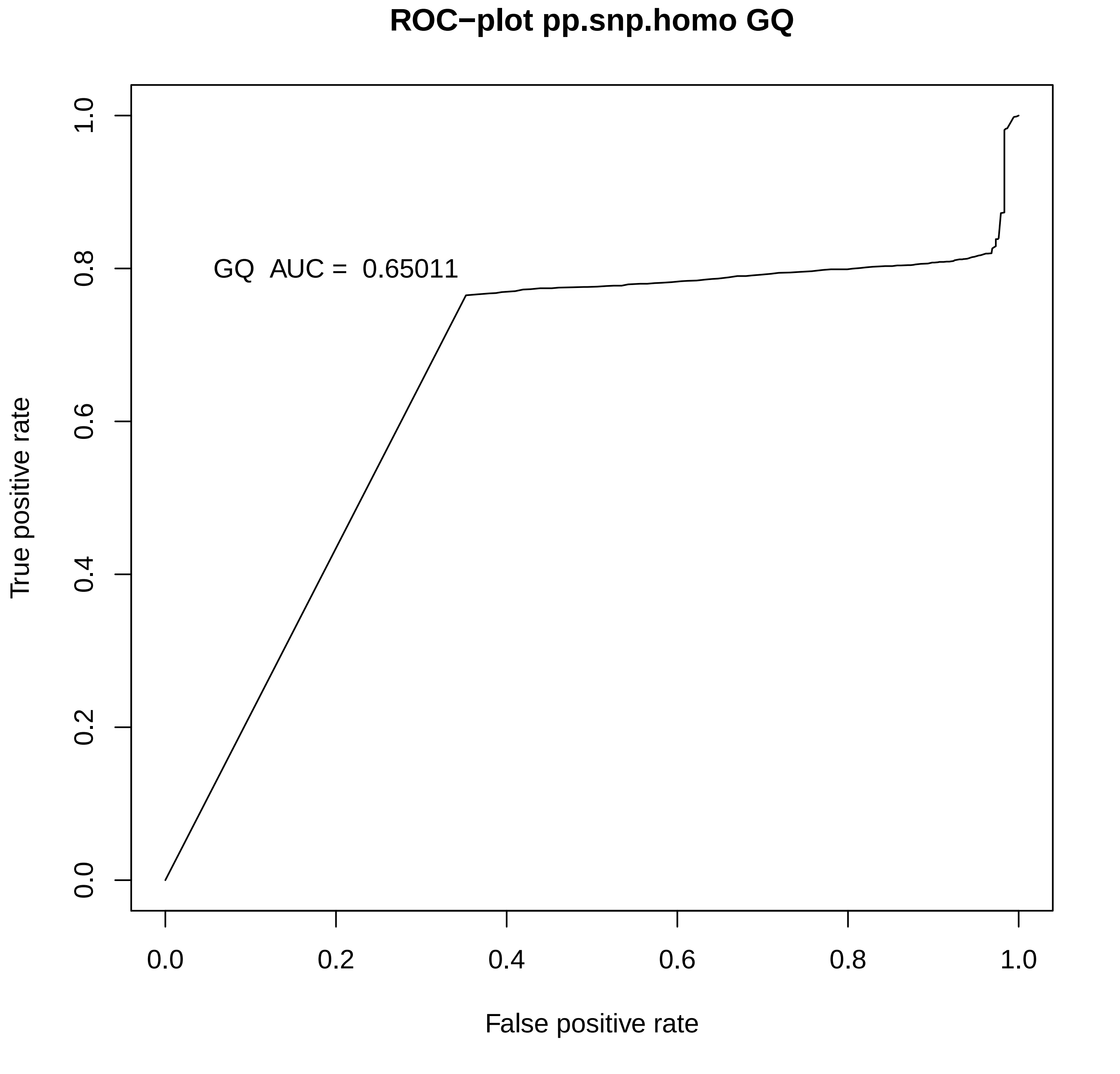
ROC−plot pp.snp.homo GQ
GQ AUC = 0.65011
True positive rate
False positive rate
0.0
0.2
0.4
0.6
0.8
1.0

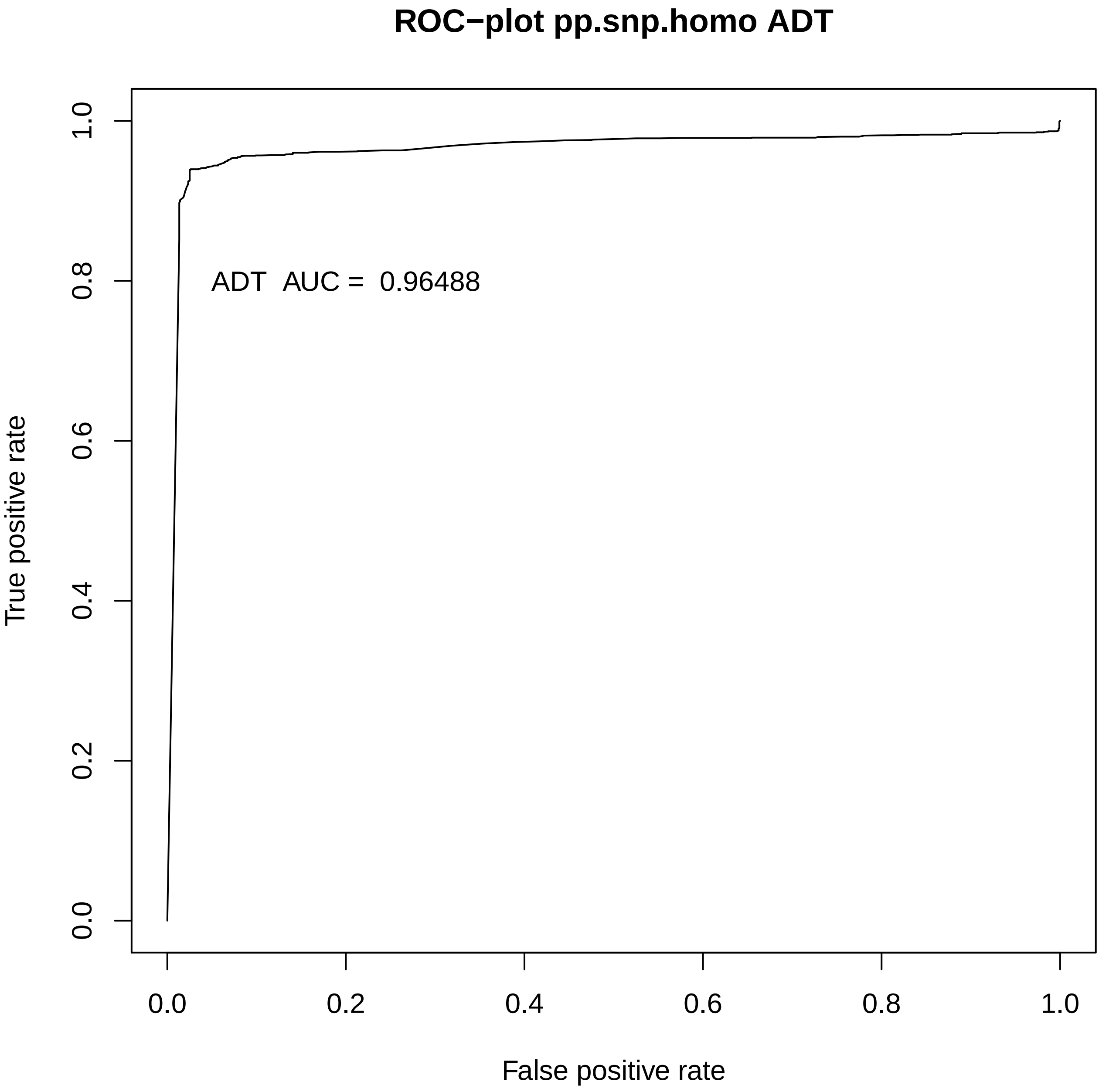
ROC−plot pp.snp.homo ADT
ADT AUC = 0.96488
True positive rate
False positive rate
0.0
0.2
0.4
0.6
0.8
1.0

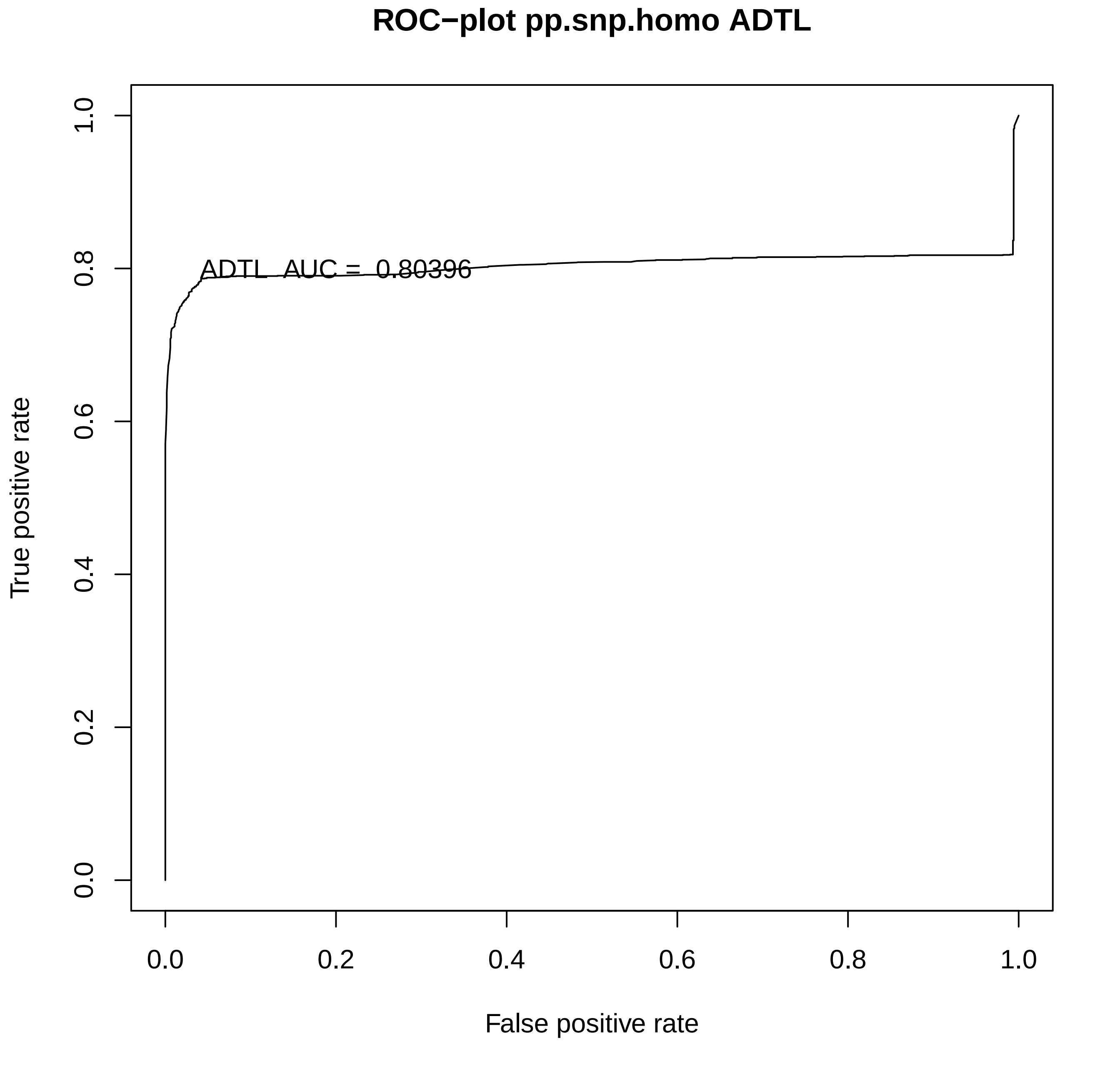
ROC−plot pp.snp.homo ADTL
ADTL AUC = 0.80396
True positive rate
False positive rate
0.0
0.2
0.4
0.6
0.8
1.0

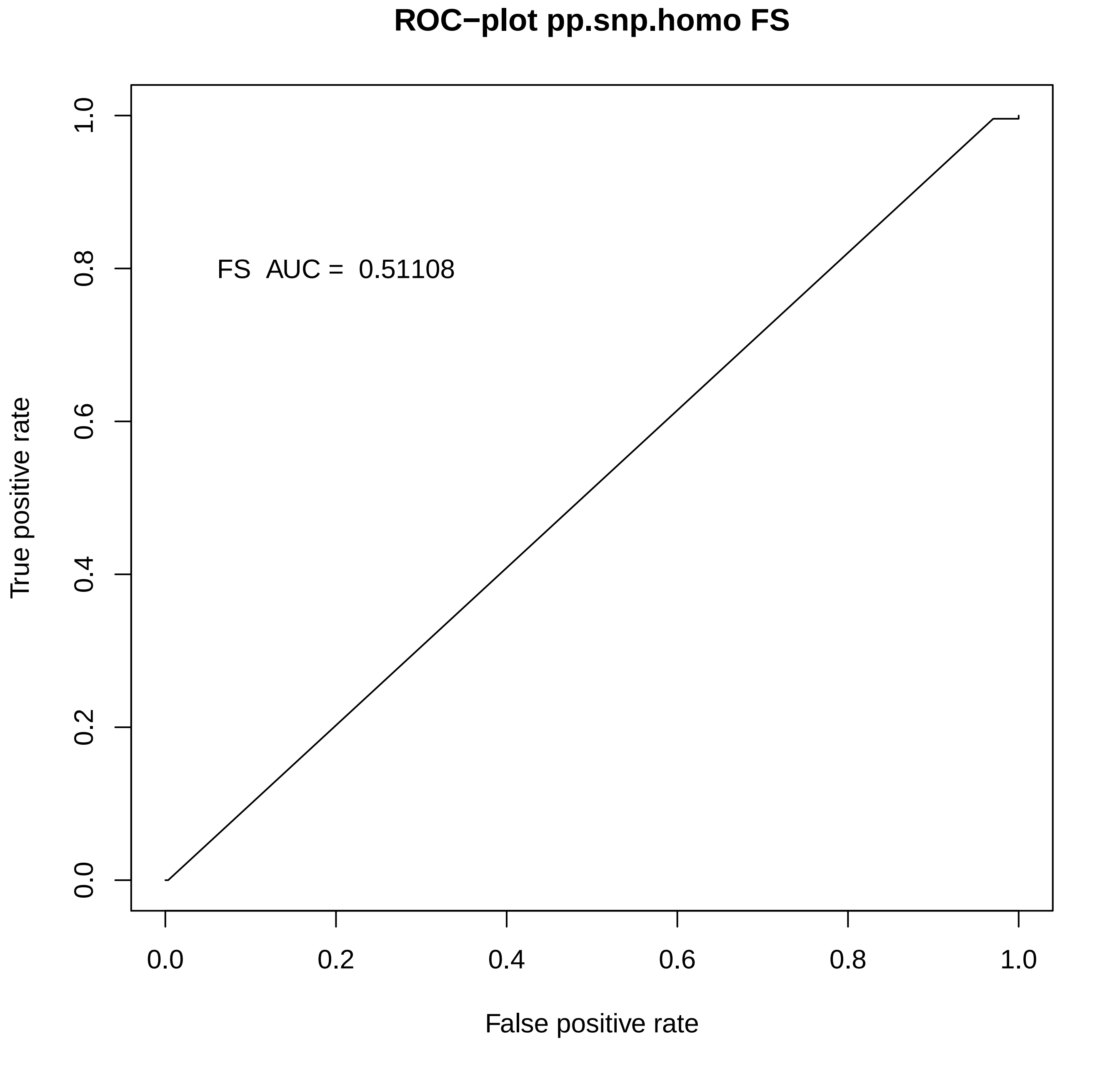

ROC−plot pp.snp.homo FS
FS AUC = 0.51108
True positive rate
False positive rate
0.0
0.2
0.4
0.6
0.8
1.0
