# Supplementary material for: GATK hard filtering: tunable parameters to improve variant calling for next generation sequencing targeted gene panel data
Source: BMC Bioinformatics. 2017 Mar 23;18(Suppl 5):119. doi: 10.1186/s12859-017-1537-8 (PMC5374681; doi:10.1186/s12859-017-1537-8)

HC SNV Heterozygous

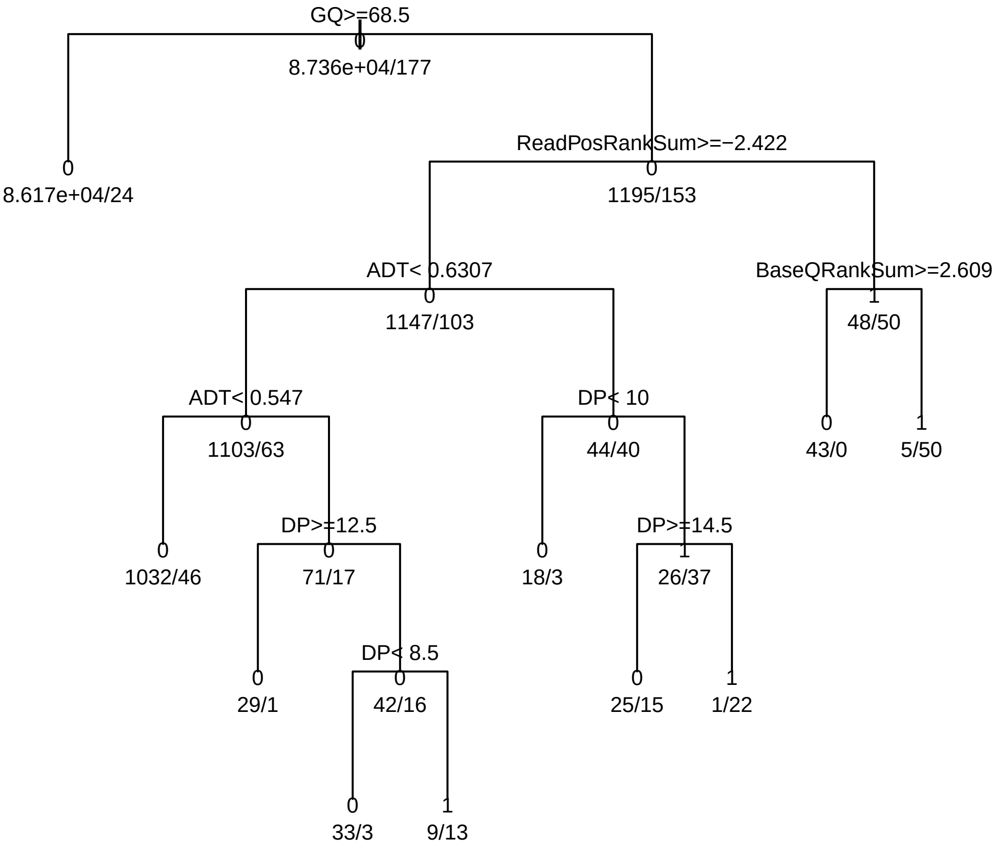

HC SNV Homozygous

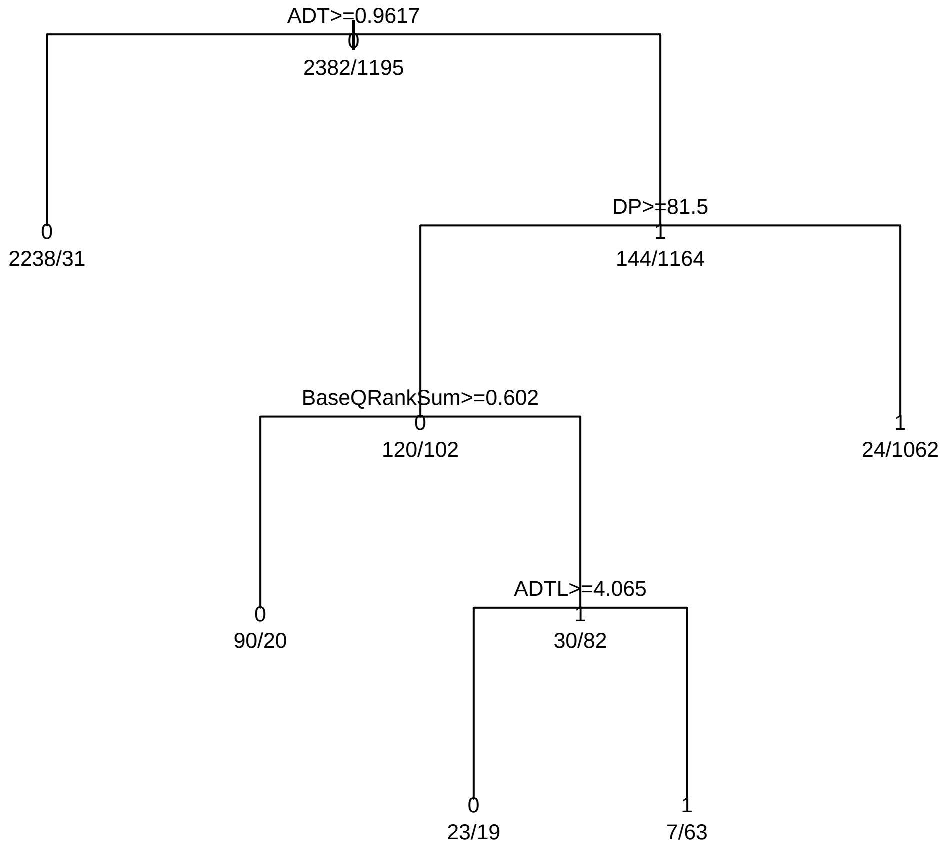

HC Indel Heterozygous

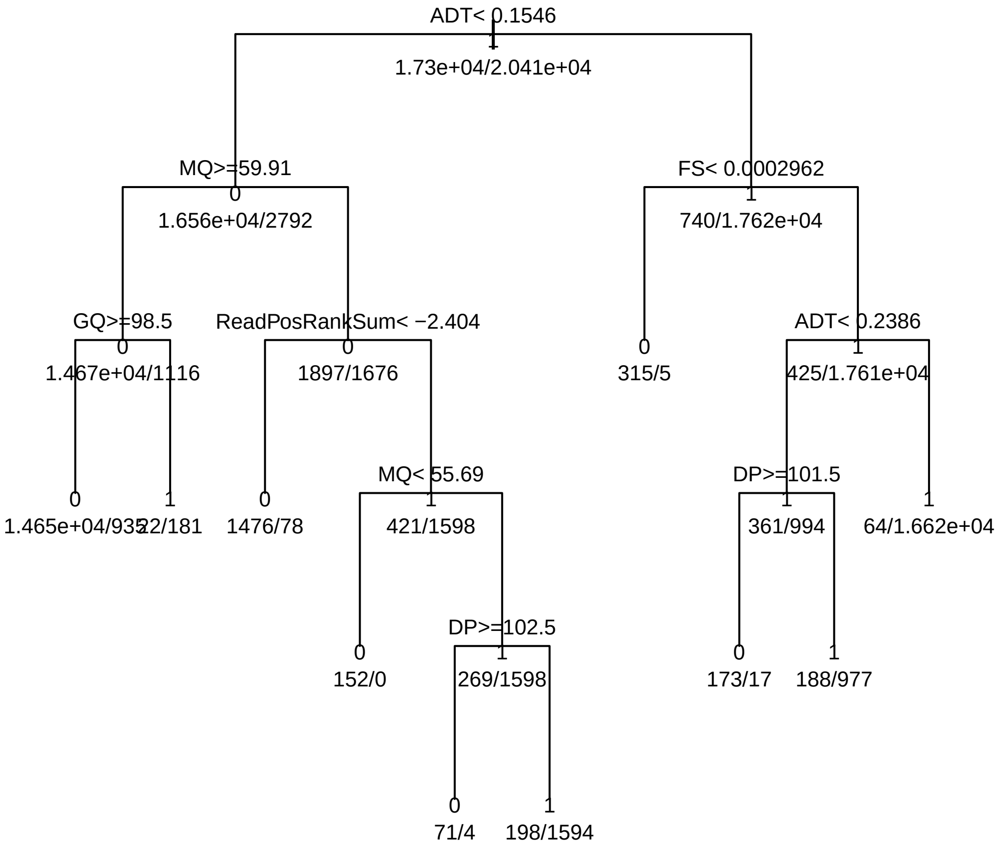

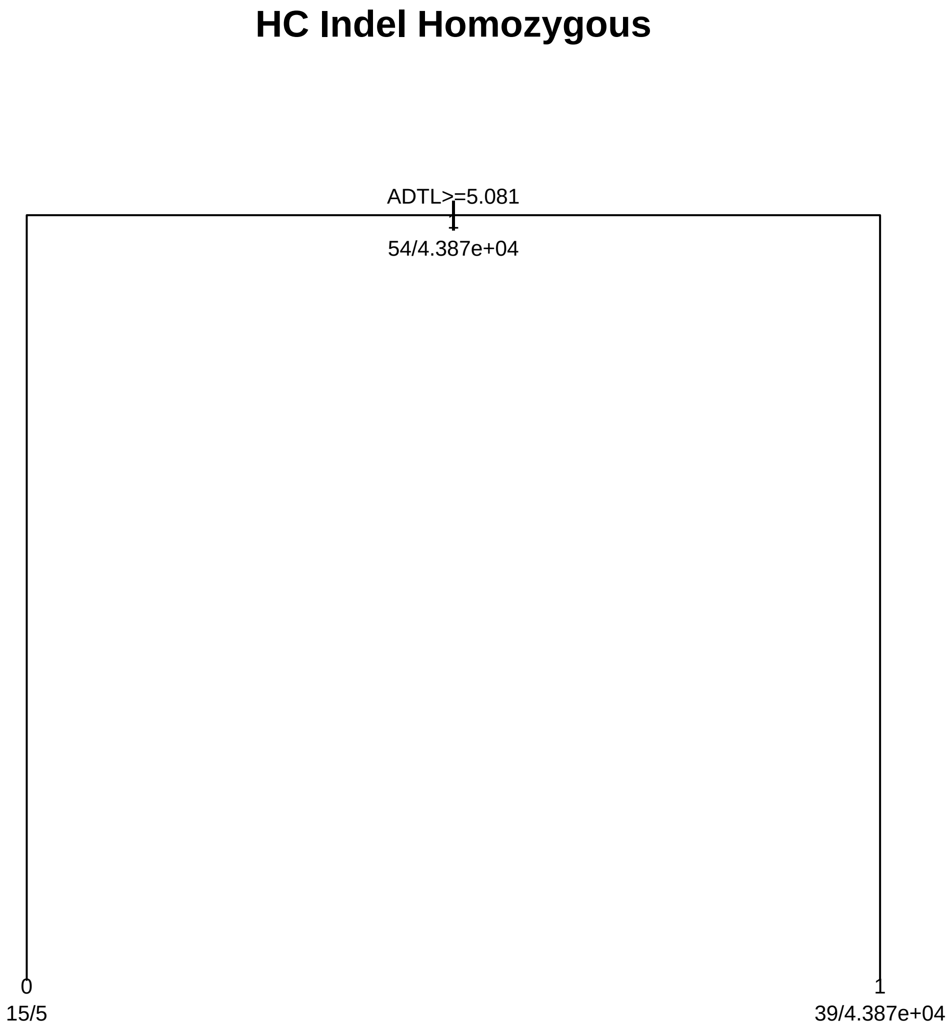
HC Indel Homozygous
ADTL>=5.081
1
54/4.387e+04
0
15/5
1
39/4.387e+04

LC SNV Heterozygous

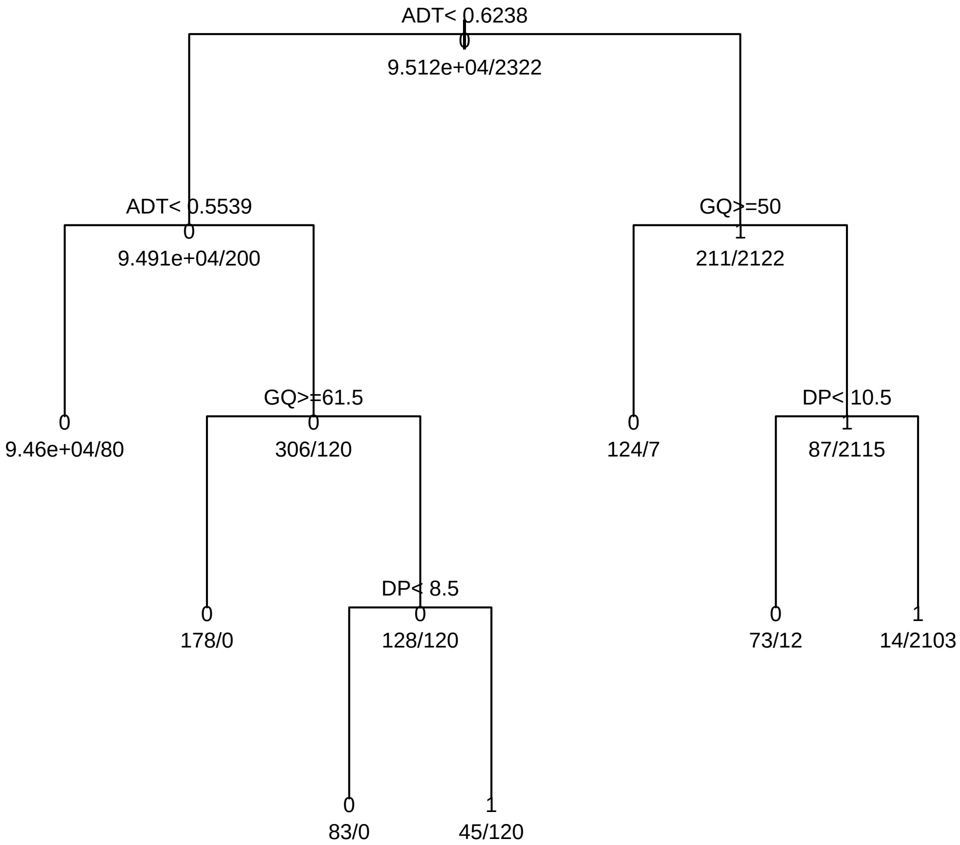

LC SNV Homozygous

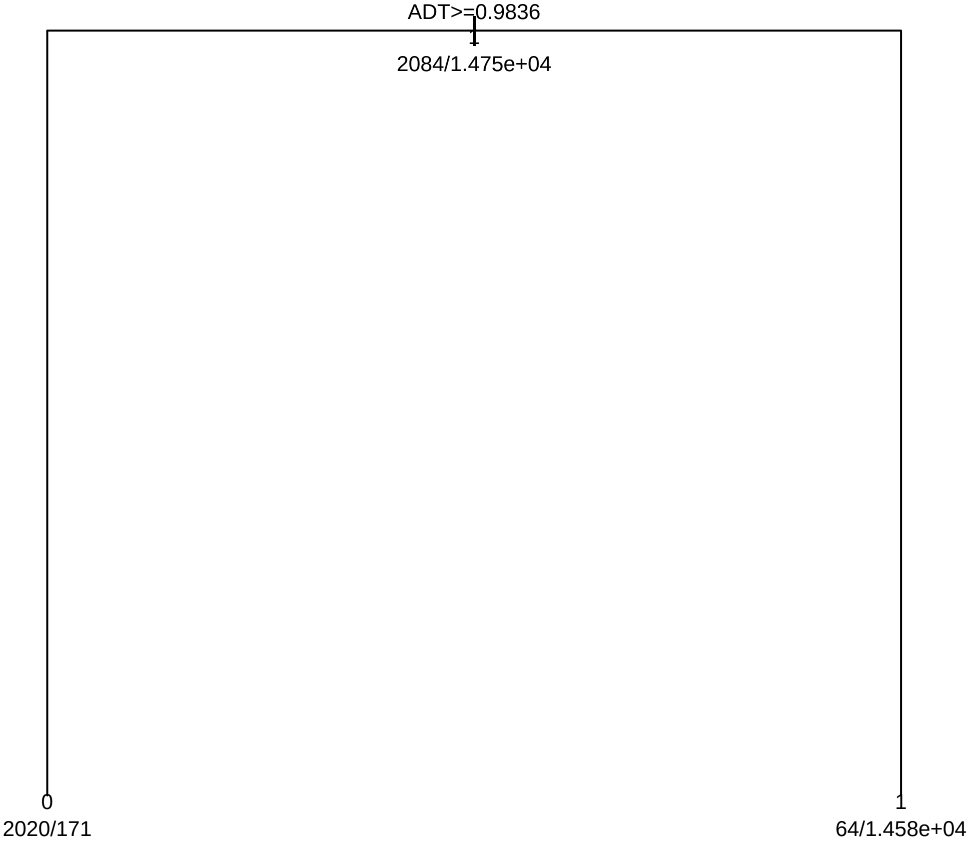

LC Indel Heterozygous

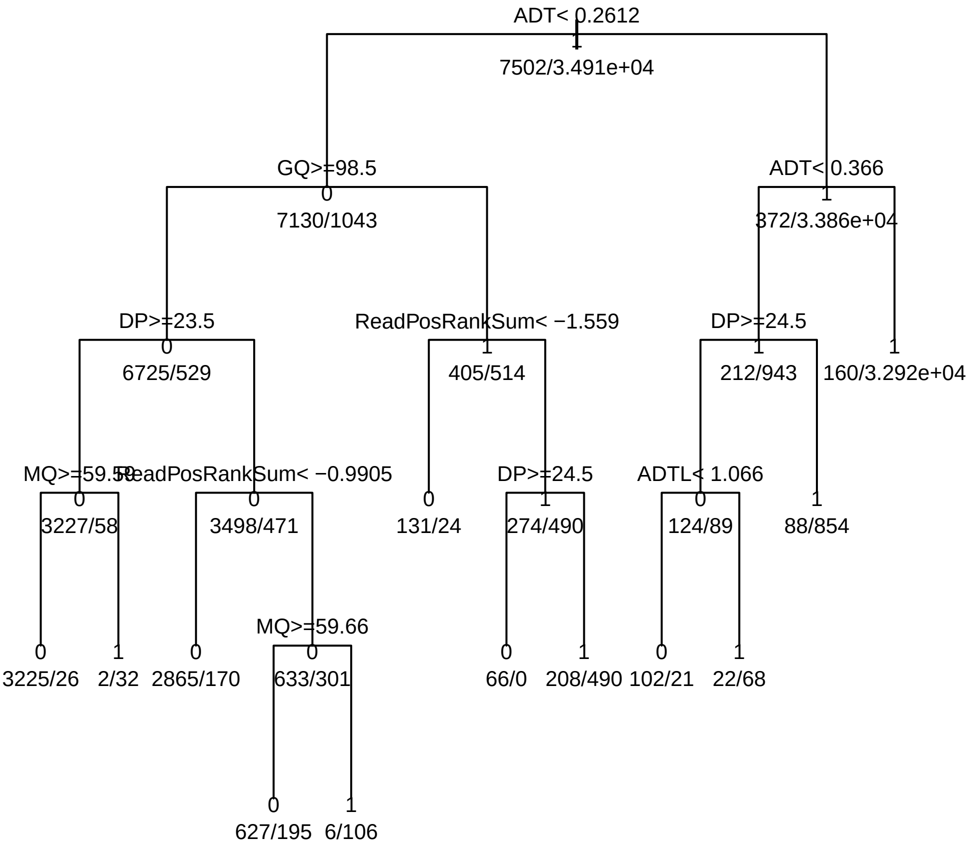

Supplement: Supplementary file 3 — Classification trees to set filter rules. (PDF 9 kb) [file 12859_2017_1537_MOESM3_ESM.pdf]
